# Individual versus group-based interventions: a systematic review and meta-analysis of physical activity, functional, psychosocial and health outcomes

**DOI:** 10.1038/s41562-026-02429-0

**Published:** 2026-04-15

**Authors:** Marlene Kritz, Hugh Riddell, Daryn Olsen, Samantha M. Harden, Shauna M. Burke, Nikos Ntoumanis, Cecilie Thøgersen-Ntoumani

**Affiliations:** 1 Department for Economy and Health, University for Continuing Education, Krems, Austria; 2 Curtin School of Population Health, Faculty of Health Sciences, Curtin University, Perth, Western Australia Australia; 3 Department of Human Nutrition, Foods, and Exercise, Virginia Tech, Blacksburg, VA USA; 4 School of Health Studies, Faculty of Health Sciences, Western University, London, Ontario Canada; 5 Children’s Health Research Institute, London, Ontario Canada; 6 Danish Centre for Motivation and Behaviour Science, University of Southern Denmark, Odense, Denmark; 7 School of Sport, Exercise, and Rehabilitation Sciences, University of Birmingham, Birmingham, UK

**Keywords:** Public health, Human behaviour

## Abstract

Social influences, including group dynamics, social norms and peer support, are assumed to influence physical activity (PA). A previous systematic review and meta-analysis found that ‘true groups’ (that is, those applying group dynamics principles) had advantages over individual PA interventions. Given technological advances and virtual platforms, we updated previous findings. Systematic searches of electronic databases were completed on 19 March 2024. A meta-analysis of 71 studies (523 effect sizes) compared individual and group-based PA interventions across behavioural, functional (for example, strength and flexibility), psychosocial (for example, quality of life and loneliness) and health outcomes (for example, VO_2_max). Using a three-level random effects model, group-based conditions showed a small but non-significant advantage for PA (*n* = 22,042; *g* = 0.086, 95% CI (−0.061, 0.233), *P* = 0.249), psychosocial outcomes (*n* = 18,223; *g* = 0.292, 95% CI (−0.171, 0.755), *P* = 0.214) and health outcomes (*n* = 31,607; *g* = 0.125, 95% CI (−0.023, 0.272), *P* = 0.096). For functional outcomes, a significant advantage for group-based interventions emerged after outlier removal (*n* = 14,429; *g* = 0.164, 95% CI (0.032, 0.297), *P* = 0.015). Moderation analysis indicated that different group types produced comparable effects, except that ‘true groups’ were associated with a larger effect size for health outcomes. Both individual and group-based approaches (in-person or online) may be effective. Future research should explore mechanisms that enhance their impact across populations and settings. This review was prospectively registered with PROSPERO (CRD42021271452).

## Abstract (web summary)

A systematic review and meta-analysis compares the effects of individual and group-based approaches on physical activity, functional, psychosocial and health outcomes.

## Main

Regular physical activity (PA) is associated with numerous physical and mental health benefits^1–3^. To achieve these benefits, the World Health Organization recommends 150–300 min of moderate-intensity or 75–150 min of vigorous-intensity aerobic activity per week, along with muscle-strengthening exercises on 2 or more days^4^. Despite such potential health gains, many adults fail to reach the recommended guidelines for PA globally^5–7^. Even among those who join a PA programme, the majority struggle with adherence and fail to sustain changes post-programme completion^8,9^.

Understanding the efficacy of interventions aimed at promoting PA requires consideration of the contextual factors surrounding PA engagement. The social environment, encompassing social norms, social support, support networks and a sense of belonging, can exert a powerful influence on PA^10,11^. These social influences are central to numerous theories applied to PA, including group dynamics^12,13^, social identity^14,15^, self-determination^16,17^ and social cognitive theories^18,19^, commonly used to inform intervention design. For instance, group dynamics theory posits that individuals are influenced by the attitudes and behaviours of their social groups, shaping social norms and subsequently guiding behaviour. Social identity theory emphasizes how individuals derive their sense of self from group affiliations, leading them to conform to group norms and values^15^. Self-determination theory suggests that social contexts that satisfy the psychological needs for autonomy, competence and relatedness are pivotal for sustained behavioural engagement^16^. Lastly, social cognitive theory suggests that individuals model the behaviours of others, highlighting the importance of social modelling and reinforcement^18^.

While social influences can profoundly shape the way PA is pursued and/or adhered to, not all individuals are attracted to group-based PA. Indeed, many choose solitary PA, either owing to personal preference or circumstances such as the COVID-19 pandemic^20^. Individualized approaches can afford individuals autonomy and flexibility, enabling them to tailor their PA programme to their specific needs, goals and schedules. However, inherently, they may lack the support, structured feedback and guidance, external accountability and social interaction that can bolster motivation and sustained PA behaviour. Participating in group activities can yield mental health benefits by fostering feelings of social connectedness, alleviating feelings of isolation and cultivating a sense of belonging^21^. Yet, reaping these benefits necessitates an environment that is conducive to these outcomes, as positive social dynamics do not always naturally emerge within group settings^15^.

Burke and colleagues^22^ were the first researchers to compare the effects of individual and group approaches (*k* = 44) for PA in a meta-analysis focusing on adult populations. Outcomes included adherence, social interaction, quality of life, physiological effectiveness (for example, VO_2_max and body mass index) and functional effectiveness (for example, flexibility and strength). They examined four different types of PA context, two of which were individual (with and without contact/support from researchers or health professionals) and the other two were group-based (‘true’ groups and standard exercise classes). True groups were exercise classes where leaders intentionally apply group dynamics principles (for example, establishing group goals, fostering team identity and using group feedback), to enhance cohesion and engagement. By contrast, non-true groups involve participants exercising together without these structured group-building strategies^23,24^.

Burke et al. reported a trend across different outcomes suggesting that exercising in a true group was superior to exercising in a standard exercise class, which, in turn, did not differ from exercising at home with contact (from researchers or healthcare professionals). Furthermore, the latter condition was superior to exercising at home without contact^22^. However, the number of studies that included true groups was relatively small (*k* = 9), and these studies only examined adherence-related outcomes.

An update and extension of the Burke et al.^22^ meta-analysis is timely for a number of reasons. First, their review was published nearly 20 years ago, and 58 relevant new studies have since been published and included in this meta-analysis. Second, Burke et al. examined only face-to-face groups, as these were the formats generally available at the time. However, with technological developments and the introduction of online-delivered interventions^25^, an updated understanding of the literature requires expanding the definition of a PA group to include online groups^26^, and newer technologies such as virtual reality platforms^27^. These modalities were not widely accessible when Burke et al. conducted their review, potentially limiting the generalizability and scalability of their findings.

This shift is particularly relevant considering the post-COVID increase in remote and home-based PA formats, which offer enhanced accessibility, especially for underserved or rural populations^25^. Moreover, digital interventions may introduce different motivational dynamics (for example, altered social presence, autonomy or feedback structures) that can influence adherence and outcomes in ways that differ from traditional in-person settings^28,29^. These contextual changes highlight the need to reassess the relative effectiveness of group-based (online and in-person) versus individual interventions, and to update public health messaging to reflect this evolving delivery landscape.

Finally, since 2006, the use of device-based PA measures (for example, actigraphs) has enabled more sophisticated assessments of PA^30^. Differing from the Burke et al. meta-analysis, we excluded dyadic interventions, as their unique interpersonal dynamics differ from those in group or individual settings and could introduce conceptual heterogeneity^31,32^. We also included only studies directly comparing group-based and individual PA conditions, to specifically address the question about their relative effectiveness.

In sum, our study differed in several aspects from the Burke et al. meta-analysis^22^ in terms of search strategy and eligibility criteria (and analytic methods; see the next section). Our overall aim was to provide an up-to-date quantitative synthesis of existing literature comparing the behavioural, functional, psychosocial and physical health benefits associated with individual versus group-based PA in adults. Specifically, we examined whether group-based interventions, delivered either in-person or online, differ in effectiveness from individual-based interventions.

To guide our analysis, we addressed the following sub-questions:

Do online group-based interventions perform comparably to in-person groups?

Does intervention mode (online versus in-person) moderate the relationship between group format and behavioural, functional, psychosocial and physical outcomes?

## Results

### Search overview

**Fig. 1:**
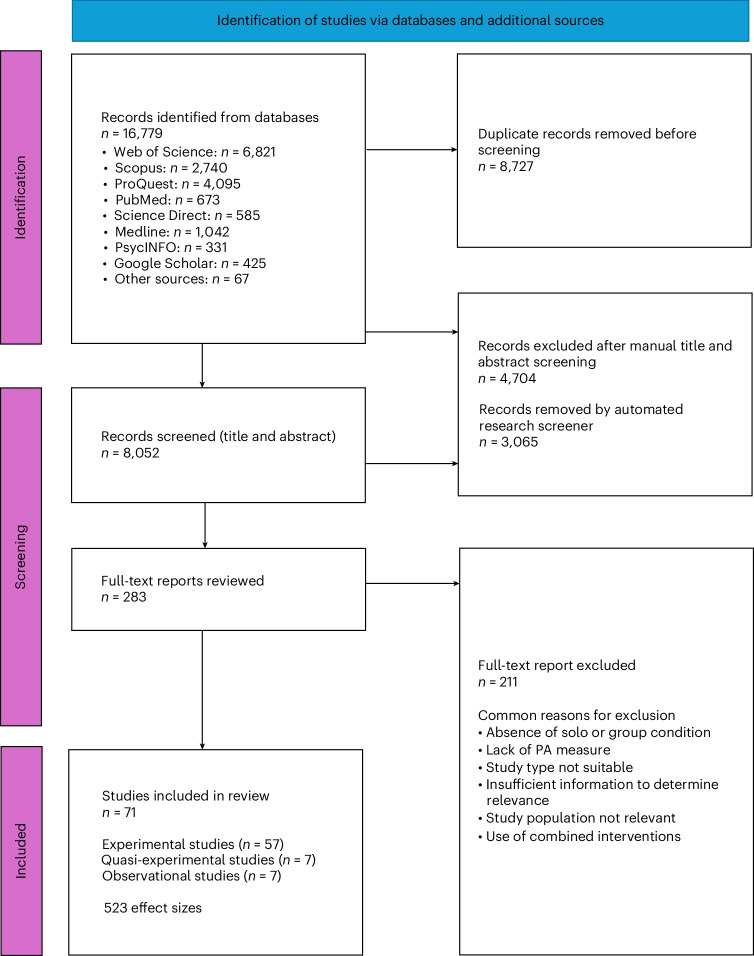
PRISMA flow diagram for study selection. Flow diagram showing identification, screening, eligibility assessment and inclusion of studies in the meta-analysis, in accordance with PRISMA guidelines.

Of 16,779 records identified, 71 studies (*k* = 523 effects) were included in the meta-analysis across PA (*k* = 125), functional (*k* = 157), psychosocial (*k* = 104) and health outcomes (*k* = 137) (Fig. 1). Power curves indicated that the minimal detectable effect size at 80% power, given the number of extracted effects and assuming high heterogeneity, was *g* = 0.058 for PA outcomes, *g* = 0.052 for functional outcomes, *g* = 0.062 for psychosocial outcomes and *g* = 0.050 for health outcomes^33,34^.

### Study characteristics

The 71 included studies (57 experimental, 7 quasi-experimental and 7 observational) contributed a total of *k* = 523 effect sizes and were conducted across 18 countries. Most were from Europe (37%, *n* = 26), followed by the USA (23%, *n* = 16), Australia (20%, *n* = 14), Canada (11%, *n* = 8) and Asia (9.8%, *n* = 7).

#### Participant characteristics

Over half of the studies (55%, *n* = 39) specifically targeted participants aged 50 years and older. Only 3% (*n* = 2) of the studies exclusively targeted male participants, while 10% (*n* = 7) focused solely on female participants. Most of the studies (59%, *n* = 42) involved healthy adult populations, while 36.6% (*n* = 26) focused on adults with clinical conditions. These clinical conditions were diverse, encompassing neurological conditions (*n* = 6), cancer (*n* = 4), cardiovascular conditions (*n* = 4), diabetes (*n* = 3), musculoskeletal and joint disorders (*n* = 6), respiratory (*n* = 3) and other conditions (for example, obesity, post-operative and mixed conditions; *n* = 2).

#### Intervention characteristics and delivery

Intervention duration ranged from 4 to 112 weeks (most lasting 8–16 weeks). Of the studies analysed, 72% (*n* = 51) utilized aerobic activities, either alone or combined with strength, balance training and/or flexibility training, whereas 20% (*n* = 14) focused solely on strength, flexibility and/or balance training. Overall, studies were primarily conducted in community settings (66%, *n* = 47), and groups were led by health professionals (for example, physical therapists or sport scientists; 44%, *n* = 31) or exercise instructors (38%, *n* = 27).

#### Characteristics of group-based conditions

Face-to-face was the predominant delivery method of group conditions (79%, *n* = 56), while online delivery methods represented a smaller but significant portion of the studies (20%, *n* = 14). Only 14 studies (20%) used ‘true groups’ in which leaders actively promoted peer interaction, whereas 48 (68%) used standard classes without such facilitation. Eight studies (11%) utilized group-based designs where participants did not meet at a scheduled time but instead engaged through an interactive online community, and one study (1.4%) used a cross-sectional design examining natural group settings. Of the 65 studies describing group meetings at set times, the majority (72%, *n* = 51/65) indicated that meetings occurred 2–3 times per week. Two studies (3%) reported meetings occurring less than once per week. A minority of studies reported peer leaders (*n* = 4) or research assistants (*n* = 3) as group leaders. Group sizes in the studies that reported them (70%, *n* = 50) ranged from 3 to 30 participants per exercise group.

#### Characteristics of individual conditions

Most individual conditions (72%, *n* = 51) were home-based, supported by phone (35%, *n* = 25) or SMS/email (23%, *n* = 16). Contact with the facilitator varied widely, ranging from no further contact beyond the initial session (*n* = 20, 28%) to 1–3 times per week (*n* = 11, 15%). The remaining studies did not specify the number of contacts or reported contact frequencies of less than once per week. More details on study characteristics are provided in Supplementary Table 1.

### Comparison of group-based and individual conditions on PA

**Fig. 2:**
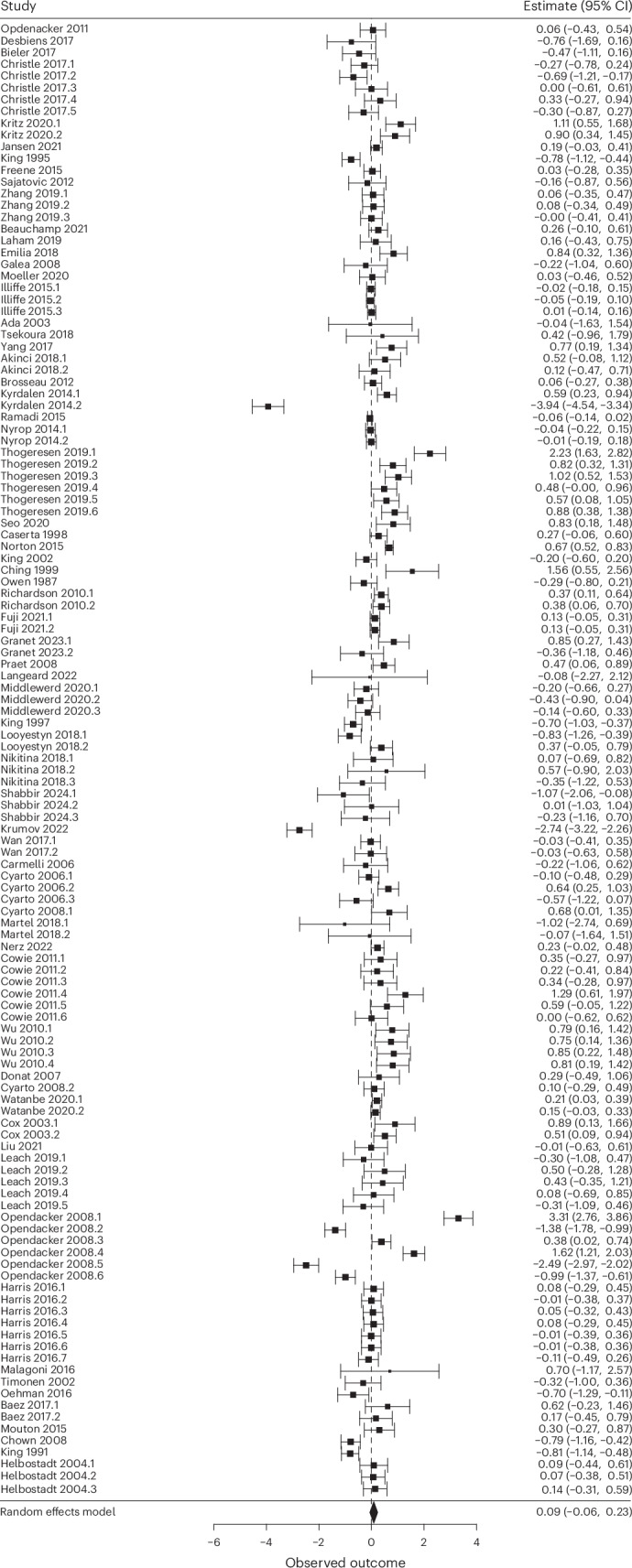
Forest plot of PA for combined effect sizes derived from proportions and standardized mean differences. Forest plot of effect sizes (Cohen’s *d*) derived from two-sided meta-regression analyses comparing PA outcomes between group-based and individual interventions. Squares represent study-specific effect size estimates, with square size proportional to study weight. Horizontal lines indicate 95% confidence intervals. The diamond represents the pooled effect size estimate with corresponding 95% confidence interval.

The difference in effect sizes for PA outcomes favoured group programmes, combined (positive signed effect), but the effect was small and did not reach statistical significance (*n* = 22,042, *k* = 125, *g* = 0.086, SE = 0.074, *P* = 0.249, 95% CI (−0.061, 0.233); Fig. 2). There was substantial heterogeneity (*I*^2^ = 90.738) between studies, which we examined through moderator analysis (‘Sensitivity analyses’). Likelihood ratio tests (LRTs) indicated significant variance in effects within studies (level 2; *σ*^2^ = 0.537, LRT = 418.378, *P* < 0.001) but not between studies (level 3; *σ*^2^ = 0.021, LRT = 0.186, *P* = 0.666), suggesting that heterogeneity may be due, in part, to inconsistencies between the various effects reported in individual studies. Prediction intervals (95% PI (−1.399, 1.571)) suggested an almost equal likelihood of effect sizes in similar future interventions favouring either group-based or individual conditions.

We also examined differences in PA outcomes between group-based and individual conditions in the subset of effect sizes derived from experimental and quasi-experimental studies only (and adjusting for pre-intervention levels of PA). Effect sizes were larger but remained non-significant (*n* = 16,886, *k* = 55, *g* = 0.141, SE = 0.134, *P* = 0.294, 95% CI (−0.126, 0.410)).

### Sensitivity analyses

#### Controlling for significant moderators

We examined the effect of the following moderators on overall effect sizes: group type, leader type, sample clinical status, age, gender, number of sessions per week, method of intervention delivery, intervention duration and study quality (Table 1). Group leader was a significant moderator, with studies involving trained group exercise leaders/instructors showing larger effects in favour of group conditions than those led by health professionals. However, including this moderator did not significantly improve model fit (omnibus test; LRT = 7.396, *P* = 0.060), suggesting that leader type was not sufficient to account for differences in PA outcomes across conditions. The variance explained by this moderator, partitioned across levels of the model, was (level 2) pseudo-*R*^2^ = 0.022 and (level 3) pseudo-*R*^2^ = 0.346.

**Table 1:** Effects of moderators on PA outcomes

	*g*	SE	*P* value	CI lower	CI upper
Moderator: group type					
Intercept (standard face-to-face group)	0.059	0.098	0.549	−0.135	0.252
Not specified	0.071	0.564	0.900	−1.044	1.187
Online/tele group	0.060	0.180	0.741	−0.297	0.417
True face-to-face group	0.063	0.236	0.789	−0.404	0.531
Moderator: group leader					
Intercept (not specified)	−0.077	0.104	0.460	−0.284	0.129
No group leader/other	0.156	0.194	0.424	−0.229	0.540
Researcher	0.210	0.290	0.470	−0.364	0.784
Trained group exercise leader/instructor	0.478	0.178	0.008	0.126	0.829
Moderator: health status					
Intercept (healthy)	0.165	0.089	0.065	−0.010	0.340
Intervention targeted at specific health condition	−0.255	0.160	0.113	−0.571	0.061
Moderator: age					
Intercept (continuous)	−0.072	0.442	0.872	−0.952	0.809
Age	0.002	0.003	0.780	−0.011	0.015
Moderator: gender					
Intercept (continuous)	0.099	0.093	0.289	−0.086	0.284
Female (%)	−0.004	0.007	0.517	−0.017	0.009
Moderator: number of sessions					
Intercept (3+ times per week)	0.038	0.125	0.765	−0.210	0.285
1–2 times per week	0.094	0.175	0.592	−0.252	0.440
1× per week or less	−0.089	0.309	0.774	−0.700	0.522
2–3 times per week	0.247	0.618	0.691	−0.978	1.471
Not specified/other	0.080	0.234	0.732	−0.382	0.543
Moderator: intervention duration					
Intercept (continuous)	0.149	0.120	0.218	−0.089	0.386
Number of weeks	−0.004	0.005	0.482	−0.014	0.007
Moderator: study quality					
Intercept (continuous)	−0.210	0.557	0.707	−1.311	0.892
Study quality	0.378	0.703	0.592	−1.013	1.769

Two-sided meta-regression results reporting effect sizes (Cohen’s *d*) for moderators comparing group-based and individual interventions; exact *P* values and 95% confidence intervals are shown.

Text in brackets describes the reference category for each intercept; SE = standard error; CI = 95% confidence interval.

#### Outliers

We identified six outlying effects based on standardized residuals (±3 SD from the model estimate) or Cook’s distances larger than three times the mean. These outliers contributed to heavy-tailed (that is, non-normal) effect size distributions, which can influence meta-analytic results (Liu et al., 2023). Aligning with our protocol, we conducted a sensitivity analysis to examine the impact of these outlying studies. After removing these cases, the pooled effect size slightly favoured group-based over individual conditions but remained small and non-significant (*n* = 21,122, *k* = 119, *g* = 0.094, SE = 0.059, *P* = 0.113, 95% CI (−0.023, 0.211)). We provide diagnostic plots (Supplementary Figs. 4–11) and a side-by-side comparison of models (Supplementary Tables 2–5) with and without outliers in the supplementary file.

#### Publication bias

**Fig. 3:**
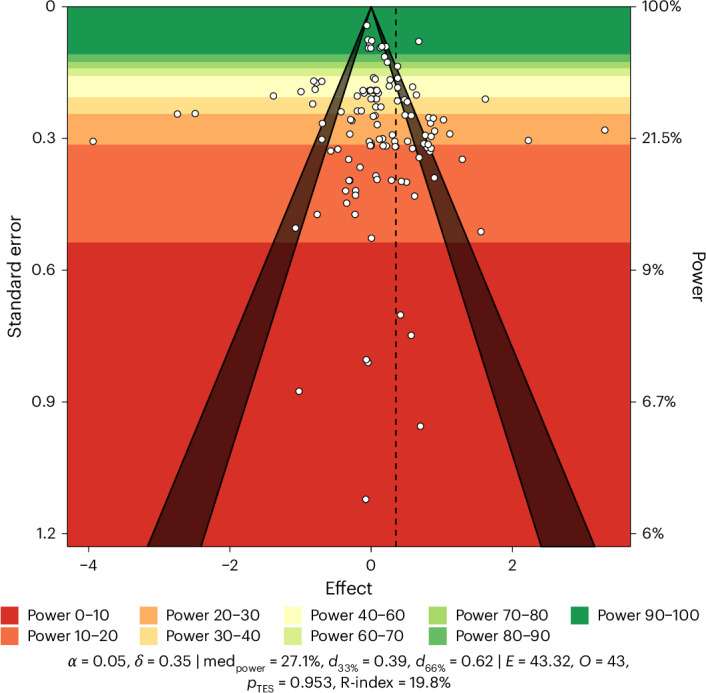
Sunset-enhanced funnel plot of included studies for PA outcome. Sunset-enhanced funnel plot showing study-level effect sizes (Cohen’s *d*) and precision for PA outcomes. Shaded regions indicate statistical significance based on two-sided tests. Meta-regression and associated inferential statistics are reported with exact *P* values and 95% confidence intervals in Tables 1–4.

A sunset-enhanced funnel plot was used to assess publication bias (Fig. 3). The plot appeared largely symmetrical, with effect sizes from studies with larger standard errors showing only slightly more dispersion than those from studies with more precise estimates of effect size. Egger et al.^35^ test of funnel plot asymmetry was conducted and indicated a small risk of publication bias (Intercept B0 = −0.035, *P* = 0.745, 95% CI (−0.177, 0.247)). However, only few studies had >80% power to detect small to moderate effect sizes (*g* = 0.35). In addition, a *z*-curve analysis was used to estimate the percentage of significant results expected if there was no selection bias (expected discovery rate (EDR) = 48.4%), as well as the estimated percentage of significant studies that would replicate if repeated perfectly (expected replication rate (ERR) = 68.8%)^36^. This analysis suggests that studies were approximately as likely to produce significant results as non-significant results, with a moderate level of replicability.

#### Testing equivalence between group and individual PA conditions

The absence of a statistically significant difference between group-based and individual conditions for PA outcomes does not imply support for the null hypothesis. Hence, we used the two one-sided test (TOST) procedure for equivalence testing as a follow-up procedure to formally test for the absence of a meaningful difference between condition types^37^. We used the model that combined mean differences and proportional effects and removed outlying effect sizes for this analysis, as outlying studies caused a heavy-tailed distribution, which violated the assumption of normality for the test. The equivalence test was significant (*z* = −1.667, *P* = 0.048), indicating that group-based and individual conditions produce statistically equivalent changes in PA.

### Comparison of group-based and individual conditions on functional, psychosocial and health outcomes

Pooled effect sizes for differences in functional outcomes were moderate and favoured group-based conditions, but were not statistically significant (*n* = 25,773, *k* = 157, *g* = 0.066, SE = 0.142, *P* = 0.065, 95% CI (−0.215, 0.346)). There were small but non-significant differences favouring group programmes for psychosocial outcomes (*n* = 18,223, *k* = 104, *g* = 0.292, SE = 0.233, *P* = 0.214, 95% CI (−0.171, 0.755)) and health-related outcomes (*n* = 31,607, *k* = 137, *g* = 0.125, SE = 0.075*, P* = 0.096, 95% CI (−0.023, 0.272)); see the Open Science Framework (OSF) page for forest plots^38^. There was moderate to substantial heterogeneity between studies for functional and health outcomes (*I*^2^_func_ = 74.480; *I*^2^_health_ = 43.384), and moderate to low heterogeneity for psychosocial outcomes (*I*^2^_psych_ = 17.268). Prediction intervals and variance partitioned across levels of the model are presented in Supplementary Table 6. Likelihood ratio tests indicated significant variance in effects within studies (level 2) and between studies (level 3), suggesting that the partitioning of variance across levels is meaningful.

A re-analysis of the data in which we examined only experimental and quasi-experimental studies and adjusted for baseline differences between groups did not reveal any substantive changes to our results (that is, effect size direction and statistical significance remained the same), with the exception of functional outcomes, for which a small, significant effect favouring group-based interventions emerged (*n* = 19,707, *k* = 140, *g* = 0.175, SE = 0.086, *P* = 0.044, 95% CI (0.005, 0.345)). Detailed results of these analyses are available in the project’s OSF repository^38^.

### Sensitivity analyses

#### Controlling for significant moderators

We examined the same moderators for our secondary outcomes as for the primary PA outcome (Tables 2–4). Group type, group leader and session frequency significantly moderated functional and psychosocial outcomes, whereas only group type significantly influenced health outcomes. For functional and psychosocial outcomes, studies that did not specify group type, group leader or session frequency tended to show stronger effect sizes compared with the respective reference categories (standard face-to-face groups, health professional-led interventions or ≥3 sessions per week). For health outcomes, studies using true face-to-face groups produced stronger effects favouring group over individual formats.

**Table 2:** Effects of moderators on functional outcomes

	*g*	SE	*P* value	CI lower	CI upper
Moderator: group type					
Intercept (standard face-to-face)	0.195	0.097	0.046	0.003	0.386
Not specified	−4.381	0.612	<0.001	−5.590	−3.173
Online/tele group	−0.174	0.285	0.541	−0.737	0.388
True face-to-face group	0.103	0.288	0.722	−0.466	0.671
Moderator: group leader					
Intercept (health professional)	0.182	0.100	0.070	−0.015	0.380
No group leader/other	−3.265	0.562	<0.0001	−4.374	−2.155
Researcher	−0.056	0.478	0.907	−1.001	0.889
Trained group exercise leader/instructor	0.070	0.275	0.801	−0.474	0.613
Moderator: health status					
Intercept (healthy)	−0.196	0.208	0.347	−0.606	0.214
Intervention targeted at specific health condition	0.566	0.305	0.065	−0.035	1.168
Moderator: age					
Intercept	0.010	1.285	0.981	−2.577	2.515
Age (continuous)	−0.001	0.019	0.997	−0.037	0.037
Moderator: gender					
Intercept	−0.273	0.411	0.508	−1.086	0.540
Female (%) (continuous)	0.581	0.599	0.333	−0.603	1.765
Moderator: number of sessions					
Intercept (3+ times per week)	0.081	0.1625	0.619	−0.240	0.402
1–2 times per week	0.1405	0.2014	0.486	−0.257	0.538
1× per week or less	0.6316	0.4431	0.156	−0.244	1.507
2–3 times per week	−0.0951	0.4235	0.823	−0.932	0.742
Not specified/other	−3.1631	0.5738	<0.0001	−4.297	−2.030
Moderator: intervention duration					
Intercept	0.184	0.133	0.170	−0.079	0.447
Weeks (continuous)	0.000	0.006	0.977	−0.012	0.012
Moderator: study quality					
Intercept (continuous)	0.068	0.145	0.641	−0.218	0.354
Study quality	−0.737	1.824	0.687	−4.340	2.866

Two-sided meta-regression results reporting effect sizes (Cohen’s *d*) for moderators comparing group-based and individual interventions; exact *P* values and 95% confidence intervals are shown.

Text in brackets describes the reference category for each intercept; SE = standard error; CI = 95% confidence interval.

**Table 3:** Effects of moderators on psychosocial outcomes

	*g*	SE	*P* value	CI lower	CI upper
Moderator: group type					
Intercept (standard face-to-face)	0.077	0.103	0.458	−0.128	0.282
Not specified	5.677	0.384	<0.001	4.916	6.439
Online/tele group	0.081	0.235	0.733	−0.386	0.547
True face-to-face group	−0.221	0.279	0.430	−0.774	0.332
Moderator: group leader					
Intercept (health professional)	0.067	0.292	0.818	−0.512	0.647
No group leader/other	1.737	0.640	0.008	0.468	3.006
Researcher	0.164	0.826	0.843	−1.474	1.803
Trained group exercise leader/instructor	−0.112	0.513	0.827	−1.131	0.906
Moderator: health status					
Intercept (healthy)	0.369	0.299	0.219	−0.223	0.961
Intervention targeted at specific health condition	−0.207	0.488	0.672	−1.176	0.761
Moderator: age					
Intercept	−0.087	1.375	0.950	−2.822	2.648
Age (continuous)	0.006	0.020	0.771	−0.034	0.046
Moderator: gender					
Intercept	0.291	0.247	0.241	−0.199	0.781
Female (%) (continuous)	0.004	0.008	0.578	−0.011	0.020
Moderator: number of sessions					
Intercept (3+ times per week)	0.041	0.318	0.898	−0.590	0.672
1–2 times per week	0.092	0.422	0.828	−0.744	0.929
1× per week or less	−0.517	0.766	0.501	−2.036	1.002
Not specified/other	2.489	0.677	<0.001	1.147	3.832
Moderator: intervention duration					
Intercept	0.276	0.163	0.094	−0.048	0.599
Weeks (continuous)	−0.013	0.008	0.133	−0.029	0.004
Moderator: study quality					
Intercept (continuous)	−1.291	2.587	0.619	−6.423	3.840
Study quality	1.950	3.173	0.540	−4.344	8.243

Two-sided meta-regression results reporting effect sizes (Cohen’s *d*) for moderators comparing group-based and individual interventions; exact *P* values and 95% confidence intervals are shown.

Text in brackets describes the reference category for each intercept; SE = standard error; CI = 95% confidence interval.

**Table 4:** Effects of moderators on health-related outcomes

	*g*	SE	*P* value	CI lower	CI upper
Moderator: group type					
Intercept (standard face-to-face)	0.071	0.082	0.388	−0.091	0.234
Online/tele group	−0.095	0.213	0.656	−0.516	0.326
True face-to-face group	0.457	0.203	0.026	0.055	0.859
Moderator: group leader					
Intercept (health professional)	0.067	0.097	0.490	−0.125	0.260
No group leader	0.242	0.306	0.430	−0.362	0.846
Researcher	0.144	0.594	0.809	−1.031	1.318
Trained group exercise leader/instructor	0.136	0.175	0.439	−0.210	0.482
Moderator: health status					
Intercept (healthy)	0.198	0.100	0.051	−0.001	0.396
Intervention targeted at specific health condition	−0.159	0.148	0.286	−0.451	0.134
Moderator: age					
Intercept	0.058	0.472	0.903	−0.883	0.999
Age (continuous)	0.002	0.007	0.800	−0.013	0.016
Moderator: gender					
Intercept	0.130	0.178	0.468	−0.223	0.482
Female (%) (continuous)	0.090	0.275	0.744	−0.455	0.635
Moderator: number of sessions					
Intercept (3+ times per week)	0.156	0.107	0.148	−0.056	0.368
1–2 times per week	−0.078	0.165	0.636	−0.403	0.247
2–3 times per week	−0.456	0.498	0.362	−1.441	0.530
Not specified/other	0.153	0.309	0.621	−0.458	0.765
Moderator: intervention duration					
Intercept	−0.021	0.117	0.860	−0.253	0.211
Weeks (continuous)	0.008	0.005	0.121	−0.002	0.018
Moderator: study quality					
Intercept (continuous)	1.285	0.681	0.061	−0.062	2.631
Study quality	−1.440	0.840	0.089	−3.101	0.222

Two-sided meta-regression results reporting effect sizes (Cohen’s *d*) for moderators comparing group-based and individual interventions; exact *P* values and 95% confidence intervals are shown.

Text in brackets describes the reference category for each intercept; SE = standard error; CI = 95% confidence interval.

To assess the influence of moderating factors on our results for each outcome, we constructed models that controlled for the combined effects of all significant moderators and compared them with models that did not contain moderator variables, using likelihood ratio tests. The omnibus test of the moderator model indicated a significant improvement over the baseline model for functional outcomes (LRT = 42.253, *P* < 0.001); the effect size adjusted for moderating variables was *g* = 0.039 (SE = 0.170, *P* = 0.819, 95% CI (−0.298, 0.376)). The omnibus test of moderators indicated a significant improvement over the no-moderator model for psychosocial outcomes (LRT = 65.862, *P* = <0.001); the adjusted effect size was *g* = 0.150 (SE = 0.161, *P* = 0.355, 95% CI (−0.170, 0.470)). The omnibus test of moderators did not indicate a substantial improvement over the no-moderator model for health-based outcomes (LRT = 5.837, *P* = 0.120). The proportion of variance explained by moderators for each secondary outcome at each level of the respective models (pseudo-*R*^2^) is provided in Supplementary Table 6.

#### Outliers

We identified outlying effect sizes for all secondary outcomes, which contributed to heavy-tailed (that is, non-normal) effect size distributions. Aligning with our protocol, we conducted sensitivity analyses to examine the impact of these effects. Regarding functional outcomes, six cases were identified as potentially influential using the same criteria as for PA outcomes. Removing these cases resulted in a pooled effect size, which was small and statistically significant, in favour of group-based conditions (*n* = 14,429, *k* = 151, *g* = 0.164, SE = 0.067, *P* = 0.015, 95% CI (0.032, 0.297)). Regarding psychosocial outcomes, 12 cases were identified as potentially influential. Removing these cases resulted in a small, non-significant effect size favouring group-based conditions (*n* = 14,429, *k* = 92, *g* = 0.221, SE = 0.142, *P* = 0.124, 95% CI (−0.062, 0.503)). Finally, regarding health-based outcomes, 12 cases were identified as outliers. After removing these cases, the pooled effect size was small but statistically non-significant, in favour of group-based conditions (*n* = 30,203, *k* = 125, *g* = 0.104, SE = 0.054, *P* = 0.055, 95% CI (−0.002, 0.211)). Side-by-side comparisons of models with and without outliers, as well as diagnostic plots, are provided in Supplementary Tables 2–5.

#### Publication bias

Visual inspection of the funnel plots for all secondary outcomes revealed some asymmetry, indicating potential publication bias (see Supplementary Figs. 1–3 for functional, psychosocial and health outcomes, respectively). The test of funnel plot asymmetry^35^ indicated a significant risk of publication bias for functional (Intercept B0 = −0.635, *P* = 0.010, 95% CI (−1.113, −0.157)) outcomes, but not for health-related (Intercept B0 = 0.115, *P* = 0.351, 95% CI (−0.128, 0.357)) or psychosocial (Intercept B0 = −0.228, *P* = 0.570, 95% CI (−0.568, 1.026)) outcomes. For all three secondary outcomes, few studies had >80% power to detect small to moderate effect sizes (*g* = 0.35). For all outcomes, *z*-curve analyses were used to estimate the ERR (functional = 76.0%; psychosocial = 75.2; health = 52.7%) and EDR (functional = 25.4%; psychosocial = 26.3%; health = 12.9%). These values indicated that studies may be likely to produce significant results but had potentially low replicability.

#### Testing equivalence between group and individual PA studies for psychosocial and health outcomes

When using the TOST procedure to test equivalence for secondary outcomes, we applied models with outliers removed to address violations of normality in the effect size distribution^37^. The equivalence test was non-significant for both psychosocial outcomes (*z* = 0.215, *P* = 0.585) and health-related outcomes (*z* = −1.596, *P* = 0.055), indicating insufficient precision in the data to either accept or reject the null hypothesis that group-based and individual conditions produce statistically similar differences in these outcomes. We did not conduct an equivalence test for functional outcomes because a significant difference between conditions emerged (after the removal of outliers), indicating that there was sufficient evidence to conclude a difference between group-based and individual conditions.

### Methodological quality and certainty of evidence

Quality ratings of included studies ranged from 0.36 to 0.95 with an overall mean of SQ_Overall_ = 0.78 (SD = 0.11). This moderate to high level of study quality suggests that, on aggregate, studies included in this meta-analysis were well conducted, appropriately analysed and clearly reported. When examining study quality for each individual outcome, average study quality was moderate to high for studies reporting PA outcomes (SQ_PA_ = 0.782, SD = 0.11), and high for studies reporting functional outcomes (SQ_Func_ = 0.82, 0.08), psychosocial outcomes (SQ_Psyc_ = 0.82, 0.07) and health outcomes (SQ_Health_ = 0.81, SD = 0.09). This did not change substantively when considering only experimental/quasi-experimental studies for any of the outcomes (SQ_PA_ = 0.78, SD = 0.11; RoB_Func_ = 0.82, SD = 0.08; RoB_Psyc_ = 0.81, SD = 0.07; RoB_Health_ = 0.82, SD = 0.07).

We assessed the certainty of evidence for randomized controlled trials (RCTs) and observational studies separately, following GRADE guidelines (Supplementary Table 1). For RCTs, the certainty was moderate for PA, functional and health outcomes, but low for psychosocial outcomes. Imprecision and risk of bias were common reasons for downgrading, with small sample sizes contributing to wide confidence intervals, making it difficult to determine the true effect of interventions. RCTs were downgraded owing to insufficient reporting of blinding and randomization, while non-experimental studies showed bias and inconsistency from lack of control for confounding factors and from using varied outcome measures. For PA, RCTs were rated moderate in terms of certainty of evidence, downgraded for inconsistency owing to varying activity measures. Non-experimental studies were rated low for often relying on adherence as a proxy for activity behaviour and for using self-reported measures. Functional and health outcomes were rated moderate in terms of certainty of evidence for both study designs, downgraded owing to risk of bias and imprecision. Psychosocial outcomes were rated low, driven by heterogeneous self-reported measures prone to bias and indirectness. Specifically, many studies measured general well-being instead of specific psychosocial or social outcomes such as mental health or loneliness.

## Discussion

The main aim of the present study was to conduct an up-to-date meta-analysis comparing group versus individual approaches for promoting PA, functional, health and psychosocial outcomes in adults. This review extends Burke et al.^22^ by incorporating studies with new technologies, delivery formats (for example, online and virtual) and more diverse outcome measures. Across 71 studies, we found statistically equivalent outcomes for PA and health, regardless of delivery format. Group-based formats showed a small, significant advantage for functional outcomes after outlier removal. Moderation analyses indicated slightly larger health effects for ‘true groups’.

Our findings—that group-based and individual approaches did not differ significantly, except for functional outcomes (where group approaches showed superiority after removing outliers)—align with those of Burke et al.^22^. The latter reported no differences between ‘standard’ exercise classes (in which group dynamics strategies were not used to enhance group cohesion) and home-based programmes with contact from researchers or health professionals.

Further, our moderation analysis on group type showed that different types of group resulted in broadly similar outcomes. The only exception was ‘true groups’ (that is, those using group dynamics principles). Consistent with Burke et al.’s finding that these groups outperform standard exercise groups, our moderator analysis showed that effect sizes for health outcomes (but not other outcomes) were slightly larger for true groups compared with standard face-to-face groups. However, the inclusion of group type as a moderator did not substantially influence the overall effect size, suggesting that any variation owing to different group types is not sufficient to account for differences in health-related outcomes between group-based and individual conditions. It should be noted, nevertheless, that there was a limited number of true group conditions (*k* = 14) and in these studies it was not possible to determine the number of group-based strategies used. This is unsurprising since previous systematic reviews of general PA promotion programmes^39^ and PA programmes for cancer survivors^40^ have shown that many ‘group based’ PA promotion programmes do not fully use a theoretical approach or provide replicable strategies that may enhance group influence or perceptions. Given the limited number of true group conditions, we were unable to examine differences between online and face-to-face true groups, although this would be an interesting avenue for future research.

The results of the TOST showed statistical equivalence between individual and group conditions for PA outcomes, but not for health or psychosocial outcomes. This implies that we can be reasonably confident that group-based and individual interventions have similar effects in terms of PA outcomes. However, it was not possible to conclude whether group or individual interventions are superior for health or psychosocial outcomes. This could be due to heterogeneity in how these outcomes were assessed, poorly described groups or different types of intervention. This finding underscores the need for consistent definitions and standardized measurement to improve comparability across studies.

The equivalence in PA outcomes has important practical implications in terms of intervention flexibility and scalability. Group interventions can foster social interaction and accountability, potentially have a greater reach and are scalable^39^. However, individual formats can offer greater accessibility, autonomy and personalization. Our findings suggest that both formats can be viable pathways to PA behaviour change. Offering choice-based or hybrid models, where individuals select the format that best fits their preferences, needs and life circumstances, may help expand the reach and inclusivity of PA programmes. For example, individuals with limited time, social anxiety or a preference for exercising alone could benefit from self-guided or online formats, particularly in rural or resource-limited settings.

From a theoretical perspective, these findings can be interpreted through the lens of self-determination theory, which highlights the importance of satisfying the psychological needs for autonomy, competence and relatedness for the facilitation of behaviour change maintenance^16,17^. The absence of clear differences in PA between group and individual formats may suggest that both formats are capable of supporting these needs, albeit through different mechanisms. Individual formats may promote autonomy and competence through flexible, self-paced engagement, while group settings may foster relatedness and competence via social support and structured guidance. It is possible that when these needs are sufficiently met, the delivery format becomes less critical to ensuing outcomes.

For functional outcomes (for example, balance, coordination and muscle strength), we did not find a substantive difference in our primary analysis; however, sensitivity analyses indicated that group-based approaches became significantly more effective after removing outliers, as well as when restricting the analysis to experimental/quasi-experimental studies (that is, excluding correlational studies). While results of sensitivity analyses should be interpreted with caution, they can be useful for hypothesis generation^41^. We suggest that in some cases (for example, when groups are formalized such as in experimental studies), group structure and support may better promote functional gains compared with individual contexts, as individuals in a group can benefit from observing others and receiving feedback.

Our findings can also be explained via the lens of social cognitive theory^18,19^, which emphasizes the role of observational learning, group modelling and vicarious reinforcement in enhancing self-efficacy and behaviour change. Within group-based exercise settings, individuals may feel more capable and motivated after observing peers succeed at a task, receiving real-time feedback from an instructor or perceiving shared progress within the group^18^. Previous research supports this, showing that the presence of others in an exercise class can facilitate skill mastery and confidence^42^. These mechanisms may be particularly salient for functional tasks requiring coordination or technique, which benefit from visual learning and real-time adjustment compared with more general PA outcomes, where intensity and consistency may matter more than group dynamics. Such functional advantages suggest that group-based approaches could be valuable for rehabilitation programmes targeting older adults and clinical populations, where preserving functional ability is critical^43^.

Another important new finding of our review was that online and standard face-to-face groups did not differ in their effectiveness. This finding suggests that the critical elements of group conditions, such as social support, accountability and shared goals, may be effectively facilitated in virtual settings. From the perspective of self-determination theory, relatedness may be fostered through virtual communities, live sessions or peer forums embedded in these platforms where users can share progress, exchange encouragement and engage in group challenges. Aligning with suggestions by other researchers^44^, it is possible that the quality of interaction may be more important than the mode of delivery in driving successful outcomes. Online formats could potentially support also autonomy via flexible scheduling and self-paced engagement. Competence could be supported through structured guidance and tailored feedback, for example, through virtual coaching platforms that provide personalized goals and progress tracking.

The similar effectiveness of online and face-to-face groups across psychosocial, functional and PA-related outcomes supports the potential of online interventions to reach populations who face barriers to in-person participation, such as mobility limitations, transportation challenges or geographic isolation. Online formats address some logistical demands (for example, transport and staffing), expand scalability and extend reach to underserved populations, thereby supporting both cost-effectiveness and equity. Building on these findings, policy initiatives could integrate virtual PA programmes into public health strategies, for example, by offering virtual and group-based rehabilitation programmes for older adults or embedding digital PA support into chronic disease prevention and management initiatives. Workplace wellness policies could also leverage virtual, group-based PA programmes to enhance reach and scalability, particularly for national PA promotion efforts in rural or resource-limited settings.

### Strengths, limitations and future research directions

Our meta-analysis had several strengths. Specifically, we used a three-level meta-analysis framework that enabled us to account for dependencies among effect sizes within studies, thereby providing a more nuanced analysis that captured variability at different levels, such as within studies, between studies and between subgroups. This added layer of precision allows for a richer understanding of the data and helped avoid biases that may arise from treating all effect sizes as independent. The integration of the TOST equivalence test further strengthens the analysis by allowing us not only to detect significant differences but also to assess practical equivalence between conditions. This is particularly valuable for understanding whether interventions are similarly effective, supporting more definitive and actionable conclusions that can be translated into informed, evidence-based decisions.

Study quality was assessed using the QualSyst tool and overall literature quality using GRADE. Average quality was relatively high, indicating that studies were generally well conducted, analysed and reported. However, GRADE ratings showed moderate certainty for all outcomes, except for psychosocial outcomes, which was low.

Although most studies were experimental, a small number were observational or cross-sectional, introducing potential limitations such as measurement imprecision and confounding. In line with our protocol, we aggregated both experimental and non-experimental studies^45^. Secondary analyses restricted to experimental and quasi-experimental studies yielded results consistent with the main analysis, suggesting that the inclusion of observational studies had minimal impact on overall findings. However, methodological concerns across the literature, including reliance on cross-sectional designs in some outcome domains, risk of bias, high heterogeneity, limited control for confounders, self-reported outcomes, use of indirect proxies (for example, class attendance as a proxy for PA) and small sample sizes, indicate scope for methodological improvement in future research.

Psychosocial outcomes in our review were particularly affected by imprecision, risks of bias (for example, lack of blinding) and heavy reliance on self-report measures, raising concerns about the robustness of the associated findings. While self-report remains a necessary method for assessing many psychological and social constructs, future research may benefit from prioritizing more specific and well-validated measures (for example, affect and loneliness) over broad or diffuse constructs (for example, general psychological well-being). In addition, meta-analytical comparisons of group-based versus individual interventions targeting clearly defined psychological and social outcomes could offer greater granularity regarding intervention effects and advance construct-specific conclusions.

We observed imprecision in both primary and secondary outcome effect size estimates, indicating uncertainty around the true size of differences between group and individual intervention programmes. This issue is potentially attributable to the substantial heterogeneity observed in all effect sizes, which underscores variability in study designs, populations and intervention implementation across studies. Differences in participant demographics, settings (for example, community versus clinical) and intervention characteristics (for example, frequency and duration) likely contributed to inconsistent results.

Although our sensitivity analyses indicated that the overall pattern of findings was largely robust to between-study differences, heterogeneity remains an important limitation. Inconsistent or incomplete reporting of intervention settings (for example, community, workplace and clinical) and intervention characteristics (for example, group size) prevented us from examining whether these factors moderated intervention effects. To improve interpretability and better isolate the conditions under which group or individual formats are most effective, future research should adopt clearer reporting standards for intervention context and consider standardizing key intervention characteristics or incorporating planned subgroup/moderator analyses. Such steps would enhance comparability across studies and improve the precision of effect size estimates.

Finally, our obtained effect sizes were not disattenuated for measurement error. We suggest that this is likely to have a minimal impact on results, since measurement error should affect both individual PA and group-based PA equally; however, we acknowledge that correcting for this would have provided a purer assessment of effects. In addition, we excluded grey/unpublished literature, including dissertations and conference proceedings, as these typically provide insufficient methodological detail or extractable data. While this decision aligns with our methodological rigour, it may bias results by overrepresenting published studies, which are more likely to report positive effects.

Future research could explore potential moderators to better understand which individuals respond best to group versus individual PA conditions. Factors such as personal preference, personality traits, physical and psychological capability, and social or physical opportunity may influence individual responses^46,47^. Interaction effects between moderators (for example, group type × leader role or duration × setting) may offer additional explanatory insight, but limited reporting in the primary studies precluded reliable testing. Larger datasets or access to the raw data from all primary studies may enable future analyses to systematically explore these interactions.

It may also be valuable to determine whether specific group formats (for example, synchronous versus asynchronous, instructor-led versus peer-supported) better align with individual characteristics or contextual demands. Hybrid models that combine group and individual elements (for example, periodic group sessions alongside personal exercise plans) could be tested for synergistic benefits^48^. Where integration is not feasible, formats should be tailored to individual preferences, intended outcomes (for example, functional, psychological and social) and contextual constraints (for example, personnel time, facility space, available social support and scalability)^49^.

## Methods

### Protocol and registration

This meta-analysis was pre-registered with PROSPERO (CRD42021271452) and adhered to the Preferred Reporting Items for Systematic Reviews and Meta-Analyses (PRISMA) guidelines^50^. We followed the protocol^51^ for objectives, eligibility and primary analyses, with a small number of refinements: the search period was extended, screening was restricted to English-language studies, GRADE ratings were added to supplement QualSyst, and equivalence testing (TOST) was incorporated. In addition, psychological and social outcomes were merged and reported as ‘psychosocial’, and physical health outcomes were separated into functional and health domains. The originally pre-registered outcome structure (separate psychological and social outcomes, and a single physical outcome domain) was not analysed, as substantial overlap in outcome measures and inconsistent reporting across primary studies rendered these distinctions methodologically uninformative and analytically unfeasible. Full details are provided in Supplementary Table 7.

### Eligibility criteria

To be eligible for this meta-analysis, the authors of the studies had to:

1. Report any type of PA or exercise condition or intervention, performed in at least one individual (home-based, online or face-to-face) and at least one group setting. Group settings could include face-to-face or online formats and needed to include at least three group members. Dyadic interventions were excluded as they have been meta-analysed recently^31^, and also for the reasons explained earlier. Studies that described multi-component programmes (for example, combining PA with nutrition counselling) were excluded unless they provided separate effect sizes for the PA component(s).
2. Report at least one measure of PA behaviour/adherence (primary outcome of interest), which could be based on self-reports or device-based measurements.
3. Include adults aged 18 and older, regardless of their health status. Studies that reported data for mixed age groups but included a clear breakdown for adults were also eligible.

We included quantitative studies with experimental, quasi-experimental and correlational designs (longitudinal or cross-sectional). Both intervention and observational studies were eligible if they reported sufficient information to compute effect sizes. Studies were excluded if they focused on competitive sports, if they lacked sufficient data to compute an effect size (and the authors could not provide this data upon request), or if they were unavailable in English. Conference proceedings and theses/dissertations were excluded owing to limited methodological detail and incomplete data reporting. Only one relevant abstract was identified, which lacked usable data.

### Information sources

Following best practices for systematic reviews and meta-analyses, we conducted a search of multiple databases via platforms such as Web of Science and ProQuest, ensuring broad coverage and methodological rigour^52^. No date restrictions were applied, and all searches were conducted in English. We included studies from inception given that we used somewhat different criteria from the 2006 meta-analysis^22^. In addition, manual searches of reference lists of included studies were conducted, and forward and backward citation tracking was used. Google Scholar was used to identify any additional relevant literature. The final search was completed on 19 March 2024.

### Search strategy

The search strategy was designed by the first author in collaboration with an experienced research librarian and reviewed by the research team. Literature searches were conducted in Web of Science and ProQuest from database inception to 19 March 2024. Search terms combined keywords and subject headings related to PA (for example, PA and exercise), intervention format (for example, group, collective, group-based, individual, solo, home-based and online) and outcomes (for example, adherence, fitness, functional, health, psychological and social). Reference lists of included studies were manually searched, and forward and backward citation tracking was conducted using Google Scholar. Full search strings for all databases are provided in the supplementary file (Part 1: Search strategy)

### Study selection

All identified records were imported into Endnote v.21 to remove duplicates. The remaining studies (*n* = 8,052) were screened in 2 stages.

#### Title and abstract screening

Two independent reviewers (M.K. and D.O.) screened the titles and abstracts of 62% of the retrieved studies (4,987/8,052) based on eligibility criteria. The Research Screener software^53,54^, a validated machine learning tool^53^ trained on eligible studies, was used to assist with the initial screening process by automating the exclusion of irrelevant studies. Using this tool, in the second step, the reviewers screened 51% of the imported studies (3,152/6,217), manually reviewing those flagged as potentially eligible. This percentage far exceeded the 5%–35% recommended by the authors of the tool^53^, as well as generic recommendations of a minimum of 20% for automated screening tools^54^. All AI-flagged potentially eligible abstracts were manually reviewed, and final inclusion decisions were verified by the two independent human reviewers (M.K. and D.O.). The tool excluded 3,065 studies as irrelevant. In an updated search, an additional 1,835 studies were manually screened by the reviewers using Endnote. Any conflicts between reviewers (*n* = 212) were resolved through discussion or with input from a senior researcher (C.T.-N.).

#### Full-text screening

Full texts of the studies deemed eligible after the title and abstract screening (*n* = 283) were retrieved and assessed for eligibility by at least 2 independent reviewers (M.K. and D.O.). Disagreements were resolved either through discussion (*n* = 55) or consultation with a third reviewer (H.R., C.T.-N. or N.N.; *n* = 16). Agreement was reached in 213 cases (75%), and interrater reliability was moderate (Cohen’s *κ* = 0.47). Reasons for exclusion at this stage are documented in Fig. 1.

#### Data extraction process

A standardized Excel form was used for data extraction, which is available on the OSF^38^. Three independent reviewers (M.K., H.R. and D.O.) extracted study and participant details (for example, author, design, sample size, age, gender and health status), PA intervention characteristics (for example, type, duration, intensity and frequency), setting (for example, community and healthcare), delivery method (in-person versus online), facilitator contact, group and individual format characteristics, and reported outcomes. Extending the work of Burke et al.^22^, groups were categorized into true face-to-face (*n* = 11), standard face-to-face (*n* = 45), true online (*n* = 3), standard online (*n* = 3), online community with interactive components (*n* = 8) and other (*n* = 1). All studies were screened by at least two reviewers, with disagreements resolved by consensus or consultation with a senior researcher (C.T.-N. or N.N.). We contacted the authors of five studies with missing or unclear data, sending two email reminders spaced 2 weeks apart. Two authors did not respond, and their studies were excluded owing to insufficient information.

#### Outcomes and grouping

Primary outcomes for our review included self-reported (for example, frequency, duration and type of PA) or device-based measures of PA (for example, step counts and accelerometer data). Secondary outcomes were grouped into three distinct categories: (1) psychosocial outcomes, including measures of psychological well-being (for example, life satisfaction and mental health) and social interaction (for example, social support and group cohesion); (2) health outcomes, referring to physical indicators such as VO_2_max, body mass index and blood pressure; and (3) functional outcomes, such as strength, balance and mobility. Details of these groupings and the specific variables included in each category are provided on the project’s OSF page^38^.

#### Moderators

We pre-specified and a priori registered the following moderators for later meta-regression: group type, leader type, clinical status, age, gender, session frequency, delivery format, duration and study quality.

### Quality and risk of bias of individual studies

#### Methodological and reporting quality

The quality of the included studies was assessed by three independent reviewers (M.K., H.R. and C.T.-N.) using the 14-item Standard Quality Assessment Criteria for Primary Research Papers (QualSyst^55^). QualSyst provides a standardized checklist and manual specifically for evaluating the methodological rigour and reporting quality of a wide range of study designs based on items tapping study size, outcome appropriateness, control of confounding factors and clarity in reporting of results. Scores for this tool range between zero and one with a higher score indicating higher study quality.

#### Certainty of evidence and risk of bias

The risk of bias of the whole body of literature was independently assessed by two reviewers (M.K. and H.R.) using the GRADE tool^56^, which evaluates factors such as study limitations, consistency of results, imprecision, indirectness and publication bias. The final grading was reviewed by the other team members. Certainty of evidence was evaluated separately for experimental and observational studies (Supplementary Table 1), and independently for PA, functional, psychosocial and health outcomes. The evidence was downgraded, where needed, based on factors such as serious study limitations, inconsistency, indirectness, imprecision and a high risk of publication bias.

### Data analyses

#### Calculation of effect sizes

We calculated standardized mean differences to have a comparable index of effect size for outcomes (for example, PA) measured using different metrics, scales or tools. We used Hedges’ *g* as the effect size metric to account for the relative size of each sample^57^. Effect sizes were calculated from means, standard deviations and sample sizes of experimental or intervention groups at post-intervention, using existing formulas^58^. The values of 0.2, 0.5 and 0.8 or greater were deemed as small, medium and large, respectively^59^. In addition, many of the included studies reported proportional metrics of PA adherence and some non-experimental studies reported correlational effect sizes. We calculated effect sizes for these proportional measures as odds ratios, which were converted to *g* (ref. ^58^), an approach that was also used for correlational effect sizes. We combined converted effects with effect sizes derived from mean differences. Our primary analysis used all available effect sizes (for example, from observational/cross-sectional and intervention studies) and combined effect sizes derived from mean differences and proportional data. Data analysis scripts, extracted datasets and additional methodological materials are available via the OSF^38^.

#### Statistical synthesis of effect sizes

The inclusion of multiple effect sizes from a single study, as was the case in our review, violates the assumption of independence in effect sizes in traditional meta-analyses. Consequently, we used a 3-level random effects model to decompose sampling variance of the observed effect sizes (level 1), and variance within studies (level 2) and between studies (level 3), thus accounting for interdependences in effect sizes from the same study^60^. We conducted all statistical analyses metafor, metaSEM and metaviz packages^61^ in R (version 4.3.1)^62^, following guidelines^63^. In addition, because non-significant effects obtained from the random effects model cannot be used to infer evidence for the null hypothesis (that is, non-significant effects do not necessarily infer an absence of a difference), we used a TOST procedure for equivalence testing^37^ to formally test for the absence of a meaningful difference between individual and group conditions, when the main effect sizes were non-significant. To provide a brief primer on the interpretation of this procedure, a significant TOST test indicates that the two conditions produce statistically equivalent effect sizes, and we can thus accept the null hypothesis that there is no difference between the two. A non-significant TOST test indicates that there is insufficient precision to conclude either for or against the null hypothesis. We specified equivalence bounds (the upper and lower limits of effect sizes that we would consider meaningful) at *d* = ±0.19, corresponding to the 25th percentile of effect sizes 6 months post PA intervention, based on a relevant meta-analysis^64^. We used the metaviz^65^ that produces ‘sunset’ funnel plots to show information on the statistical power of each individual study included in the synthesis.

#### Sensitivity analyses

In addition to our primary analyses, we conducted several sensitivity analyses for primary and secondary outcomes to assess the robustness of findings and examine potential sources of heterogeneity^63^. First, we assessed the influence of outlying effect sizes on the model. Outliers were defined as effects with standardized residuals exceeding three standard deviations from the model estimate or Cook’s distances larger than three times the mean. We conducted these analyses to assess the impact of statistically extreme effect sizes that may disproportionately influence pooled estimates or produced non-normal effect size distributions. Second, we examined effects of potential moderators by including the following variables as covariates in a meta-regression: group type (the style of group setting used, such as online or standard face-to-face groups), group leader (the individual leading the group), health status (whether participants were healthy or had a specific medical condition), age (average age of the sample), gender (the proportion of female identifying participants in the sample), sessions per week (the frequency that groups or individuals exercised at), intervention duration (the number of weeks that the intervention ran for) and study quality (the study’s methodological and reporting quality rating). This moderator analysis allowed us to compare effect sizes across different moderator categories/quantities.

We made an a priori decision to include both experimental and observational studies to ensure comprehensive coverage of the literature and to reflect the full range of evidence on PA interventions across real-world contexts. While experimental designs typically provide greater evidentiary strength, observational studies can enhance ecological validity^45^. To assess whether their inclusion biased results, we conducted a secondary analysis restricted to experimental and quasi-experimental studies, adjusted for pre-intervention PA levels. Results were consistent with the main analysis, indicating minimal impact on overall findings.

### Reporting summary

Further information on research design is available in the Nature Portfolio Reporting Summary linked to this article.

## Supplementary information

Supplementary Information Supplementary Methods and Analysis, Figures 1–11, Tables 1–7 and protocol adherence and diversions.

Reporting Summary

Peer Review File

Supplementary Tables 1

## Data Availability

The dataset generated and analysed during this meta-analysis and additional materials are publicly available via the Open Science Framework (OSF) at 10.17605/OSF.IO/XT2G4 (ref. ^38^).

## Code availability

All custom code used for data processing and statistical analyses in this meta-analysis is publicly available via the Open Science Framework (OSF) at 10.17605/OSF.IO/XT2G4 (ref. ^38^).
