# Supplementary material for: Individual versus group-based interventions: a systematic review and meta-analysis of physical activity, functional, psychosocial and health outcomes
Source: Nat Hum Behav. 2026 Apr 15;10(6):1109–21. doi: 10.1038/s41562-026-02429-0 (PMC13290480; doi:10.1038/s41562-026-02429-0)
Supplement: Supplementary file 1 — Supplementary Methods and Analysis, Figures 1–11, Tables 1–7 and protocol adherence and diversions. [file 41562_2026_2429_MOESM1_ESM.pdf]

nature human behaviour

Supplementary information https://doi.org/10.1038/s41562-026-02429-0

Individual versus group-based interventions: a systematic review and meta-analysis of physical activity, functional, psychosocial and health outcomes

In the format provided by the authors and unedited

Overview

This Supplementary File provides in the first part additional analyses, and diagnostic checks supporting the main findings of the meta-analysis. The second part provides documentation of adherence to the registered PROSPERO protocol. Supplementary figures, tables, and references are provided to ensure transparency and reproducibility.

Part 1: Supplementary Information on Methods and Analysis

This section provides detailed methodological information and additional analyses supporting the main manuscript. It includes the full search strategy, eligibility criteria, study selection and data extraction procedures, outcome domain definitions, statistical modeling approaches, risk-of-bias and certainty assessments, evaluations of evidential strength and small-study effects, robustness and sensitivity analyses, and variance component estimates.

Search strategy

The electronic search was conducted from the inception of the databases to 19th March 2024. An electronic search was performed from inception of the databases to 19th of March 2024. The searched databases included Web Science, Scopus, ProQuest, PubMed, ScienceDirect, Medline, PsycINFO, and Google Scholar, with additional manual searches carried out in relevant journals. We also performed backward and forward citation tracking to identify further studies that may not have appeared in the initial search results. Search terms were carefully selected to include a combination of free text terms and subject headings, where applicable, to maximize sensitivity and relevance. We sought peer-reviewed articles published in English, and searches were restricted to quantitative studies, including randomized controlled trials (RCTs) and clinical trials, focusing on adult populations, group-based versus individual interventions, physical activity outcomes, and psychosocial or physical health impacts.

With the assistance of an experienced librarian, we developed the search string to achieve a balance between broad coverage and high specificity, focusing on identifying studies that compare solo and group formats in physical activity interventions/exposures. We identified key terms that were also identified from previous systematic reviews and meta-analyses on

physical activity interventions, to provide additional specificity. Relevant databases, such as Mesh, were utilized to identify key terms and incorporate subject-specific terms into our search strategy. To reduce irrelevant results, broader terms were refined to focus on the context of physical activity. For example, "exercise" was paired with terms like "intervention" and "program" to exclude general or unrelated exercise studies, while "group" and "solo" were structured with adjacencies (e.g., "group adj3 exercise") to capture relevant comparisons without unrelated group-based activities.

The following terms were searched as free text and using Boolean operators (AND/OR/NOT) in listed databases.

Adult, Adults, "aged 18 and over", "aged 18 and older"
Group, "with others", "with peers", "group-exercise", "peer-support*"
Communal, "in-center", "in-centre", accomp* (accompanied)
"group-interaction*", "group-based", collab* (collaborative), collective
"group-mediated", "group-led", cohes* (cohesive), *class*, *social*, "face to face"
buddy, group*, group-dynamics*, mentor*, peer*, peer-support*, interaction*
team-based, team exercise, group fitness, "shared exercise", "collaborative exercise"

Alone, Home*, "personal training", Individual
"non-group", "non-social", Solo, "without support", "without others"
"solo exercise", "individual-based PA", "self-guided exercise", "alone vs with others"

center*, centre*, home, home-based, in-center, in-centre
"mall-walk*", "peer-led exercise", "group-led PA"

Intervention, Program, "exercise-program*", Programme, Trial, Exposure
"physical activity intervention", "head-to-head comparison", "controlled trial"
"effectiveness comparison", "group-based vs individual-based intervention"

Outcome, Benefit, Improve, Impact, Change, Effect, Differ
"Adherence", "Engagement", "Retention", "Health outcomes", "Social outcomes"
"Motivation", "Psychosocial outcomes", "Behavior change", "Well-being outcomes"

"Physical activ*", "physical move*", "physical training", "physical strength"
Gait, "physical performance", "physical fitness", *Exercise*

"mall-walk*", Swim, Walk, "resistance training", Biking, Jog, Jump, Hike, Dance, Run
"walking group", Yoga, Bike, aerobic*, cycling, exercise-class, exercise-group
running, resistance, training

Compar*, Versus, Vs, "alone vs group", "individual vs group exercise"

Qualitative, Review, "systematic review", "meta-analysis", "meta-synthesis"

Details on Specific Search Strategy for each Database

Web of Science:

TS=(adult* OR "aged 18 and over" OR "aged 18 and older" OR "adults")

AND

TS=(Group OR "with others" OR "with peers" OR "group-exercise" OR "peer-support*" OR communal OR "in-center" OR "in-centre" OR accomp* OR "group-interaction*" OR "group-based" OR collab* OR collective OR "group-mediated" OR "group-led" OR cohes* OR "class" OR social OR "face to face")

AND

TS=(Alone OR home* OR "personal training" OR individual OR "non-group" OR "non-social" OR solo OR "without support" OR "without others")

AND

TS=("physical activ*" OR "physical move*" OR "physical training" OR "physical strength" OR "quality of life" OR "gait" OR "physical performance" OR "physical fitness" OR exercise OR "mall-walk*" OR swim* OR walk* OR "resistance training" OR biking OR jog* OR jump* OR hik* OR danc* OR run* OR "walking group" OR yoga OR bike*)

AND

TS=(intervention OR program* OR trial OR "exercise-program*" OR exposure)

AND

TS=(Outcome OR benefit* OR improv* OR impact* OR change* OR effect* OR differ*)

AND

TS=(psychosocial OR physical OR psychological OR "mental health" OR social OR health OR wellbeing OR function* OR physic*)

AND

TS=(compar* OR versus OR vs)

NOT

TS=(qualitative OR review OR "systematic review" OR "meta-analysis" OR "meta-synthesis")

SCOPUS

(TITLE (adult* OR "aged 18 and over" OR "aged 18 and older" OR "adults") OR ABS (adult* OR "aged 18 and over" OR "aged 18 and older" OR "adults")) AND (TITLE (group OR "with others" OR "with peers" OR "group-exercise" OR "peer-support*" OR communal OR "in-center" OR "in-centre" OR accomp* OR "group-interaction*" OR "group-based" OR collab* OR collective OR "group-mediated" OR "group-led" OR cohes* OR "class" OR social OR "face to face") OR ABS (group OR "with others" OR "with peers" OR "group-exercise" OR "peer-support*" OR communal OR "in-center" OR "in-centre" OR accomp* OR "group-interaction*" OR "group-based" OR collab* OR collective OR "group-mediated" OR "group-led" OR cohes* OR "class" OR social OR "face to face")) AND (TITLE (alone OR home* OR "personal training" OR individual OR "non-group" OR "non-social" OR solo OR "without support" OR "without others") OR ABS (alone OR home* OR "personal training" OR individual OR "non-group" OR "non-social" OR solo OR "without support" OR "without others")) AND (TITLE ("physical activity" OR "exercise" OR adherence OR "compliance" OR "participation" OR "engagement" OR "physical fitness" OR "exercise frequency" OR "exercise intensity" OR "duration" OR "exercise volume" OR "MVPA" OR "vigorous physical activity" OR "moderate physical activity" OR "step count" OR "pedometer" OR "accelerometer" OR "activity tracker") OR ABS ("physical activity" OR "exercise" OR adherence OR "compliance" OR "participation" OR "engagement" OR "physical fitness" OR "exercise frequency" OR "exercise intensity" OR "duration" OR "exercise volume" OR "MVPA" OR "vigorous physical activity" OR "moderate physical

activity" OR "step count" OR "pedometer" OR "accelerometer" OR "activity tracker")) AND (TITLE ("physical activ*" OR "physical move*" OR "physical training" OR "physical strength" OR "quality of life" OR "gait" OR "physical performance" OR "physical fitness" OR exercise OR "mall-walk*" OR swim* OR walk* OR "resistance training" OR biking OR jog* OR jump* OR hik* OR danc* OR run* OR "walking group" OR yoga OR bike*) OR ABS ("physical activ*" OR "physical move*" OR "physical training" OR "physical strength" OR "quality of life" OR "gait" OR "physical performance" OR "physical fitness" OR exercise OR "mall-walk*" OR swim* OR walk* OR "resistance training" OR biking OR jog* OR jump* OR hik* OR danc* OR run* OR "walking group" OR yoga OR bike*)) AND (TITLE (intervention OR program* OR trial OR "exercise-program*" OR exposure) OR ABS (intervention OR program* OR trial OR "exercise-program*" OR exposure)) AND (TITLE (outcome OR benefit* OR improv* OR impact* OR change* OR effect* OR differ*) OR ABS (outcome OR benefit* OR improv* OR impact* OR change* OR effect* OR differ*)) AND (TITLE (psychosocial OR physical OR psychological OR "mental health" OR social OR health OR wellbeing OR function* OR physic*) OR ABS (psychosocial OR physical OR psychological OR "mental health" OR social OR health OR wellbeing OR function* OR physic*)) AND (TITLE (compar* OR versus OR vs) OR ABS (compar* OR versus OR vs)) AND NOT (TITLE (qualitative OR review OR "systematic review" OR "meta-analysis" OR "meta-synthesis") OR ABS (qualitative OR review OR "systematic review" OR "meta-analysis" OR "meta-synthesis"))

Filter peer-reviewed, Article and English

PROQUEST:

(ti(adult* OR "aged 18 and over" OR "aged 18 and older" OR "adults") OR ab(adult* OR "aged 18 and over" OR "aged 18 and older" OR "adults") OR kw(adult* OR "aged 18 and over" OR "aged 18 and older" OR "adults"))

AND

(ti(Group OR "with others" OR "with peers" OR "group-exercise" OR "peer-support*" OR communal OR "in-center" OR "in-centre" OR accomp* OR "group-interaction*" OR "group-based" OR collab* OR collective OR "group-mediated" OR "group-led" OR cohes* OR "class" OR social OR "face to face") OR ab(Group OR "with others" OR "with peers" OR "group-exercise" OR "peer-support*" OR communal OR "in-center" OR "in-centre" OR

accomp* OR "group-interaction*" OR "group-based" OR collab* OR collective OR "group-mediated" OR "group-led" OR cohes* OR "class" OR social OR "face to face") OR kw(Group OR "with others" OR "with peers" OR "group-exercise" OR "peer-support*" OR communal OR "in-center" OR "in-centre" OR accomp* OR "group-interaction*" OR "group-based" OR collab* OR collective OR "group-mediated" OR "group-led" OR cohes* OR "class" OR social OR "face to face"))

AND

(ti(Alone OR home* OR "personal training" OR individual OR "non-group" OR "non-social" OR solo OR "without support" OR "without others") OR ab(Alone OR home* OR "personal training" OR individual OR "non-group" OR "non-social" OR solo OR "without support" OR "without others") OR kw(Alone OR home* OR "personal training" OR individual OR "non-group" OR "non-social" OR solo OR "without support" OR "without others"))

AND

(ti("physical activity" OR "exercise" OR adherence OR "compliance" OR "participation" OR "engagement" OR "physical fitness" OR "adherence" OR "exercise frequency" OR "exercise intensity" OR "duration" OR "exercise volume" OR "MVPA" OR "vigorous physical activity" OR "moderate physical activity" OR "step count" OR "pedometer" OR "accelerometer" OR "activity tracker") OR ab("physical activity" OR "exercise" OR adherence OR "compliance" OR "participation" OR "engagement" OR "physical fitness" OR "adherence" OR "exercise frequency" OR "exercise intensity" OR "duration" OR "exercise volume" OR "MVPA" OR "vigorous physical activity" OR "moderate physical activity" OR "step count" OR "pedometer" OR "accelerometer" OR "activity tracker") OR kw("physical activity" OR "exercise" OR adherence OR "compliance" OR "participation" OR "engagement" OR "physical fitness" OR "adherence" OR "exercise frequency" OR "exercise intensity" OR "duration" OR "exercise volume" OR "MVPA" OR "vigorous physical activity" OR "moderate physical activity" OR "step count" OR "pedometer" OR "accelerometer" OR "activity tracker"))

AND

(ti("physical activ*" OR "physical move*" OR "physical training" OR "physical strength" OR "quality of life" OR "gait" OR "physical performance" OR "physical fitness" OR exercise

OR "mall-walk*" OR swim* OR walk* OR "resistance training" OR biking OR jog* OR jump* OR hik* OR danc* OR run* OR "walking group" OR yoga OR bike*) OR ab("physical activ*" OR "physical move*" OR "physical training" OR "physical strength" OR "quality of life" OR "gait" OR "physical performance" OR "physical fitness" OR exercise OR "mall-walk*" OR swim* OR walk* OR "resistance training" OR biking OR jog* OR jump* OR hik* OR danc* OR run* OR "walking group" OR yoga OR bike*) OR kw("physical activ*" OR "physical move*" OR "physical training" OR "physical strength" OR "quality of life" OR "gait" OR "physical performance" OR "physical fitness" OR exercise OR "mall-walk*" OR swim* OR walk* OR "resistance training" OR biking OR jog* OR jump* OR hik* OR danc* OR run* OR "walking group" OR yoga OR bike*))

AND

(ti(intervention OR program* OR trial OR "exercise-program*" OR exposure) OR ab(intervention OR program* OR trial OR "exercise-program*" OR exposure) OR kw(intervention OR program* OR trial OR "exercise-program*" OR exposure))

AND

(ti(Outcome OR benefit* OR improv* OR impact* OR change* OR effect* OR differ*) OR ab(Outcome OR benefit* OR improv* OR impact* OR change* OR effect* OR differ*) OR kw(Outcome OR benefit* OR improv* OR impact* OR change* OR effect* OR differ*))

AND

(ti(psychosocial OR physical OR psychological OR "mental health" OR social OR health OR wellbeing OR function* OR physic*) OR ab(psychosocial OR physical OR psychological OR "mental health" OR social OR health OR wellbeing OR function* OR physic*) OR kw(psychosocial OR physical OR psychological OR "mental health" OR social OR health OR wellbeing OR function* OR physic*))

AND

(ti(compar* OR versus OR vs) OR ab(compar* OR versus OR vs) OR kw(compar* OR versus OR vs))

AND

NOT

(ti(qualitative OR review OR "systematic review" OR "meta-analysis" OR "meta-synthesis") OR ab(qualitative OR review OR "systematic review" OR "meta-analysis" OR "meta-synthesis") OR kw(qualitative OR review OR "systematic review" OR "meta-analysis" OR "meta-synthesis"))

Only include Scholar Journals

Pubmed:

(adults[Title/Abstract]) AND (Group[Title/Abstract] OR others[Title/Abstract] OR "with peers"[Title/Abstract] OR "group-exercise"[Title/Abstract] OR "peer-support*"[Title/Abstract] OR communal[Title/Abstract] OR "in-center"[Title/Abstract] OR "in-centre"[Title/Abstract] OR accomp*[Title/Abstract] OR "group-interaction*"[Title/Abstract] OR "group-based"[Title/Abstract] OR collab*[Title/Abstract] OR collective[Title/Abstract] OR "group-mediated"[Title/Abstract] OR "group-led"[Title/Abstract] OR cohes*[Title/Abstract] OR class[Title/Abstract] OR social[Title/Abstract] OR "face to face"[Title/Abstract]) AND (Alone[Title/Abstract] OR home*[Title/Abstract] OR "personal training"[Title/Abstract] OR individual[Title/Abstract] OR "non-group"[Title/Abstract] OR "non-social"[Title/Abstract] OR solo[Title/Abstract] OR "without support"[Title/Abstract]) AND ("physical activity"[Title/Abstract] OR exercise[Title/Abstract] OR adherence[Title/Abstract] OR compliance[Title/Abstract] OR participation[Title/Abstract] OR engagement[Title/Abstract] OR "physical fitness"[Title/Abstract] OR "exercise frequency"[Title/Abstract] OR "exercise intensity"[Title/Abstract] OR duration[Title/Abstract] OR "exercise volume"[Title/Abstract] OR MVPA[Title/Abstract] OR "vigorous physical activity"[Title/Abstract] OR "moderate physical activity"[Title/Abstract] OR "step count"[Title/Abstract] OR pedometer[Title/Abstract] OR accelerometer[Title/Abstract] OR "activity tracker"[Title/Abstract]) AND ("physical activ*"[Title/Abstract] OR "physical move*"[Title/Abstract] OR "physical training"[Title/Abstract] OR "physical strength"[Title/Abstract] OR "quality of life"[Title/Abstract] OR gait[Title/Abstract] OR "physical performance"[Title/Abstract] OR "physical fitness"[Title/Abstract] OR exercise[Title/Abstract] OR "mall-walking"[Title/Abstract] OR swimming[Title/Abstract] OR walking[Title/Abstract] OR "resistance training"[Title/Abstract] OR

biking[Title/Abstract] OR jogging[Title/Abstract] OR jumping[Title/Abstract] OR hiking[Title/Abstract] OR dancing[Title/Abstract] OR running[Title/Abstract] OR "walking group"[Title/Abstract] OR yoga[Title/Abstract] OR biking[Title/Abstract]) AND (intervention[Title/Abstract] OR program*[Title/Abstract] OR trial[Title/Abstract] OR "exercise-program*"[Title/Abstract] OR exposure[Title/Abstract]) AND (Outcome[Title/Abstract] OR benefit*[Title/Abstract] OR improv*[Title/Abstract] OR impact*[Title/Abstract] OR change*[Title/Abstract] OR effect*[Title/Abstract] OR differ*[Title/Abstract]) AND (psychosocial[Title/Abstract] OR physical[Title/Abstract] OR psychological[Title/Abstract] OR "mental health"[Title/Abstract] OR social[Title/Abstract] OR health[Title/Abstract] OR wellbeing[Title/Abstract] OR function*[Title/Abstract] OR physic*[Title/Abstract]) AND (compar*[Title/Abstract] OR versus[Title/Abstract] OR vs[Title/Abstract]) NOT (qualitative[Title/Abstract] OR review[Title/Abstract] OR "systematic review"[Title/Abstract] OR "meta-analysis"[Title/Abstract] OR "meta-synthesis"[Title/Abstract])

Science Direct:

("adults") AND ("group exercise") AND ("individual" OR "home") AND ("physical activity" OR "exercise") AND ("intervention") AND ("outcome") AND ("psychosocial")

Medline

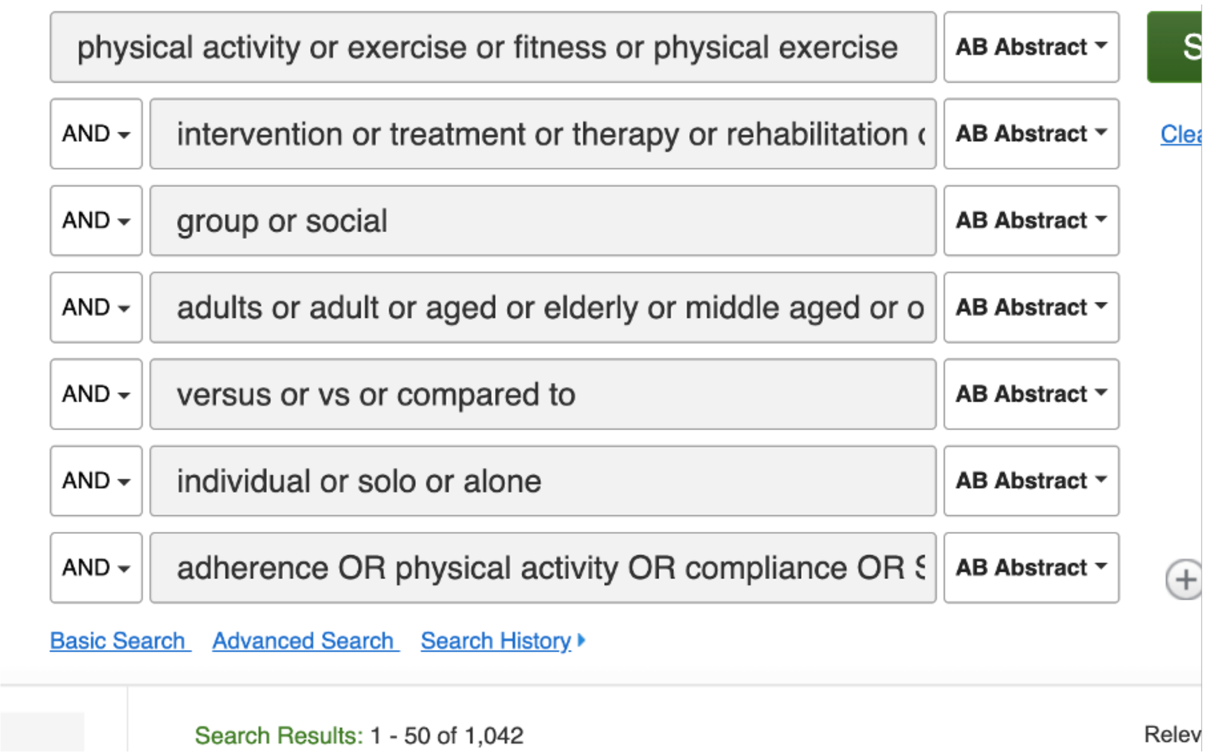
physical activity or exercise or fitness or physical exercise
AB Abstract
AND
intervention or treatment or therapy or rehabilitation
AB Abstract
AND
group or social
AB Abstract
AND
adults or adult or aged or elderly or middle aged or
AB Abstract
AND
versus or vs or compared to
AB Abstract
AND
individual or solo or alone
AB Abstract
AND
adherence OR physical activity OR compliance OR
AB Abstract
Basic Search
Advanced Search
Search History
Search Results: 1 - 50 of 1,042

PSYCINFO

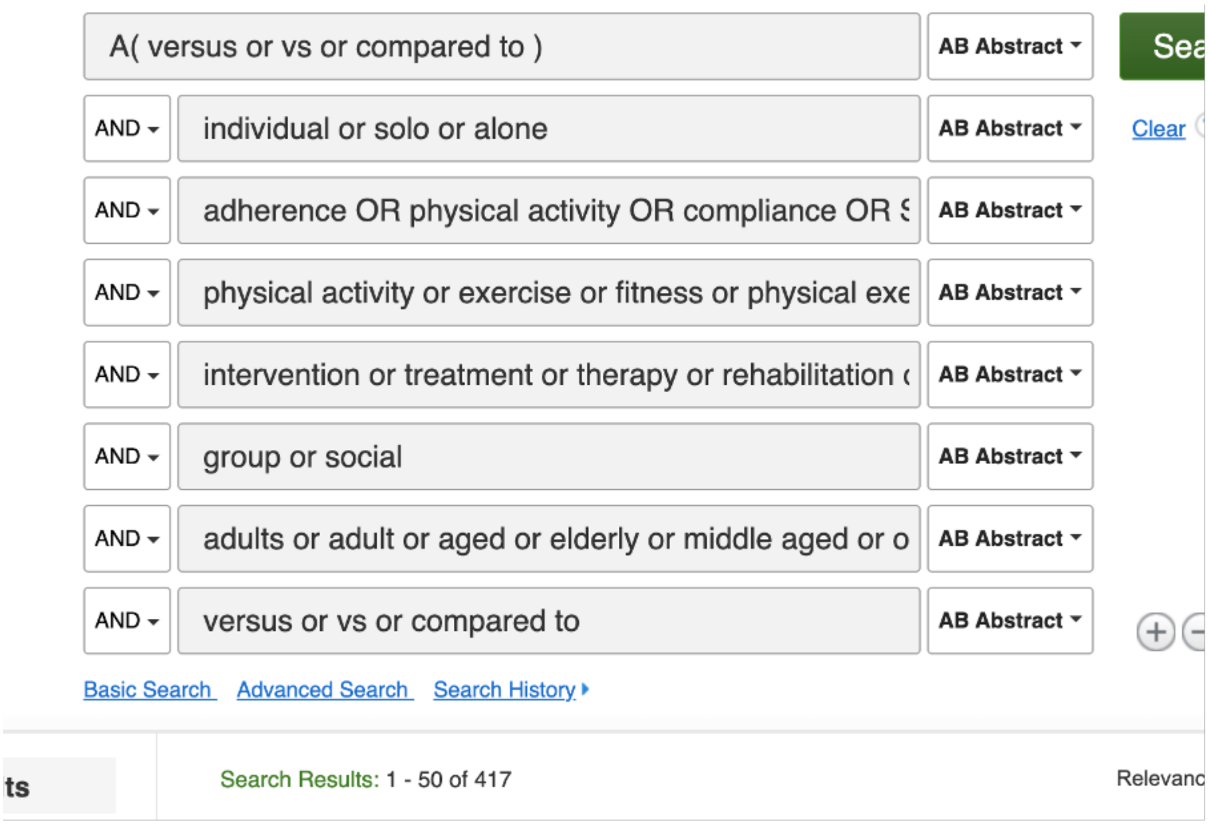

Restriction: Academic journals

Google Scholar:

"group OR collective vs. individual" AND "physical activity" AND “Adult” AND "versus OR compared to" AND "change OR intervention OR impact OR Effect OR Promote"

Other Sources (e.g. backwards and forwards search)

Certainty of evidence (GRADE assessment)

Table S1 summarizes the GRADE assessment of evidence certainty for physical activity, functional, psychosocial, and health outcomes, stratified by study design (RCT/quasi-experimental vs. non-experimental). Certainty ratings reflect judgments across GRADE domains, including risk of bias, inconsistency, indirectness, imprecision, and publication bias, and are reported as overall certainty ratings for each outcome domain.

Table S1

GRADE Assessment for Behavioral, Functional, Psychosocial and Health Outcomes.

No of studies	Design	Risk of bias	Inconsistency	Indirectness	Imprecision	Publication bias	Certainty (overall score)
**Outcome:** Physical Activity							
116 effects, 16,886 participants	RCT/Quasi-RCT	Moderate	Serious	Serious	Serious	Non-Serious	MODERATE ⊕⊕⊕◯
**Outcome:** Physical Activity							
9 effects 5,156 participants	Non-Experimental	Serious	Serious	Serious	Serious	Non-Serious	LOW ⊕◯◯◯

**Outcome:** Functional							
140 effects, 19,707 participants	RCT/Quasi-RCT	Serious	Moderate	Non-serious	Moderate	Serious	MODERATE ⊕⊕⊕◯
**Outcome:** Functional							
17 effects, 6,066 participants	Non-Experimental	Serious	Moderate	Moderate	Moderate	Serious	MODERATE ⊕⊕⊕◯
**Outcome:** Psychosocial Outcomes							
93 effects, 14,307 participants	RCT/Quasi-RCT	Serious	Non-serious	Serious	Serious	Non-serious	LOW ⊕◯◯◯
12 effects, 3,916 participants	Non-experimental	Serious	Non-serious	Serious	Moderate	Serious	LOW ⊕◯◯◯

Outcome: Health Outcomes

116 effects, 16,209 participants	RCT/Quasi-RCT	Serious	Moderate	Non-serious	Serious	Non-serious	MODERATE ⊕⊕⊕○
21 effects, 15,398 participants	Non-experimental	Serious	Moderate	Moderate	Serious	Serious	MODERATE ⊕⊕⊕○

Funnel plots for functional, psychosocial, and health outcomes

Supplementary Figures S1–S3 present sunset-enhanced funnel plots for functional, psychosocial, and health outcomes. These plots illustrate the distribution of study-level effect sizes in relation to statistical power and precision and provide a visual assessment of evidential strength and potential small-study effects across outcome domains.

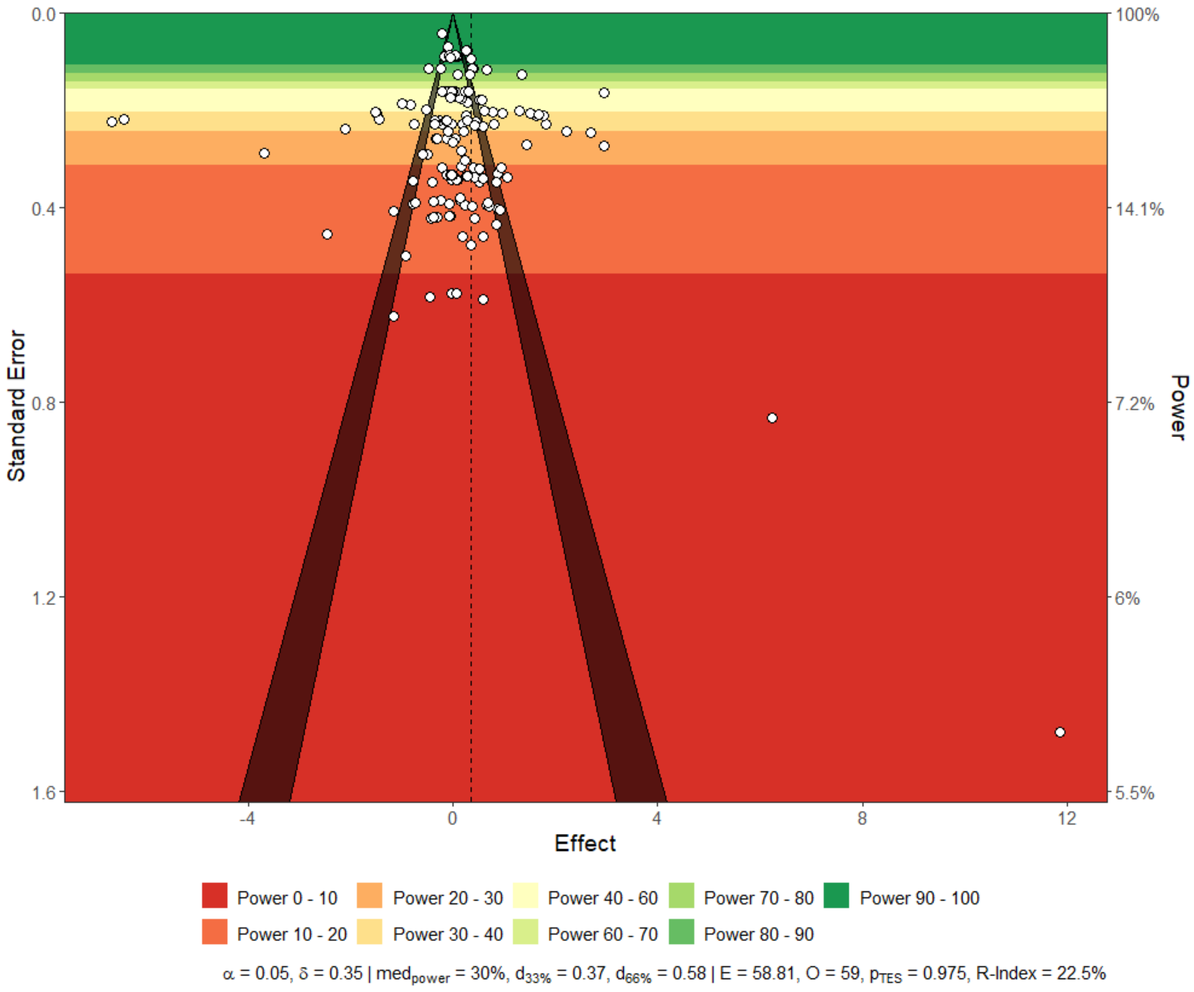

Figure S1. Sunset enhanced funnel plot of included studies for functional outcomes

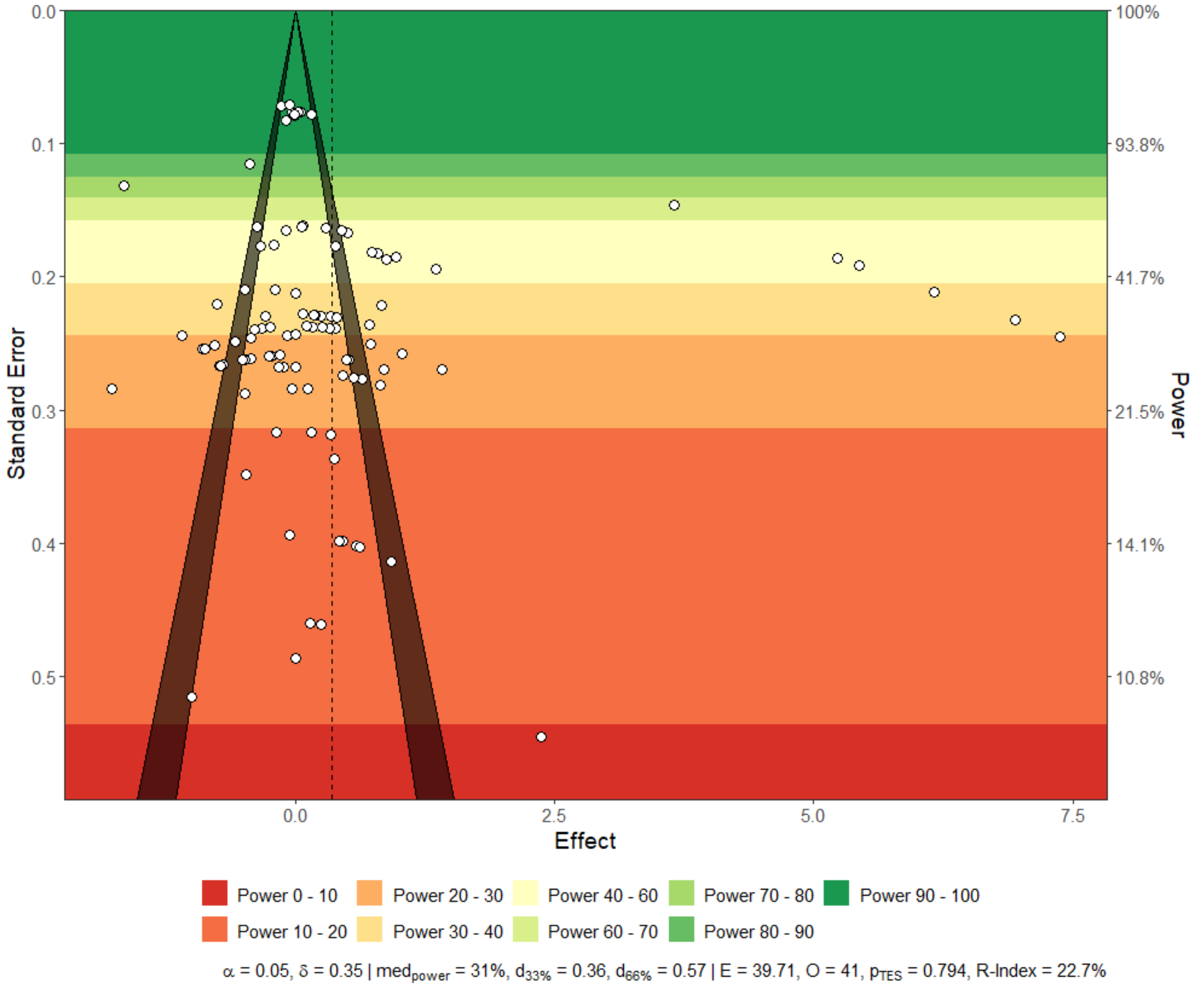

Figure S2. Sunset enhanced funnel plot of included studies for psychosocial outcomes

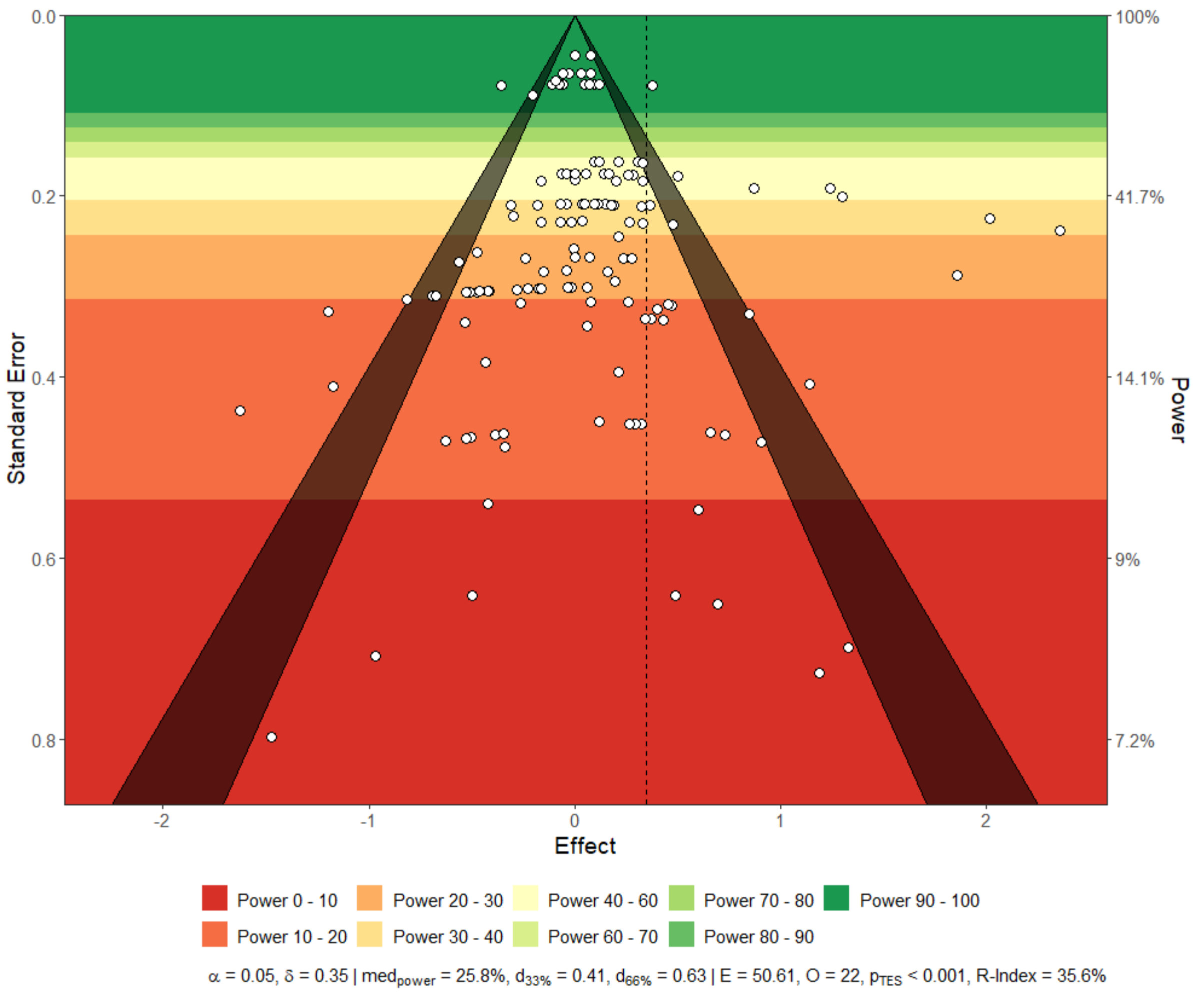

Figure S3. Sunset enhanced funnel plot of included studies for health outcomes

Robustness and sensitivity analyses

Supplementary Tables S2–S5 report three-level random-effects meta-analytic estimates (Cohen's *d*) comparing group-based and individual interventions across outcome domains, shown before and after outlier removal with corresponding test statistics, exact *p*-values, and 95% confidence intervals.

Subsequently, Supplementary Figures S4–S11 present diagnostic plots used to evaluate model assumptions and robustness of the three-level random-effects meta-analytic models across outcome domains. Cook's distance plots identify potentially influential observations, while standardized residual plots assess the distribution and magnitude of residuals. These diagnostics were examined to assess the impact of individual studies on model estimates and to support the robustness of the reported results.

Table S2
Physical activity model estimates before and after outlier removal

Model	Estimate	SE	t-value	df	p-value	CI Lower	CI Upper
Main model	0.0858	0.0744	1.1541	124	0.2507	−0.0614	0.2330
Outliers removed	0.0915	0.0591	1.548	118	0.1243	−0.0255	0.2085

Note. All analyses used a three-level random-effects model (Cheung, 2014, 2019). Effect sizes represent standardized mean differences between individual and group-based formats.

Table S2
Results for Functional Outcomes Before and After Outlier Removal

Model	Estimate	SE	t-value	df	p-value	CI Lower	CI Upper
Main model	0.0587	0.1444	0.4064	156	0.685	−0.2266	0.344
Outliers removed	0.1632	0.0649	2.5139	150	0.013	0.0349	0.2914

Note. All analyses used a three-level random-effects model (Cheung, 2014, 2019). Effect sizes represent standardized mean differences between individual and group-based formats.

Table S4.
Results for Psychosocial outcomes before and after outlier removal

Model	Estimate	SE	t-value	df	p-value	CI Lower	CI Upper
Main model	0.2919	0.2334	1.251	103	0.2138	−0.1709	0.7548
Outliers removed	0.2205	0.1420	1.5532	91	0.1239	−0.0615	0.5025

Note. All analyses used a three-level random-effects model (Cheung, 2014, 2019). Effect sizes represent standardized mean differences between individual and group-based formats.

Table S5.
Results for Health Outcomes Before and After Outlier Removal

Model	Estimate	SE	t-value	df	p-value	CI Lower	CI Upper
Main model	0.1249	0.0745	1.6762	136	0.096	−0.0225	0.2723
Outliers removed	0.1042	0.0538	1.9384	124	0.0549	−0.0022	0.2106

Note. All analyses used a three-level random-effects model (Cheung, 2014, 2019). Effect sizes represent standardized mean differences between individual and group-based formats.

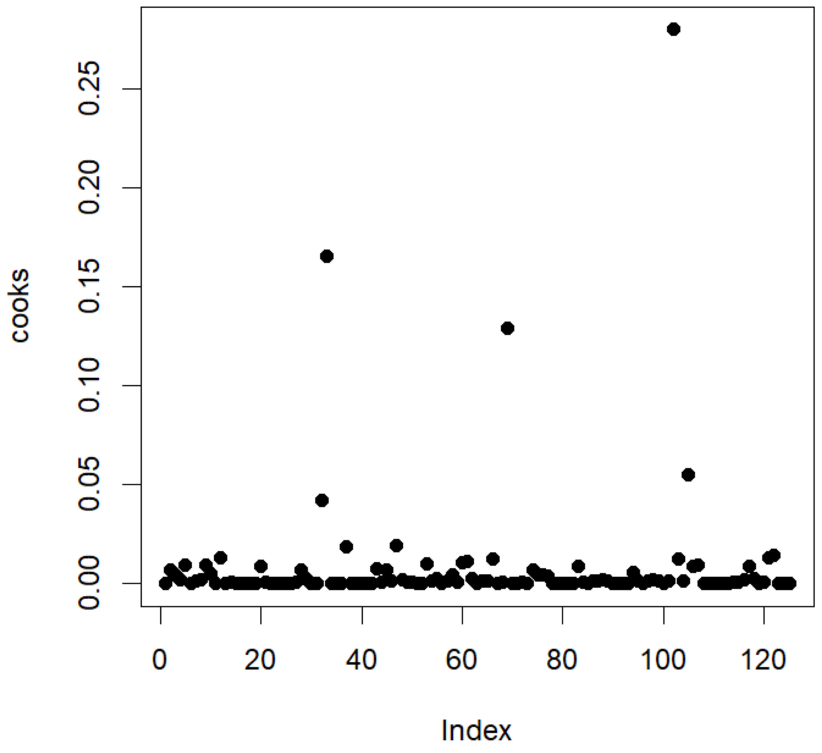

Figure S4. Cook's distance diagnostic plot for physical activity model

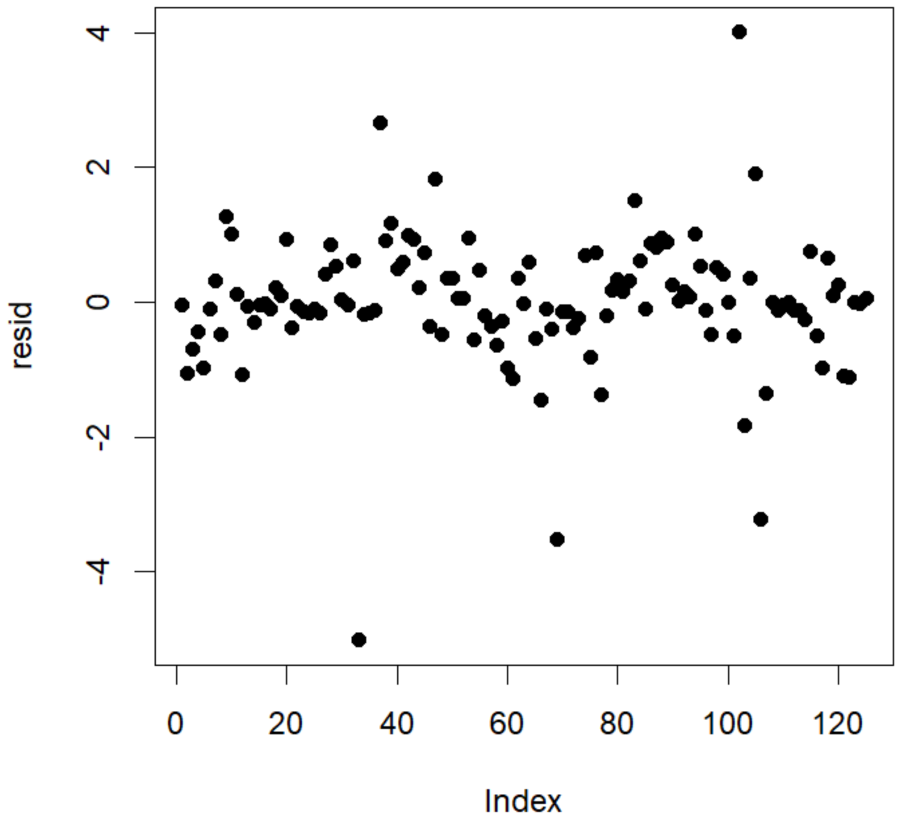

Figure S5. Standardized residuals diagnostic plot for physical activity model

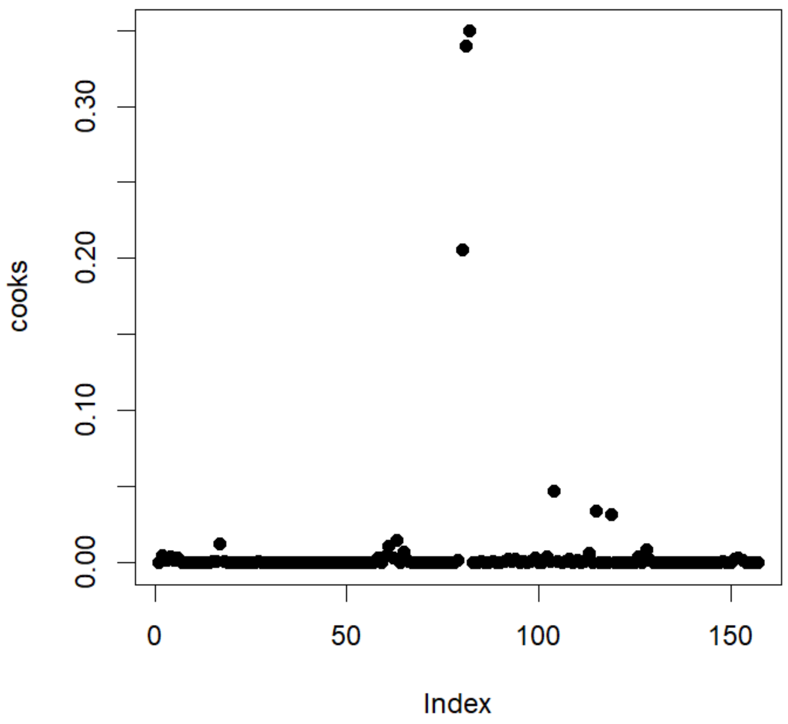

Figure S6. Cook’s distance diagnostic plot for functional outcomes

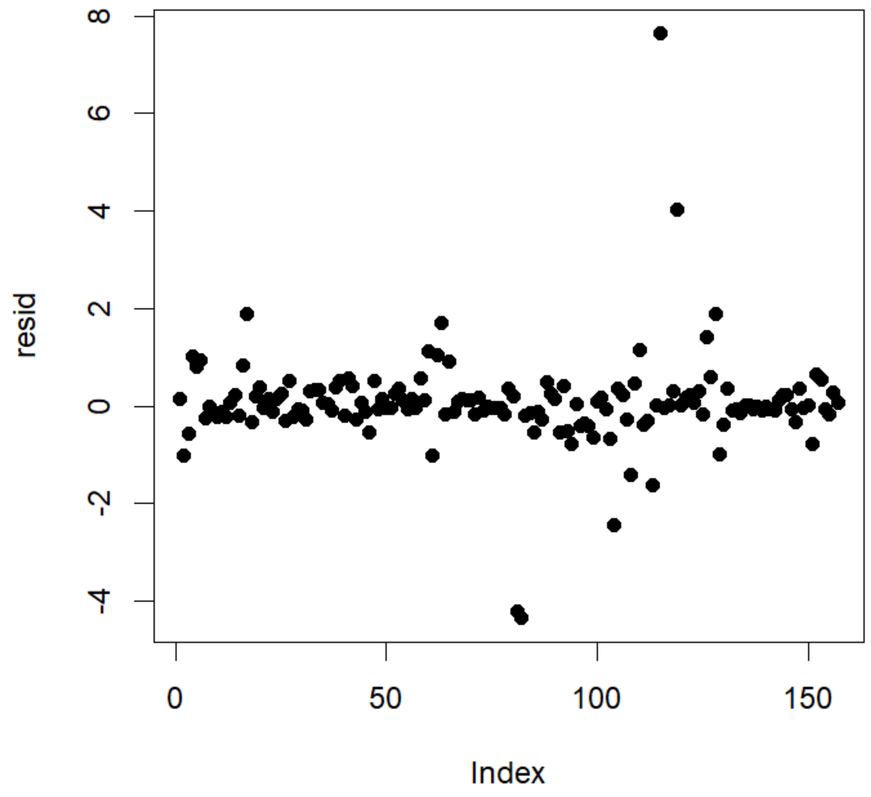

Figure S7. Standardized residuals diagnostic plot for functional outcomes

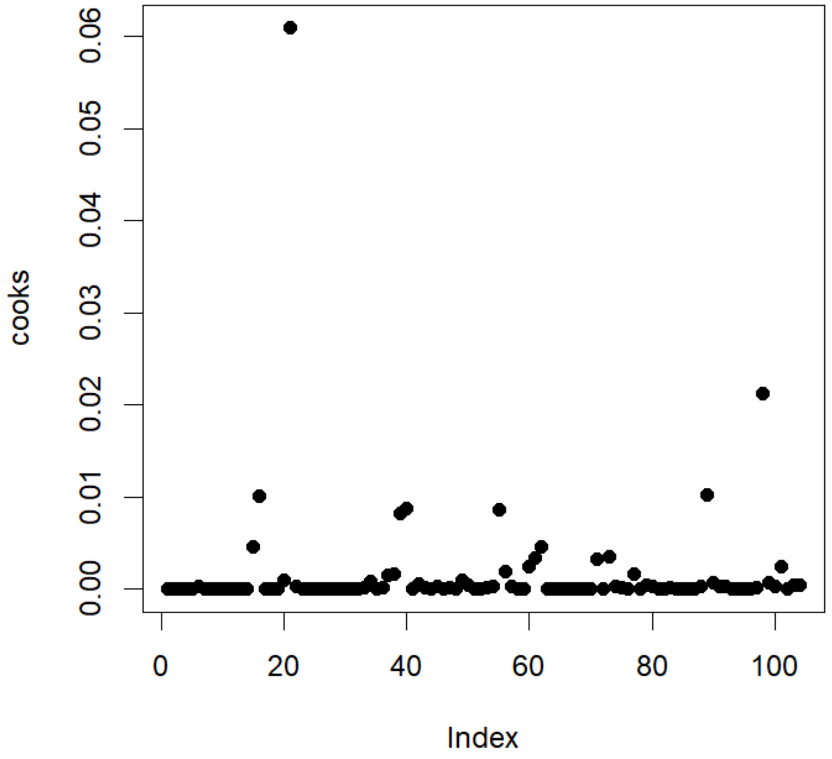

Figure S8. Cook's distance diagnostic plot for psychosocial outcomes

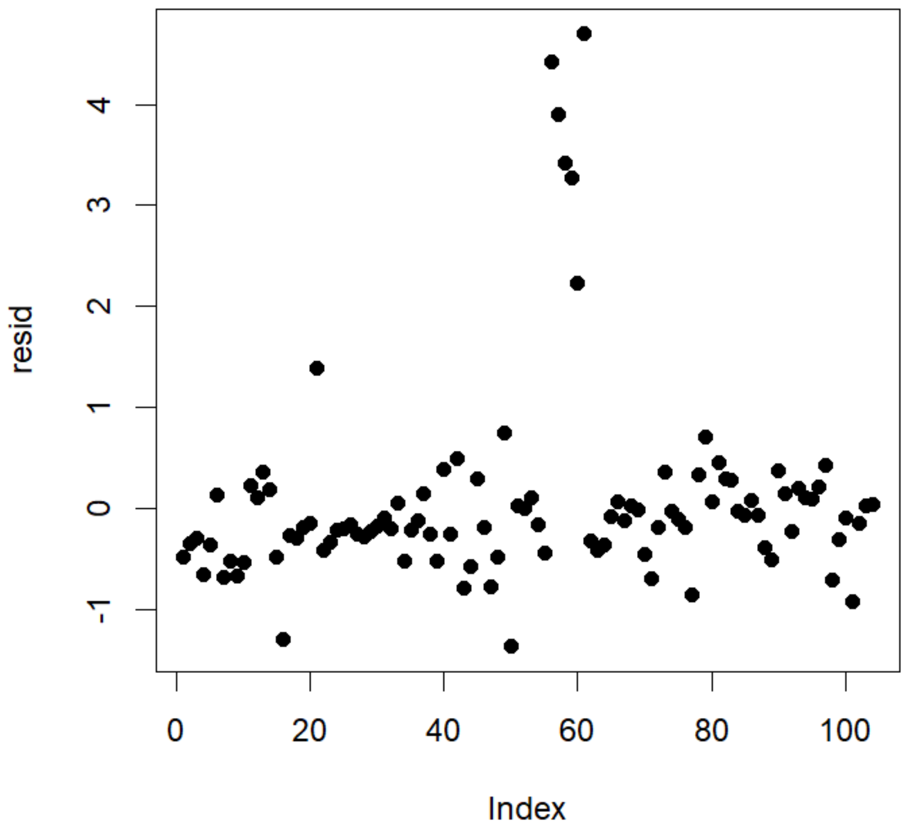

Figure S9. Standardized residuals diagnostic plot for psychosocial outcomes

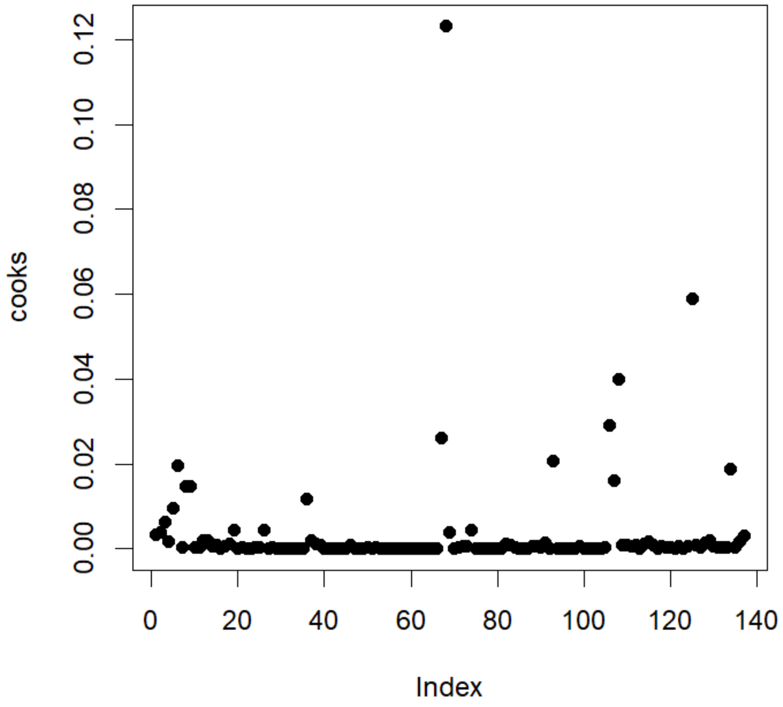

Figure S10. Cook’s distance diagnostic plot for health outcomes

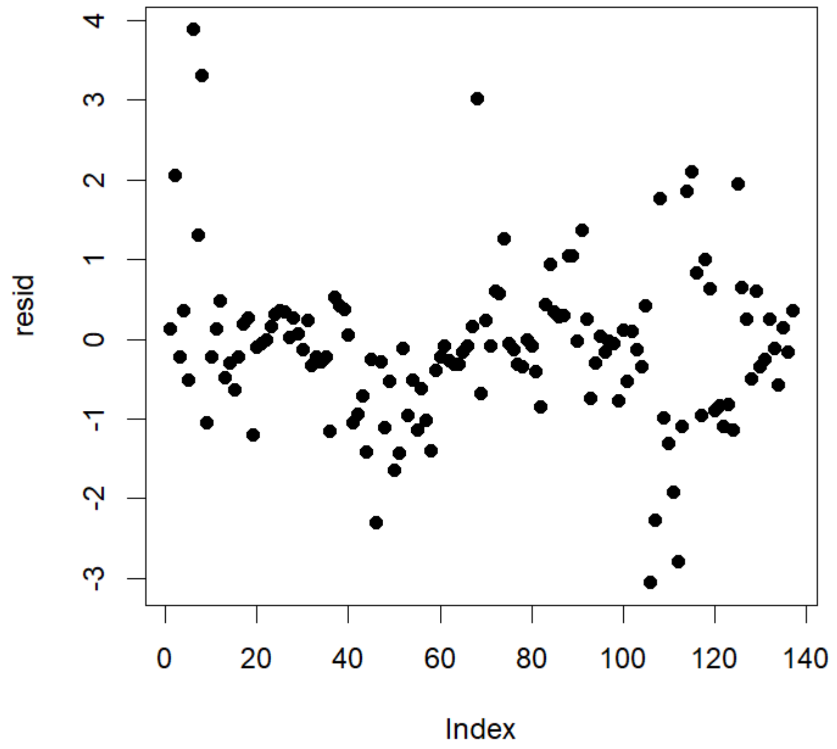

Figure S11. Standardized residuals diagnostic plot for health outcomes

Variance Components and Prediction Intervals

Table S6 reports 95% prediction intervals, variance components at levels 2 and 3, and the proportion of variance explained by moderators (pseudo-R^2) for functional, psychosocial, and health outcomes. Estimates are derived from three-level random-effects models and quantify between-study and within-study heterogeneity across outcome domains.

Table S6
Prediction Intervals, Variance Components, and Variance Accounted for by Moderators (Pseudo-R^2)

Outcome	95 % PI Lower	95 % PI Upper	Level 2 Variance (σ^2)	LRT	***p***	pseudo-R^2	Level 3 Variance (σ^2)	LRT	***p***	pseudo-R^2
Functional	−2.408	2.526	1.168	2531.7	< .001	.067	0.371	2531.7	< .001	> .999
Psychosocial	−2.338	2.922	1.404	362.872	< .001	< .001	0.300	120.823	< .001	.947
Health	−0.722	0.972	0.090	91.873	< .001	< .001	0.088	31.819	< .001	.161

Note. 95 % PI = 95 % prediction interval; LRT = likelihood-ratio test. All estimates are derived from three-level random-effects models (Cheung, 2014, 2019).

Part 2: Protocol Adherence and Deviations (PROSPERO CRD42021271452)

Table S7 summarizes adherence to the registered PROSPERO protocol (CRD42021271452) and documents any deviations between the original protocol and the current implementation. For each protocol element, the table reports the original specification, the implemented approach, the status of adherence or deviation, and the rationale for any changes to ensure transparency and reproducibility.

Table S7

Summary Of PROSPERO Protocol Adherence and Deviations (CRD42021271452)

Protocol Element	Original Specification	Current Implementation	Status / Deviation	Rationale
Title	Comparing the effectiveness of individual and group approaches for promoting physical activity, physical health, and psychosocial wellbeing	Solo or team? A meta-analysis comparing individual and group-based approaches on physical activity, functional, psychosocial, and health outcomes	Deviation 1	The title was revised to more clearly reflect the scope of the review and the outcomes examined. The updated wording specifies the comparison between individual and group-based approaches, highlights all four outcome

				domains (physical activity, functional, psychosocial, health), and aligns terminology with the analyses.
Objectives	Compare individual vs. group approaches for promoting physical activity, physical health, and psychosocial wellbeing.	Same comparison retained. Delivery mode (online vs. in-person) was explicitly included as a moderator, and sub-questions were stated to improve clarity.	✓Clarification (not a deviation)	Online group formats and moderation analyses were already conceptually included in the protocol. The refinement operationalizes this in the manuscript for greater transparency and specificity. No change in objectives.
Eligibility Criteria	Adults (≥18 yrs); quantitative	Same inclusion criteria applied.	✓ Adhered	—

	studies comparing group vs. individual physical activity.			
Search Strategy	Databases: Web of Science, Scopus, ProQuest, PubMed, PsycINFO, ScienceDirect, MEDLINE, CENTRAL; searches in English & German; end date April 2022.	Databases as registered; search updated to 2025; restricted to English only.	Deviation 2 and 3	(1) Extended timeframe. (2) Limited to English-language studies to ensure reliability (no second German coder).
Risk-of-Bias / Quality Assessment	QualSyst and Q-SSP checklists.	QualSyst plus GRADE ratings for evidence certainty.	Deviation 4- (added component)	Enhanced transparency and comparability across domains.
Statistical Analyses	Three-level random-effects model (Cheung, 2014, 2019); report I^2, τ^2, H^2; publication bias via Egger's test.	Same model plus variance components, pseudo-R^2, prediction intervals, and z-curve analysis; included TOST	Deviation 5- (extended analytic detail)	Added to interpret equivalence and improve bias diagnostics.

		equivalence testing.		
Outcomes	Physical activity (primary); psychological, social, and physical health (secondary).	In the protocol, psychological and social outcomes were listed separately. During synthesis, these domains were conceptually merged and reported as ‘psychosocial outcomes.’ Additionally, the protocol category ‘physical health’ was separated into functional and health outcomes to reflect how results were reported in the included studies.	Deviation 6- **(Terminology clarification and analytic categorization**.)	Psychological and social outcomes were conceptually merged and reported as ‘psychosocial outcomes’ because the included measures typically captured overlapping constructs (e.g., well-being, quality of life). Physical health outcomes were separated into functional and health outcomes to better reflect how results were reported in the included studies. No outcomes were omitted or

				renamed beyond this consolidation.
Publication Bias Assessment	Egger’s test and funnel plots.	Extended with z-curve analysis (Brunner & Schimmack, 2020).	Deviation 7 (methodological enhancement)	Enhanced robustness.
Registration & Transparency	PROSPERO CRD42021271452 (Aug 2021).	Cited in Methods (pp. 21–22).	✔ Adhered	
Additional Comments	Additional changes included slight adaption to contributing authors			

References

Cheung, M. W. L. (2014). Modeling dependent effect sizes with three-level meta-analyses. *Journal of Educational and Behavioral Statistics, 39*(2), 135–157.

Brunner, J., & Schimmack, U. (2020). *Estimating population mean power under conditions of heterogeneity and selection for significance. Meta-Psychology, 4*, Article MP.2018.874. https://doi.org/10.15626/MP.2018.874

Kritz, M., Ridell, H., Olsen, D., Harden, S. M., Burke, S. M., Ntoumanis, N., & Thøgersen-Ntoumani, C. (2025). Data and materials for "Solo or team? A meta-analysis comparing individual and group-based approaches on physical activity, functional, psychosocial, and health outcomes". Open Science Framework. https://doi.org/10.17605/OSF.IO/XT2G4
