# Supplementary material for: Individual versus group-based interventions: a systematic review and meta-analysis of physical activity, functional, psychosocial and health outcomes
Source: Nat Hum Behav. 2026 Apr 15;10(6):1109–21. doi: 10.1038/s41562-026-02429-0 (PMC13290480; doi:10.1038/s41562-026-02429-0)
Supplement: Supplementary file 2 — Reporting Summary [file 41562_2026_2429_MOESM2_ESM.pdf]

nature portfolio

Corresponding author(s): Marlene Kritz

Last updated by author(s): 29.1.2026 by Marlene Kritz

Reporting Summary

Nature Portfolio wishes to improve the reproducibility of the work that we publish. This form provides structure for consistency and transparency in reporting. For further information on Nature Portfolio policies, see our Editorial Policies and the Editorial Policy Checklist.

Statistics

For all statistical analyses, confirm that the following items are present in the figure legend, table legend, main text, or Methods section.

n/a	Confirmed	
☐	☒	The exact sample size (*n*) for each experimental group/condition, given as a discrete number and unit of measurement
☐	☒	A statement on whether measurements were taken from distinct samples or whether the same sample was measured repeatedly
☐	☒	The statistical test(s) used AND whether they are one- or two-sided<br>*Only common tests should be described solely by name; describe more complex techniques in the Methods section.*
☐	☒	A description of all covariates tested
☐	☒	A description of any assumptions or corrections, such as tests of normality and adjustment for multiple comparisons
☐	☒	A full description of the statistical parameters including central tendency (e.g. means) or other basic estimates (e.g. regression coefficient) AND variation (e.g. standard deviation) or associated estimates of uncertainty (e.g. confidence intervals)
☒	☐	For null hypothesis testing, the test statistic (e.g. *F*, *t*, *r*) with confidence intervals, effect sizes, degrees of freedom and *P* value noted<br>*Give P values as exact values whenever suitable.*
☒	☐	For Bayesian analysis, information on the choice of priors and Markov chain Monte Carlo settings
☐	☒	For hierarchical and complex designs, identification of the appropriate level for tests and full reporting of outcomes
☐	☒	Estimates of effect sizes (e.g. Cohen's *d*, Pearson's *r*), indicating how they were calculated

Our web collection on statistics for biologists contains articles on many of the points above.

Software and code

Policy information about availability of computer code

Data collection	An automated research screening tool (Research Screener) was used to assist with title and abstract screening, alongside manual screening and full-text assessment by the research team. Excel was used to assist with data extraction.
Data analysis	All analyses were conducted in R (version 4.3.1) using established meta-analytic packages (metafor, metaSEM, and metaviz packages). Scripts/code used for the analysis are available on the Open Science Framework at https://doi.org/10.17605/OSF.IO/XT2G4.

For manuscripts utilizing custom algorithms or software that are central to the research but not yet described in published literature, software must be made available to editors and reviewers. We strongly encourage code deposition in a community repository (e.g. GitHub). See the Nature Portfolio guidelines for submitting code & software for further information.

Data

Policy information about availability of data

All manuscripts must include a data availability statement. This statement should provide the following information, where applicable:

- Accession codes, unique identifiers, or web links for publicly available datasets
- A description of any restrictions on data availability
- For clinical datasets or third party data, please ensure that the statement adheres to our policy

The dataset generated and analyzed during this meta-analysis and additional materials, are publicly available via the Open Science Framework (OSF) at https://doi.org/10.17605/OSF.IO/XT2G4

Research involving human participants, their data, or biological material

Policy information about studies with human participants or human data. See also policy information about sex, gender (identity/presentation), and sexual orientation and race, ethnicity and racism.

Reporting on sex and gender	This meta-analysis synthesizes data from previously published studies. Where reported by the original authors, sex and gender were extracted and considered as potential moderators. However, not all included studies disaggregated results by sex or gender. No new participant data were collected
Reporting on race, ethnicity, or other socially relevant groupings	Race, ethnicity, or other socially constructed groupings were not consistently reported across included studies. Where relevant and available, this information was extracted, but no new data were collected for this meta-analysis.
Population characteristics	Population characteristics (e.g., clinical vs. non-clinical, age group, delivery mode) were extracted based on the information reported in the original studies and analyzed as potential moderators.
Recruitment	No participants were recruited for this study. All data were extracted from previously published research. Recruitment procedures were those of the original studies and are reported therein.
Ethics oversight	As this is a meta-analysis of published data, no new ethical approval was required.

Note that full information on the approval of the study protocol must also be provided in the manuscript.

Field-specific reporting

Please select the one below that is the best fit for your research. If you are not sure, read the appropriate sections before making your selection.

☐ Life sciences ☒ Behavioural & social sciences ☐ Ecological, evolutionary & environmental sciences

For a reference copy of the document with all sections, see nature.com/documents/nr-reporting-summary-flat.pdf

Behavioural & social sciences study design

All studies must disclose on these points even when the disclosure is negative.

Study description	This was a quantitative meta-analysis of published studies comparing individual versus group-based physical activity interventions. A three-level random-effects model was used.
Research sample	The sample consisted of 71 published studies reporting on adult populations from various countries. Sample characteristics (e.g., age, sex, delivery mode) were extracted where available and analyzed as moderators. No new data were collected.
Sampling strategy	Studies were selected through systematic searches in peer-reviewed databases using predefined inclusion criteria. No additional sampling was conducted. A sensitivity analysis was conducted to address potential bias.
Data collection	Research Screener was used for abstract screening. All data were extracted manually from published studies and recorded in structured Excel spreadsheets. Data were manually extracted by two independent reviewers using structured coding sheets. Discrepancies were resolved through discussion. Study characteristics and outcome variables to calculate effect sizes were coded.
Timing	The final literature search was completed on 19 March 2024.
Data exclusions	Outlier studies were excluded in sensitivity analyses based on pre-specified criteria (e.g., extreme effect sizes with large standard errors). All decisions were transparently reported.
Non-participation	Not applicable. No new participants were recruited; all data were drawn from published sources.
Randomization	Not applicable. This was a meta-analysis.

Reporting for specific materials, systems and methods

We require information from authors about some types of materials, experimental systems and methods used in many studies. Here, indicate whether each material, system or method listed is relevant to your study. If you are not sure if a list item applies to your research, read the appropriate section before selecting a response.

Materials & experimental systems

n/a	Involved in the study
☒	☐ Antibodies
☒	☐ Eukaryotic cell lines
☒	☐ Palaeontology and archaeology
☒	☐ Animals and other organisms
☒	☐ Clinical data
☒	☐ Dual use research of concern
☒	☐ Plants

Methods

n/a	Involved in the study
☒	☐ ChIP-seq
☒	☐ Flow cytometry
☒	☐ MRI-based neuroimaging

Plants

Seed stocks	Not applicable
Novel plant genotypes	Not applicable
Authentication	Not applicable
