# Supplementary material for: Individual versus group-based interventions: a systematic review and meta-analysis of physical activity, functional, psychosocial and health outcomes
Source: Nat Hum Behav. 2026 Apr 15;10(6):1109–21. doi: 10.1038/s41562-026-02429-0 (PMC13290480; doi:10.1038/s41562-026-02429-0)
Supplement: Supplementary file 4 — Supplementary Tables 1 [file 41562_2026_2429_MOESM4_ESM.pdf]

Extended Data Table 1

Key Characteristics of Included Studies

Authors, year, and country	Study sample (N, age category, health status, fitness status)	Study setting	Study design	Exercise type, frequency, duration	Condition characteristics<br><br>(Group: delivery location, Group type: contact mode, number of contacts, type of group leader, group size)<br><br>Individual: Setting, contact mode, number of contacts)	Intervention/study duration (weeks)	Outcomes assessed	Qual Syst rating
Opdenacker et al., 2011, Belgium	N=120, older adults 60+, healthy, previously sedentary or insufficiently active<br><br>N=186	Community	Experimental-RCT	Exercise:<br><br>Mixed- Moderate Aerobic<br><br>Strength & Flexibility Training, 3 times/week, 40 minutes	Group condition: Center-based, true face-face group: 3 times/week, Qualified exercise instructor, group size: 10<br><br>Individual condition: Home-based, contact mode: Virtual-SMS or Telephone contact, number of contacts: 1-3 times/month	44 weeks	Physical Activity: % sessions attended<br><br>Functional: Number of bicep curls in 30 seconds, number of stands in 30 seconds, vertical jump, muscular fitness, peak torque recorded using six contractions at 240 deg/s, strength endurance<br><br>Health: BMI, waist circumference, % body fat, blood pressure, time to exhaustion, VO2max, exercise capacity.	0.73
Carmelli et al., 2006, Israel	N=63, older adults 75+, currently have a clinical condition, post-	Healthcare	Experimental-RCT	Exercise: Mixed-Moderate Aerobic, Strength & Flexibility	Group condition: health care center-based (physical therapy clinic), standard face-face group contact, 3 times/week, health professional	14 weeks	Physical Activity: compliance/drop-out rate	0.69

	operative, not specified			training 3 times/week, 50 minutes	(licensed physical therapist), group size: 7-10<br><br>Individual condition: Home-based, contact mode: Mixed- personal and telephone, number of contacts: 1-3 times/month.			
Cowie et al., 2011, United Kingdom	N=60, older adults 60+, clinical condition (left ventricular systolic dysfunction), not specified	Healthcare	Experimental- RCT	Exercise: Moderate Aerobic, Training, 2 times/week, 60 minutes	Group condition: health care center-based (hospital), standard face-face group, 2 times/week, health professional (physiotherapist), group size: not specified<br><br>Individual condition: Home-based, contact mode: telephone, number of contacts: 2 times/month	8 weeks	Physical Activity: Number of hours/standing/day, steps/day	0.91
Liu et al, 2021, USA	N=60, older adults 60+, Healthy (The majority,81% perceived themselves as healthy), all participants fit to walk), not specified	online	Experimental- RCT	Exercise: Moderate Aerobic Training (Walking), no prescription (steps/day)	Group condition: online, online community group with interactive component, frequency: Other- only social group feedback/interaction, no leader, group size: Not specified but assuming it is more than dyad as the authors refer to participants communicating with participants (i.e., plural)<br><br>Individual condition: online, contact mode: Virtual-SMS or Telephone contact, n contacts: 1-2 times/week	16 weeks	Physical activity: steps/day<br><br>Psychosocial: social engagement, exercise self-efficacy	0.84

Chown et al., 2008, United Kingdom	N=239, adults 18+ (18-65), currently have a clinical condition (chronic back pain), not specified.	Healthcare	Observational-longitudinal	Exercise: Flexibility and Strength Training, 2 times/month, 30 minutes	Group condition: health care center-based, standard face-face group, 2 times/month, group leader: health professional (physiotherapist), group size: 80<br><br>Individual condition: center-based, contact mode: face-face, 2 times/month	30 weeks	Physical activity: program attendance	0.78
Sajatovic et al., 2017, USA	N=30, older adults 60+, currently have clinical condition (Parkinsons disease and co-morbid depression), not specified	Healthcare	Experimental-RCT	Exercise: Mixed-Moderate Aerobic, Strength & Flexibility training, 3 times/week, 60 minutes	Group condition: health care center-based, true face-face group, 3 times/week, group leader: qualified exercise instructor and health professional (nurse), 7-8<br><br>Individual condition: center-based, contact mode: telephone, 1-2 times/week	40 weeks	Physical activity: program attendance	0.66
Galea et al., 2008, Australia	N=23, older adults 60+, currently have a clinical condition (post-operative after hip replacement), not specified	Community	Experimental-RCT	Exercise: Strength & flexibility training, 2x/week, 45 minutes	Group condition: center-based, standard face-face group, 2 times/week, group leader: health professional (physiotherapist), 11<br><br>Individual condition: home-based, contact mode: none, no contact	8 weeks	Physical activity: number of times exercised/week, Cadence (steps/min)<br><br>Functional: seconds needed to go up 4 steps, stair power, timed up and go test (secs), Distance (in meters) walked in 6 minutes.<br><br>Psychosocial: Quality of life	0.77

Ada et al., 2003, Australia	N=29, older adults 60+, currently have a clinical condition (after stroke), Previously sedentary or insufficiently active	Community	Observational - longitudinal	Exercise: Aerobic (Walking on treadmill), 3x/week, 30 minutes	Group condition: center-based, standard face-face group, 3 times/week, group leader: health professional (qualified physical therapist), group size: in dyads but in group setting<br><br>Individual condition: Home-based, Virtual-SMS or telephone contact, n contacts: Non-regular, 1-3 initial contacts then no contact	4 weeks	Physical activity: number of sessions attended, counts of activity (e.g., accelerometer epochs), cadence (steps/min)<br><br>Functional: distance walked in 6 mins, self-reported functional limitations.	0.84
Nyrop et al., 2014, USA	N=462, adults 18+, adults, currently have a clinical condition (joint pain/arthritis), not specified	Community	Observational-Longitudinal	Exercise: Aerobic (Walking on treadmill), 3x/week, 30 minutes	Group condition: delivery location not specified, standard face-face group, 3 times/week, qualified exercise instructor, group size not specified<br><br>Individual condition: home-based, no contact	6 weeks	Physical activity: Number walks/week, min/walk	0.36
Norton et al., 2015, Australia	N=871, adults 18+, healthy, previously sedentary, or insufficiently active	Community	Experimental-RCT	Exercise: Aerobic (Walking) 7x/week, 5000 steps/day	Group condition: center-based, true face-face group, 3 times/week (30 minutes), qualified exercise instructor, group size not specified.<br><br>Individual condition: home-based, no contact.	6 weeks	Functional: Handgrip strength, flexibility<br><br>Health: body weight, BMI, waist circumference, hip circumference, sum of skinfolds, systolic and diastolic blood pressure, total cholesterol, VO2max	0.84

Fuji et al., 2021, Japan	N=616, older adults 65+, health status not specified, mixed	Community	Observational – cross-sectional	Exercise: Mixed, no prescribed activity or duration, not applicable.	Group condition: mixed, not specified/other, no leader<br><br>Individual condition: no contact	N/A	Physical activity: total self-reported physical activity<br><br>Functional: Handgrip strength, sit-to-stand, 5 m walk test<br><br>Psychosocial: cognitive function (attention, memory, visuospatial, language, reasoning, overall cognition)	0.89
Praet et al., 2008, Netherlands	N=92, adults 18+, Currently have a clinical condition (Type 2 Diabetes), not specified	Healthcare	Experimental - RCT	Exercise: Moderate aerobic exercise, 3x/ week, 60 minutes	Group condition: not specified (health care setting), standard face-face group, 3 times/week, mixed (physical therapist and exercise trainer), group size: 15-25<br><br>Individual condition: home-based, not specified, not specified	52 weeks	Physical activity: adherence (not specified)<br><br>Health: VO2max, %- HbA1c, BMI, fasting blood glucose, insulin resistance, resting heart rate, systolic and diastolic blood pressure, total cholesterol, LDL, HDL, triglycerides<br><br>Psychosocial: health-related quality of life	0.79

Williams et al., 2021, Australia	N=50, older adults 60+, currently have a clinical condition (Multiple Sclerosis), not specified	Community	Experimental - RCT	Exercise: Mixed- Moderate aerobic and strength training, 2 times/ week, 60 minutes	Group condition: center-based, standard face-face group, 2 times/week, health professional (physiotherapist), 4-6<br><br>Individual condition: home-based, Virtual-SMS or Telephone, 1-3 times/month	8 weeks	Physical activity: adherence (% sessions completed)<br><br>Functional: Distance (m) walked in 6 minutes, Berg balance scale	0.91
Leach et al., 2019, USA	N=27, female adults 18+, Currently have clinical condition (Breast cancer- Stage 1 or II), previously sedentary or insufficiently active	Academic	Experimental - RCT	Exercise: Mixed- Moderate aerobic and strength training, 3x/ week, 60 minutes	Group condition: center-based, true face-face group, 2x times/week, research assistant, group size: 3-10<br><br>Individual condition: center-based, center based-face-face contact, 2 x/week	8 weeks	Physical activity: Walking time (MET-hours/week), Moderate (MET-hours/week)-MET-hours/week, Vigorous Physical Activity-MET-hours/week, MET-hours/week (all IPAQ)<br><br>Functional: Chest press, leg press, arm curl, time on treadmill, Sit to Stand test<br><br>Health: VO2max<br><br>Psychosocial: Quality of life	0.70

Cyarto et al., 2008, USA 1	N=167, older adults 65+, healthy, previously sedentary or insufficiently active	Community	Quasi-experimental wo/control	<u>Exercise:</u> Mixed-moderate balance, strength and aerobic training, 2x/ week, 30 minutes	<u>Group condition</u>: Centre-based (Retirement villages), true face-face group, 2 times/week, qualified exercise instructor, no of contacts not specified. group size not specified.<br><br><u>Individual condition</u>: Home-based, mixed- personal and telephone, non-regular contact (1-3 times/month)	20 weeks	<u>Physical activity</u>: Adherence (% sessions attended)<br><br><u>Functional</u>: Balance, timed-up-and-go	0.82
Martel et al., 2018, Canada	N=48, older adults 65+, healthy, previously sedentary or insufficiently active	Community	Experimental - RCT	<u>Exercise:</u> Mixed-moderate aerobic, strength and flexibility training, 2x/ week, 55 minutes	<u>Group condition</u>: center-based, standard face-face group, 2 times/week, mixed (health professional and qualified exercise instructor), group size: 6-8<br><br><u>Individual condition</u>: home-based, face-face contact, 1-2 times/week	12 weeks	<u>Physical activity</u>: Adherence (number of sessions completed), attendance (mean % of completed sessions)<br><br><u>Functional</u>: walking speed, physical functioning (SF-36), sit-to-stand test, timed-up-and-go test, one leg balance, handgrip<br><br><u>Health</u>: BMI<br><br><u>Psychosocial</u>: Cognitive impairment (MoCA)	0.66
Jansen et al., 2021, Germany	N=309, older adults 70+, healthy but at risk of falling, previously sedentary or	Community	Experimental - RCT	<u>Exercise:</u> Mixed-Moderate aerobic, strength and flexibility training, 3x/ week, Individual: 1-1.5	<u>Group condition</u>: center-based, standard face-face group, 2 times/month, health professional, group size: 8-12	11 weeks	<u>Physical activity</u>: steps/day (accelerometer), adherence (% sessions attended).	0.86

	insufficiently active.			hours, Group 2 – 2.5 hours.	Individual condition: home-based, face-face contact, 1-3 times/month.		Functional: life function and disability, sit-to-stand, balance, gait performance.<br><br>Psychosocial: fear of falling, balance confidence.	
Nerz et al, 2022, Germany	N=309, older adults 70+, health status not specified, previously sedentary or insufficiently active	Community	Experimental - RCT	Exercise: Mixed-Balance and strength training, not specified/no prescription.	Group condition: center based, standard face-face group, 2 times/month, qualified exercise instructor, group size: 8-12<br><br>Individual condition: home-based, face-face contact, no of contacts not specified	54 weeks	Physical activity: steps/day (accelerometer)<br><br>Functional: sit-to-stand, balance	0.67
Wu et al, 2010, USA	N=64, older adults 65+, healthy but at risk of falling, not specified	Community	Experimental - RCT	Exercise:<br><br>Tai Chi and Stretching, 3 times/week, 60 minutes	Group condition: online, standard online group, 3 times/week, qualified exercise instructor (Thai Chi instructor), group size not specified.<br><br>Individual condition: home-based, online, no contact, no contact (watched instructor video but no live contact)	15 weeks	Physical activity: number of minutes exercised, adherence (% sessions attended)	0.71
Donat, 2007, Turkey	N=42, older adults 65+, healthy, previously sedentary or insufficiently active	Healthcare	Experimental - RCT	Exercise: Balance, strengthening and stretching, 45-50 minutes, 3 times/week,	Group condition: healthcare (nursing home), standard face-face group, 3 times/week, health professional (physiotherapist), not specified	8 weeks	Physical activity: group-based: attendance (recorded by physiotherapist); home-based: Number of exercises completed (self-reported)	0.66

					Individual condition: home-based, face-face contact, health professional (physiotherapist), 1 times/month			
Cox et al., 2003, Australia	N=126, female adults, 18+, healthy, previously sedentary or insufficiently active	Community	Experimental-RCT	Exercise: Aerobics, 30 minutes, 3 times/week	Group condition: center-based, standard face-face group, 3 times/week, qualified exercise instructor, not specified. Individual condition: home-based, no contact (unsupervised).	24 weeks	Physical activity: Adherence (% participants retained), drop-out, % sessions completed, % exercises completed	0.87
Richardson et al., 2010, USA	N=324, adults 18+, clinical condition (overweight, type 2 diabetes, or coronary heart disease), previously sedentary or insufficiently active	Online	Experimental RCT	Exercise: Aerobic (Walking), not, specified, not specified	Group condition: Online, online community with interactive component, no scheduled time, no leader, group size not specified. Individual condition: Online, online message/email, not specified.	16 weeks	Physical activity: Steps/day, adherence (program completion)	0.77
Opdenacker et al., 2008, Belgium	N=186, older adults 60+, healthy, previously sedentary or insufficiently active	Community	Experimental RCT	Exercise: Aerobic, strength, flexibility, and balance training, 60-90 minutes, 3 times/week	Group condition: center-based, true face-face group, face-face, 3 times/week, qualified exercise instructor, 10 Individual condition: Home-based, virtual - SMS or telephone contact, 2 times/month	44 weeks	Physical activity: leisure-time, active transportation, household/garden, total (self-reported questionnaire), steps/day (pedometer), adherence (proportion)	0.74

Harris et al., 2016, United Kingdom	N=152, male adults 18+ (18-40), healthy, previously sedentary or insufficiently active	Community	Experimental RCT	Exercise:<br><br>Aerobic (Walking)<br><br>not, specified, not specified	Group condition: Online, online community with interactive component (group feedback), no leader, not specified<br><br>Individual condition: Home-based, only online feedback (no contact).	6 weeks	Physical activity: daily step count (app)	0.75
Malagoni et al., 2016, Italy	N=12, adults 18+, currently have a clinical condition (post-stroke survivors), not specified	Community	Experimental RCT	Exercise: Aerobic (Walking) (indoors), 20 minutes, 6 days/week<br><br>Group based condition additionally had 40 minutes of balance, strengthening and flexibility exercise.	Group condition: center-based,, standard face-face group, 3 times/week, health professional (physiotherapist), not specified.<br><br>Individual condition: Home-based, virtual – SMS or telephone contact, not specified.	10 weeks	Physical activity: Adherence (% sessions attended)<br><br>Functional: 6-min walk test, timed-up-and-go, stair climbing, physical functioning (SF-36)	0.79
Timonen et al., 2002, Finnland	N=68, older female adults, 75+, currently have a clinical condition (mobility impaired, frail), not specified	Healthcare	Experimental RCT	Exercise:<br><br>Functional and strength, 90 minutes, 2 times/week	Group condition: healthcare center-based (geriatric ward of hospital), true face-face group, 2 times/week, health professional (physiotherapist), 3-8<br><br>Individual condition: center-based, face-face, no contact (only one initial contacts)	10 weeks	Physical activity:<br><br>Adherence: % of sessions attended<br><br>Psychosocial: Emotional functioning (Self-Rating Depression Scale)	0.68

Baez et al., 2017, Italy	N=40, older adults 60+, healthy, not specified	Online	Experimental RCT	Exercise: Moderate Aerobic, Strength Training, & Flexibility, 30 minutes, 2 times /week,	Group condition: Online, true online group, 2 times/week, qualified exercise instructor, not specified<br><br>Individual condition: Home-based, online message/email, not specified	30 weeks	Physical activity: Adherence (% program adherence), % of exercise videos watched, and exercises completed	0.88
Mouton et al., 2015, Belgium	N=206, older adults, 50+, healthy, not specified	Community	Experimental RCT	Exercise:<br><br>Aerobic, strength and flexibility, not specified, 1 time /week,	Group condition: Centre-based, standard face-face group, 1 time/week, health professional (trained physical educator), not specified.<br><br>Individual condition: Home-based, virtual: online message/email, 1 times/month	12 weeks	Physical activity: Retention (completed 3-month assessment)	0.80
King et al., 1991, USA	N=357, older adults 50-65, healthy, previously sedentary or insufficiently active	Community	Experimental RCT	Exercise:<br><br>High intensity Training, & Flexibility, 3 times/week, 60 minutes	Group condition: center-based, standard face-face group, 3 times/week, qualified exercise instructor, not specified<br><br>Individual condition: Home-based, virtual: SMS or telephone contact, 1-3 times/month	12 weeks	Physical activity: Adherence (% sessions attended)	0.86

Helbostadt et al., 2004, Norway	N=77, older adults 75+, healthy, previously sedentary or insufficiently active	Community	Experimental RCT	Exercise:<br><br>Functional and strengthening training, 2 times/week, 60 minutes	Group condition: Centre-based, standard face-face group, 2 times/week, health professional (physiotherapist), 5-8<br><br>Individual condition: Home-based, personal contact, 1-3 times/month	12 weeks	Physical activity: Adherence (% sessions completed), number of days walked, walking time (duration)<br><br>Functional: Fast gait walking speed, preferred walking speed, physical functioning (SF-36)<br><br>Health: health-related quality of life (bodily pain), general health (self-report)<br><br>Psychosocial: vitality, social function (SF-36), emotional and mental functioning (SF-12)	0.85
Bieler et al., 2017, Denmark	N=152, older adults 60+, currently have clinical condition (hip osteoarthritis), previously sedentary or insufficiently active	Community	Experimental RCT	Exercise:<br><br>Mixed- Aerobic and Functional training, 3 times/week, 60 minutes	Group condition: Outdoor, standard face-face group, 3 times/week, health professional, (qualified physical therapist), not specified<br><br>Individual condition: Home-based, personal contact, irregular:1 initial contact then no contact	16 weeks	Physical activity: Adherence (% sessions completed)	0.91

Christle et al., 2017, Germany	N=60, older adults 70+, clinical condition (heart disease), previously sedentary or insufficiently active	Healthcare	Experimental RCT	Exercise:<br><br>Mixed- functional and strength and flexibility, 1 times/week, 60 minutes	Group condition: healthcare center-based, standard face-face group, 1 times/week, qualified exercise instructor, 15-20<br><br>Individual condition: Centre-based, personal contact, 1-2 times/week	26 weeks	Physical activity: Self-reported physical activity (IPAQ; % in PA category)<br><br>Functional: Health-related quality of life (physical; SF-36), quality of life (physical)<br><br>Health: General health (SF-36)<br><br>Psychosocial: Health-related quality of life (psychosocial; SF-36), negative affect, positive affect, emotional health-related quality of life, quality of life (social, total)	0.80
Kritz et al., 2020, Australia	N=58, older adults 60+, healthy, previously sedentary or insufficiently active	Community	Observational - Longitudinal	Exercise:<br><br>Aerobic (Walking), 3 times/week, 20 minutes	Group condition: Mixed-virtual, true face-face group, 2 times/week, trained peer group exercise leader, 3-10<br><br>Individual condition: Home-based, virtual SMS or telephone contact, 2 times/month	12 weeks	Physical activity: Physical activity (PASE)<br><br>Functional: 6-minute walk test<br><br>Health: % muscle mass, waist circumference (cm), % body fat, Body Mass Index<br><br>Psychosocial: Walking self-efficacy, motivation for walking	0.90

King et al., 1995, USA	N=269, older adults 50-65+, healthy, previously sedentary or insufficiently active	Community	Experimental RCT	<u>Exercise:</u><br><br>Mixed: High intensity Training, & Flexibility, 3 times/week, 60 minutes	<u>Group condition</u>: Centre-based, standard face-face group, 3 times/week, qualified exercise instructor, not specified<br><br><u>Individual condition</u>: Home-based, virtual SMS or telephone contact, 2 times/month	12 weeks	<u>Physical activity</u>: Adherence (% sessions attended, % program adhered to)	0.86
Aamot et al., 2016, Norway	N=83, adults 18+, currently have a clinical condition (diagnosed with myocardial infarction or acute coronary syndrome), not specified	Community	Experimental RCT	<u>Exercise</u>: High intensity training, 16 minutes, 2x/week	<u>Group condition</u>: Hospital, standard face-face group, 2 times/week, health professional (physiotherapist), 10-15<br><br><u>Individual condition</u>: Home-based, personal contact, irregular: 1-3 initial contacts then no contact	12 weeks	<u>Functional</u>: Health-related quality of life (physical)<br><br><u>Health</u>: VO2max, peak heart rate, Body Mass Index, body fat %, muscle mass %, resting metabolic rate (kcal/day)<br><br><u>Psychosocial</u>: Health-related quality of life (emotion, social, global)	0.82
Freene et al., 2015, Australia	N=158, older adults 50-65+, healthy, previously sedentary or insufficiently active	Community	Quasi-experimental wo/control	<u>Exercise</u>: Mixed-aerobic and strength training, 1 times/week, 60 minutes	<u>Group condition</u>: Centre-based, standard face-face group, 1 times/week, health professional, not specified<br><br><u>Individual condition</u>: Home-based, virtual SMS or telephone contact, 1-3 times/month	26 weeks	<u>Physical activity</u>: Moderate-Vigorous Physical Activity (ACTi Graph)<br><br><u>Functional</u>: 2-minute step test<br><br><u>Health</u>: Body Mass Index, waist-hip ratio, waist circumference, blood pressure, physical health (self-report; SF-12)	0.75

							Psychosocial: Mental health composite score (SF-12)	
Zhang et al., 2019, USA	N=91, female adults 18+, healthy, not specified	Community Online	Experimental RCT	Exercise: Mixed-aerobic and strength training, no prescription (self-monitored).	Group condition: Online, online community with interactive component (group feedback), no leader<br><br>Individual condition: Online, online message/email, not specified.	12 weeks	Physical activity: Steps/day, light PA (mins/day), MVPA (mins/day)<br><br>Functional: Number of push-ups<br><br>Health: Body Mass Index	0.89
Beauchamp et al., 2021, Canada	N=241, Older adults, 65+, healthy, sedentary or insufficiently active.	Online	Experimental RCT	Exercise: Mixed-strength, flexibility, balance, 3 times/week, 60 minutes	Group condition: Online,, true online group, 3 times/week, older peer group exercise leader, not specified.<br><br>Individual condition: online, online contact, 3 times/week, older peer-leader.	12 weeks	Physical Activity: Adherence (% attending at least 3 classes/week at Week 12)	0.79
Laham et al., 2019, Australia	N=45, older adults 60+, currently have a clinical condition (chronic obstructive pulmonary disease), previously sedentary or insufficiently active	Healthcare	Experimental RCT	Exercise: Mixed-Aerobic and strength,<br><br>6x/week,<br><br>30 minutes	Group condition: health care entre-based, standard face-face group, 3 times/week, health professional (physiotherapist), not specified.<br><br>Individual condition: Home-based, center based – personal contact, 1-2 times/week	8 weeks	Physical activity: Minutes of MVPA per day (accelerometer)	0.68

Moeller et al., 2020, Denmark	N=153, female adults 18+, currently have clinical condition (breast cancer), previously sedentary or insufficiently active	Healthcare	Experimental RCT	Exercise: Mixed-aerobic biking, resistance, circuit. 4x/week, 60 minutes, 30	Group condition: health care hospital-based, standard face-face group, 3 times/week,<br><br>qualified exercise instructor, 10<br><br>Individual condition: Home-based, center based – personal contact, 3 times/week	12 weeks	Physical activity: Adherence (% completing assessments)<br><br>Functional: Knee extensions, lateral pull, leg press, context peak left and right<br><br>Health: Body weight, fat mass, bone mass, lean body mass, bone mineral density, dyspnea, pain, fatigue<br><br>Psychosocial: Anxiety, depression	0.95
Iliffe et al., 2015, USA	N=1256, older adults 65+, healthy, previously active	Community	Experimental RCT	Exercise:<br><br>Moderate Aerobic, Strength Training, & Flexibility, 3x/week, 30 minutes	Group condition: center-based, standard face-face group, 3 times/week, research assistant, 15<br><br>Individual condition: Home-based, virtual SMS or telephone, 1-3 times/month	24 weeks	Physical activity: % participants with > 150 minutes MVPA/week, physical activity (Physical Activity Scale for the Elderly), FITT (phone)<br><br>Functional: Physical functioning (SF-36), balance, falls risk<br><br>Psychosocial: Social functioning, quality of life, exercise outcome expectations	0.86

Cyarto et al., 2008, Australia[1]	N=167, older adults 65+, healthy, previously sedentary or insufficiently active	Community	Quasi-experimental wo/control	Exercise:<br><br>Functional Balance and Strength. 2x/week, 60 minutes	Group condition: center-based, standard face-face group, 2 times/week, qualified exercise instructor, not specified.<br><br>Individual condition: Home-based, mixed- personal and telephone, non-regular contact (1-3 times/month)	20 weeks	Physical activity: % sessions attended<br><br>Functional: 30 second sit to stand test, 30 second arm curl test, chair sit and reach, back scratch test, timed-up-and-go test, 2-min step test.	0.95
Cyarto et al., 2006, Australia	N=119, older adults, 65+ (65-96), healthy, not specified	Community	Quasi-experimental wo/control	Exercise:<br><br>Functional Balance and Strength. 2x/week, 60 minutes	Group condition: Centre-based (Retirement villages), true face-face group, 2 times/week, qualified exercise instructor, 10<br><br>Individual condition: Home-based, telephone, 1-2 times/week	20 weeks	Physical activity: Adherence (mean proportion of sessions completed), compliance, % sessions attended	0.80
Tsekoura et al., 2018, Greece	N=54, older adults 65+, currently have a clinical condition (sarcopenia) previously sedentary or insufficiently active	Community	Experimental RCT	Exercise:<br><br>Mild Aerobic, Strength Training, & Flexibility, 3x/week, 30-35 minutes	Group condition: Centre-based, standard face-face group, 2 times/week, health professional, not specified<br><br>Individual condition: Home-based, personal contact, 1-3 times/month	12 weeks	Physical activity: Adherence (% sessions attended)<br><br>Functional: Timed-Up-And-Go, 4 m walk speed, gait speed, chair stand test, handgrip strength, knee extension and flexion<br><br>Health: Body Mass Index, skeletal muscle mass, fat free mass, calf circumference<br><br>Psychosocial: Quality of life	0.79

Yang et al., 2017, China	N=36, Adults 18+, currently have a clinical condition (diagnosed with mild to moderate Parkinson disease), previously sedentary or insufficiently active	Community	Experimental RCT	Exercise:<br><br>Tai chi, 7x/week, 40-45 minutes	Group condition: Hospital-based, standard face-face group, 7x times/week, qualified exercise instructor (Tai Chi instructor), 6-7<br><br>Individual condition: Hospital-based, personal contact, 3 times//week.	13 weeks	Physical activity: Adherence (% sessions attended)<br><br>Health: Sleep quality<br><br>Psychosocial: Depression, cognitive impairment	0.82
Akinci et al., 2018, Turkey	N=65, Adults 18+, currently have a clinical condition (Type 2 Diabetes), not specified	Community	Experimental RCT	Exercise: Aerobic (Walking), 3x/week, 60 minutes	Group condition: Not specified, standard face-face group, 3 times/week, not specified, health professional, not specified.<br><br>Individual condition: Home-based, virtual – online message/email, not specified	8 weeks	Physical activity: steps/day (pedometer)<br><br>Functional: 6-minute walk test<br><br>Health: HbA1C (%), fasting blood glucose, HDL, LDL, triglycerides, total cholesterol, Body Mass Index, waist circumference, hip circumference<br><br>Psychosocial: Quality of life	0.89
Brosseau et al., 2012, Canada	N=222, Adults 18+, currently have a clinical condition, (mild to moderate knee osteoarthritis), previously sedentary or	Community	Experimental RCT	Exercise:<br><br>Mild aerobic training<br><br>3x/week, 65 minutes	Group condition: outdoor, true face-face group, 2 times/week, qualified exercise instructor, not specified.<br><br>Individual condition: center-based, personal contact, non-regular: 1-3 initial contacts, then no contact.	20 weeks	Physical activity: Adherence (% sessions attended).<br><br>Psychosocial: Coping with symptoms, confidence about doing things	0.80

	insufficiently active.							
Kyrdalen et al., 2014, Norway	N=125, older adults 75+, healthy, previously sedentary or insufficiently active	Community	Experimental RCT	Exercise:<br><br>Mixed- functional and strength training and flexibility, 3 times/week, 60 minutes	Group condition: center-based, standard face-face group, 2 times/week, health professional, 4-8<br><br>Individual condition: Home-based, mixed personal and telephone, 1-3 times/month	12 weeks	Physical activity: Mean number of outdoor walks during intervention, adherence (% sessions attended)<br><br>Functional: Berg balance scale, Timed-Up-And-Go, sit-to-stand, physical functioning (SF-36)<br><br>Psychosocial: Mental health (SF-36)	0.88
Ramadi et al., 2015, Canada	N=3488, Adults 18+, currently have a clinical condition (diagnosed with coronary artery disease), previously sedentary or insufficiently active.	Community	Observational - Longitudinal	Exercise: Aerobic moderate-vigorous intensity exercise (45 to 85% of HRR and 12-14 RPE), 3-5x/week, 20-60 minutes.	Group condition: Centre-based, standard face-face group, 2-3 times/week, supervised (not specified by whom), not specified<br><br>Individual condition: Home-based, virtual – SMS or telephone, minimum of 1 session (unspecified).	12 weeks	Physical activity: Exercise sessions per week (self-reported), minutes per week<br><br>Functional: Exercise capacity (treadmill using Bruce or modified Bruce protocol).<br><br>Health: HR recovery, Body Mass Index, waist circumference, HDL, LDL,	0.85

							total cholesterol, triglycerides, fasting blood glucose.	
Thøgersen-Ntoumani et al., 2019, Australia	N=116, older adults 60+, healthy, previously sedentary or insufficiently active	Community	Quasi-experimental wo/control	Exercise:<br><br>Aerobic (Walking), 3 times/week, 20 minutes	Group condition: retirement village, true face-face group, 2 times/week, trained peer group exercise leader, 3-10<br><br>Individual condition: Home-based, virtual SMS or telephone contact, 2 times/month	16 weeks	Physical activity: Steps/day, stepping time, light physical activity (mins/day), moderate-vigorous physical activity (mins/day), sitting (mins/day), standing (mins/day) (accelerometer)<br><br>Functional: Physical health (SF-12), physical fitness (Dartmouth COOP charts)<br><br>Health: Change in health, pain<br><br>Psychosocial: Subjective vitality, mental health, emotional functioning, social role functioning (SF-12), social support, quality of life (Dartmouth COOP charts), anxiety, depression (HADS), loneliness	0.88

Seo et al., 2020, Korea	N=79, Adults 18+, healthy, previously sedentary or insufficiently active	Academic	Experimental RCT	Exercise:<br><br>Aerobic (Walking), not specified, 60 minutes/day	Group condition: Online, online community with interactive component, no scheduled time, no leader, not specified<br><br>Individual condition: Online, online, not specified, no contact, (only initial contact for distributing the trackers)	8 weeks	Physical activity: Steps/day<br><br>Health: Body weight, Body Mass Index, waist circumference, body fat, systolic and diastolic blood pressure<br><br>Psychosocial: Exercise motivation, perceived self-efficacy	0.67
Caserta et al., 1998, USA	N=146, older female, adults 60+, currently have a clinical condition (obesity), previously sedentary or insufficiently active	Community	Experimental RCT	Exercise: Aerobic moderate-vigorous intensity exercise (45 to 85% of HRR and 12-14 RPE), 3x/week, 60 minutes	Group condition: Center-based, standard face-face group, 3 times/week, health professional, not specified<br><br>Individual condition: Home-based, personal contact, 1-2 times/week	16 weeks	Physical activity: 7-day physical activity recall	0.75
King et al., 2002, USA	N=155, older adults 60+, healthy, previously sedentary or insufficiently active	Community	Experimental RCT	Exercise: Mixed-aerobic and strength training, 4x/week, 60 minutes	Group condition: Centre-based, standard face-face group, 3 times/week, health professional, 8-10<br><br>Individual condition: Home-based, personal contact, 1-3 times/month	56 weeks	Physical activity: Adherence (% sessions attended)<br><br>Functional: MacArthur Test, sit-to-stand test, standing balance, physical performance test, 6-minute walk test, 8-minute fast gait, 8 m usual gait, balance	0.93

							confidence, physical functioning (SF-36)<br><br><u>Psychosocial</u>: Mental health	
Ching et al., 1999, Taiwan	N=20, Adults 18+, healthy, previously sedentary or insufficiently active	Community	Observational - Longitudinal	<u>Exercise:</u><br><br>Mixed- Thai Chi (group) Aerobic (Walking) (Individual), Mixed- 3x/week- Individual, 7x/week group, 50 minutes	<u>Group condition</u>: Centre-based, standard face-face group, 3 times /week, qualified exercise instructor (Tai Chi instructor), 12<br><br><u>Individual condition</u>: Home-based, not specified, 3 times/week	52 weeks	<u>Physical activity</u>: Adherence (% sessions attended)<br><br><u>Functional</u>: Power work rate<br><br><u>Health</u>: VO2max, peak heart rate, peak O2 pulse, Ve peak, VR peak, peak heart rate, minute ventilation Ve	0.59
Owen et al., 1987, Australia	N=156, Adults 18+, healthy, previously sedentary or insufficiently active	Community	Experimental-RCT	<u>Exercise:</u><br><br>Moderate aerobic exercise, 2x/week, 60 minutes	<u>Group condition:</u><br><br>Centre-based, standard face-face group, 2 times/week, qualified exercise instructor, not specified<br><br><u>Individual condition:</u><br><br>Home-based. Virtual phone, not specified/once	12 weeks	<u>Physical activity</u>: Physical Activity (% meeting ACSM levels)	0.56

Granet at al., 2023, Canada	N=83, older adults 60+, healthy, not specified	Online	Experimental RCT	Exercise:<br><br>Mixed- Aerobic, strength training and flexibility, 3x/week, 55 minutes	Group condition: Online, standard online group, 3 times/week, health professional, not specified<br><br>Individual condition:<br><br>Online, not specified	12 weeks	Physical activity:<br><br>Adherence (n remaining in the intervention), attendance (% sessions completed)<br><br>Functional:<br><br>Balance (unimodal balance), fast gait walking speed, sit to stand 5rep, 10 rep, Muscle Power, 30 sec chair test, cardiovascular fitness- max aerobic power<br><br>Health: Body Mass Index	0.73
Langeard et al., 2022, France	N=43, older adults, aged 70-80, healthy, previously sedentary or insufficiently active	Community	Experimental RCT	Exercise: Mixed- Aerobic and strength training, 2x/week, 60 minutes	Group condition: Online, standard online group, 2 times/week, health professional, 4<br><br>Individual condition: Centre-based – personal contact, 2 times/week	13 weeks	Physical activity: Adherence (% sessions completed, number of sessions completed)<br><br>Functional: Handgrip strength, muscle function<br><br>Health: Cardio-respiratory fitness, heart rate, body weight (kg), body fat (%), muscle mass (%)	0.80
Middlewerd et al., 2022, Netherlands	N=77, Adults 18+, healthy, previously sedentary or	Academic	Quasi-experimental wo/control	Exercise:<br><br>Not specified (presuming it is Aerobic, walking	Group condition: Online, online community with interactive (feedback) component 1 time/week, no leader, 6	12 weeks	Physical activity: MVPA, steps/day (accelerometer), steps/day (Fitbit)	0.72

	insufficiently active			as steps are measured), prescription not specified (individually tailored).	Individual condition: Online, no contact, not specified		Psychosocial: Self-efficacy, outcome expectations, social norms, intentions, barriers, self-regulation skills	
King, 1997, USA	N=269, Adults 18+, healthy, previously sedentary or insufficiently active	Community	Experimental RCT	Exercise:<br><br>Aerobic (high and low intensity) training, 3 times/ week, 60 minutes	Group condition: Centre-based, standard face-face group, 3 times/week, qualified exercise instructor, not specified<br><br>Individual condition: Home-based, virtual SMS or telephone contact, less than 1 time/month	112 weeks	Physical activity: Adherence (% sessions attended), self-reported total MVPA/week	0.78
Looyestyn et al., 2018, Australia	N=89, Adults 18+, healthy, previously sedentary or insufficiently active	Online	Experimental RCT	Exercise: mixed-aerobic, running/interval training, not specified, 30 minutes	Group condition: Online, online community group with interactive component, 7 days/week (online motivational posts), qualified exercise instructor, 41<br><br>Individual condition: Online, no contact, self-directed	8 weeks	Physical activity: Adherence<br><br>Health: Cardio-respiratory fitness<br><br>Psychosocial: Self-efficacy, exercise attitudes, social support	0.68
Nikitina et al., 2018, Italy	N=44, older adults 59+ (59-83), healthy, previously sedentary or insufficiently active	Online	Experimental RCT	Exercise:<br><br>strength and balance training, 2x/week, 30-40 minutes	Group condition: Online, true online group (social interaction features and classes), 2 times/week, qualified exercise instructor (coach- medical doctor), 20	8 weeks	Physical activity: Adherence (number of sessions attended)	0.54

					<u>Individual condition</u>: Online, online message/email, 2 times/week			
Shabbir et al., 2024, Canada	N=38, older male adults 60+, currently have a clinical condition (diagnosed with prostate cancer of any stage, starting or continuing androgen deprivation therapy for at least six months), previously sedentary or insufficiently active	Community	Experimental RCT	<u>Exercise:</u> Mixed- a combined aerobic and resistance, flexibility exercise session, 4–5x/week, 60 minutes.	<u>Group condition</u>: center based, standard face-face group, 3 times/week, qualified exercise instructor, not specified<br><br><u>Individual condition</u>: Home-based, face-face, not specified.	24 weeks	<u>Physical activity</u>: Sedentary minutes/day, MVPA minutes/day (accelerometer)<br><br><u>Function</u>: Sit-to-stand, handgrip strength, 6-minute walk test<br><br><u>Health</u>: Fatigue, fasting blood glucose, HbA1c, HDL, Hemoglobin, LDL, PSA, testosterone, total cholesterol, triglycerides, waist circumference, waist: hip ratio, fat free mass, body fat, BMI<br><br><u>Psychosocial:</u> Quality of life	0.83
Arevalo et al., 2023, USA	N=30, Adults 18+, healthy, previously sedentary or insufficiently active	Community	Quasi-experimental wo/control	<u>Exercise:</u><br><br>Mixed- flexibility, cardiovascular, and strength exercises, 3x/week, 60 minutes	<u>Group condition</u>: Centre-based, standard face-face group, 2 times/week, qualified exercise instructor, not specified<br><br><u>Individual condition</u>: Home-based, face-face, 1 time/month	12 weeks	<u>Physical activity</u>: Adherence (% sessions attended)	0.72

Krumov et al., 2022, Bulgaria	N=130, Older adults 65+ (65-80), healthy, previously sedentary or insufficiently active	Healthcare	Experimental RCT	Exercise: Mixed-strength and balance exercises, not specified, not specified.	Group condition: Centre-based, standard face-face group, 3 times/week, health professional, 5-8<br><br>Individual condition: Centre-based, personal contact, not specified	12 weeks	Physical activity: Total physical activity (self-reported, PASE)<br><br>Function: 6-minute walk test, physical functioning (SF-36)<br><br>Health: General health, bodily pain<br><br>Psychosocial: Vitality, social functioning, emotional functioning, overall mental health	0.78
Karmakar et al., 2022, Hong Kong	N=107, Older adults 60+, healthy, previously sedentary or insufficiently active	Community	Experimental RCT	Exercise:<br><br>Aerobic (Walking), 3x/week, 50-70 minutes	Group condition: Centre-based, standard face-face group, 3 times/week, health professional, not specified<br><br>Individual condition: Centre-based, personal contact, 3 times/week	15 weeks	Function: Physical performance, physical functioning (SF-36)<br><br>Health: General health, Body Mass Index, resting heart rate, systolic and diastolic blood pressure, bodily pain<br><br>Psychosocial: Cognitive impairment, vitality, social functioning, emotional functioning, overall mental health, physical activity enjoyment	0.84

Wan et al., 2017, USA	N=109, Older adults 60+, currently have a clinical condition (Had a diagnosis of COPD and received medical clearance to participate), previously sedentary or insufficiently active	Healthcare	Experimental RCT	Exercise:<br><br>Aerobic (Walking), not specified, not specified.	Group condition: Online, online community with interactive component, no scheduled time, no leader, not specified<br><br>Individual condition: Online, telephone, not specified	13 weeks	Physical activity: Steps/day (pedometer), adherence (% pedometer wear days)	0.84
King et al., 1993, USA	N=151, Adults 18+, healthy, previously sedentary or insufficiently active	Community	Experimental RCT	Exercise:<br><br>Mixed: High intensity Training, & Flexibility exercises, 3x/week, 60 minutes.	Group condition: Centre-based, standard face-face group, 3 times/week, qualified exercise instructor, not specified<br><br>Individual condition: Home-based, virtual SMS or telephone contact, 1-3 times/month	12 weeks	Physical activity: Adherence (% program adhered to)<br><br>Psychosocial: Perceived stress, anxiety, depressed mood	0.86
Desbiens et al., 2017, Canada	N=26, Older female, 60+, currently have a clinical condition (diagnosed with stage 1-3 breast cancer), mixed	Healthcare	Experimental RCT	Exercise:<br><br>Mixed: Aerobic, strength and flexibility, 2 times/week, 50 minutes	Group condition: Not specified, standard face-face group, 2 times/week, health professional (kinesiologist), not specified<br><br>Individual condition: Home-based, no contact, no contact (video asked to do it 2 times/week).	12 weeks	Physical activity: Completion (% completing the programme)	0.73

Watanabe et al., 2020, Japan	N=517, older adults 60+, healthy, previously sedentary or insufficiently active	Community	Experimental RCT	Exercise:<br><br>Mixed- aerobic PA and resistance<br><br>1x/week, 90 minutes.	Group condition: Centre-based, standard face-face group, 1 times/week, health professional, 6-30<br><br>Individual condition: Home-based, mixed (email and personal contact), 1 times/month	12 weeks	Physical activity: Steps/day, MET hours/week (accelerometer)<br><br>Functional: Handgrip strength, knee extension strength, walking speed (normal and maximal), timed-up-and-go, sit-to-stand, functional reach, chair stepping, vertical jump<br><br>Health: Anterior thigh muscle thickness	0.80
Holland et al., 2017, Australia	N=166, older adults 60+, currently have a clinical condition (COPD), previously sedentary or insufficiently active	Community	Experimental RCT	Exercise:<br><br>Mixed- aerobic and resistance.<br><br>2x/week, 30 minutes	Group condition: Centre-based, standard face-face group, 2 times/week, health professional, not specified<br><br>Individual condition: Home-based, mixed (personal and telephone contact), 1times/week	8 weeks	Physical activity: Adherence (% sessions attended)	0.90

Emilia et al., 2018, Malaysia	N=62, Adults 18+ (35-45), healthy, previously sedentary or insufficiently active	Healthcare	Experimental RCT	Exercise: Aerobic (Walking), 10,000 steps/day.	Group condition: healthcare entre-based (primary care clinic), standard face-face group, 1 times/month, research assistant, 10-15<br><br>Individual condition: Home-based, no contact, no contact (only initial introductory session).	12 weeks	Physical activity: Steps/day	0.66

Note: 1= Cyarto et al. (2008): These studies utilized the same sample but were counted as separate studies in this analysis because their results were published in separate articles and addressed different outcomes Detailed information on how each outcome category was defined and coded is available on the project's OSF page: https://doi.org/10.17605/OSF.IO/XT2G4

j
