# Supplementary material for: Individual versus group-based interventions: a systematic review and meta-analysis of physical activity, functional, psychosocial and health outcomes
Source: Nat Hum Behav. 2026 Apr 15;10(6):1109–21. doi: 10.1038/s41562-026-02429-0 (PMC13290480; doi:10.1038/s41562-026-02429-0)
Supplement: Supplementary file 3 — Peer Review File [file 41562_2026_2429_MOESM3_ESM.pdf]

Peer Review File

Individual vs. group-based interventions: A systematic review and meta-analysis of physical activity, functional, psychosocial, and health outcomes

Corresponding Author: Dr Marlene Kritz

Version 0:

Decision Letter:

16th April 2025

Dear Dr Kritz,

Thank you once again for your manuscript, entitled "Solo or team? A meta-analysis of individual vs. group-based studies on physical activity, functional, psychosocial, and health outcomes.", and for your patience during the peer review process.

Your Article has now been evaluated by 3 referees. You will see from their comments copied below that, although they find your work of considerable potential interest, they have raised quite substantial concerns. In light of these comments, we cannot accept the manuscript for publication, but would be interested in considering a revised version if you are willing and able to fully address reviewer and editorial concerns.

We hope you will find the referees' comments useful as you decide how to proceed. If you wish to submit a substantially revised manuscript, please bear in mind that we will be reluctant to approach the referees again in the absence of major revisions. We are committed to providing a fair and constructive peer-review process. Do not hesitate to contact us if there are specific requests from the reviewers that you believe are technically impossible or unlikely to yield a meaningful outcome.

To guide the scope of the revisions, the editors discuss the referee reports in detail within the team, including with the chief editor, with a view to (1) identifying key priorities that should be addressed in revision and (2) overruling referee requests that are deemed beyond the scope of the current study. We hope that you will find the prioritised set of referee points to be useful when revising your study. Please do not hesitate to get in touch if you would like to discuss these issues further.

1. Reviewer 1 is concerned about the effects of heterogeneity on the findings. Please carefully address this concern and conduct robustness tests.

2. Our reviewers raise questions about some analytical choices such as the inclusion of digital interventions, the aggregation of experimental and observational studies, as well as the exclusion of conference proceedings, theses and dissertations and outliers. We ask that you provide a strong motivation for all analytical choices and conduct sensitivity analyses.

Finally, your revised manuscript must comply fully with our editorial policies and formatting requirements. Failure to do so will result in your manuscript being returned to you, which will delay its consideration. To assist you in this process, I have attached a checklist that lists all of our requirements. If you have any questions about any of our policies or formatting, please don't hesitate to contact me.

If you wish to submit a suitably revised manuscript, we would hope to receive it within 3 months. I would be grateful if you could contact us as soon as possible if you foresee difficulties with meeting this target resubmission date.

With your revision, please:

• Include a “Response to the editors and reviewers” document detailing, point-by-point, how you addressed each editor and referee comment. If no action was taken to address a point, you must provide a compelling argument. When formatting this document, please respond to each reviewer comment individually, including the full text of the reviewer comment verbatim followed by your response to the individual point. This response will be used by the editors to evaluate your revision and

sent back to the reviewers along with the revised manuscript.

• Highlight all changes made to your manuscript or provide us with a version that tracks changes.

Please use the link below to submit your revised manuscript and related files:

Link Redacted

Note: This URL links to your confidential home page and associated information about manuscripts you may have submitted, or that you are reviewing for us. If you wish to forward this email to co-authors, please delete the link to your homepage.

Thank you for the opportunity to review your work. Please do not hesitate to contact me if you have any questions or would like to discuss the required revisions further.

Sincerely,

[redacted]

[redacted]
Nature Human Behaviour

Reviewer expertise:

Reviewer #1: individual and group-based studies on physical activity ; functional, psychosocial, and health outcomes

Reviewer #2: individual and group-based studies on physical activity ; functional, psychosocial, and health outcomes

Reviewer #3: systematic reviews and meta-analyses

REVIEWER COMMENTS:

Reviewer #1 (Remarks to the Author):

Overall comments:
Thank you for the opportunity to review "Solo or team? A meta-analysis of individual vs. group-based studies on physical activity, functional, psychosocial, and health outcomes." This study aims to update and extend Burke et al.'s (2006) meta-analysis comparing group-based and individual physical activity interventions. The meta-analysis provides a valuable update with a large sample and advanced methods, aligning well with Nature Human Behaviour's focus on human behavior and social challenges.
The manuscript follows the necessary steps by adhering to PRISMA guidelines and conducting a comprehensive analysis to achieve the study's goals. It is clearly written, with a logical flow from methods to results and discussion. The detailed reporting of moderator analyses and sensitivity tests enhances transparency. Furthermore, the discussion effectively situates the findings within the existing literature, referencing both historical and contemporary studies.
However, based on the journal guidelines and review criteria, several critical issues undermine the manuscript's robustness and impact, requiring substantial explanations and appropriate revisions:
• The high heterogeneity remains poorly explained despite moderator analyses, weakening confidence in pooled effect sizes. Subgroup analyses (e.g., by population—healthy vs. clinical—or intervention duration) are needed to pinpoint variability sources and refine conclusions.
• Moderate GRADE ratings for most outcomes (low for psychological) stem from imprecision, bias risks (e.g., lack of blinding), and reliance on self-reports, casting doubt on the stability of the results. A sensitivity analysis excluding indirect psychological measures (e.g., general well-being) and focusing on specific constructs (e.g., loneliness) could strengthen validity.
• The shift to significance for functional outcomes only after outlier removal raises concerns about selective reporting. A detailed justification for exclusions (e.g., study-specific traits) and consistent reporting of results with and without outliers across all outcomes are essential.
• Moderator analyses lack explanatory power, and low study power (per funnel plots) risks missing true effects. Testing moderator interactions (e.g., group type × leader) and adding a post-hoc power calculation could help clarify these weaknesses.
• The discussion does not fully explore practical implications (e.g., scalability) or theoretical alignment (e.g., self-determination theory), limiting its reach. Providing concrete examples (e.g., policy applications) and deeper theoretical synthesis would enhance the manuscript's contribution to the field.

Specific Comments Following the Journal's Reviewer Guidelines
Study Rationale
The study presents a strong rationale for updating Burke et al.'s (2006) meta-analysis but lacks the precision needed to

convincingly justify its urgency and originality. The statement that “a multitude of relevant new studies” have emerged (line 101) is too vague, as it does not provide concrete examples or preliminary evidence (e.g., the number of studies published since 2006, notable shifts in effect sizes). Without such details, the argument for why an update is necessary feels assumed rather than empirically justified. The authors should consider strengthening this section with quantitative indicators, such as citation trends or methodological advancements in PA interventions, to reinforce the study’s relevance.

Additionally, while the inclusion of online groups and virtual reality (line 106) is a valuable expansion, the rationale does not clearly connect these modalities to the specific limitations of Burke et al.’s findings. A stronger statement is needed to explain why the rise of digital interventions necessitates reassessment, particularly in light of changes in exercise behavior post-COVID, increased accessibility through technology, or different motivational structures in virtual environments. The current study’s aim (line 116) is also broad and does not explicitly pose a specific research question or hypothesis. The authors should consider elaborating on whether the study seeks to determine if online groups outperform individual PA interventions post-COVID or whether digital interventions lead to higher adherence rates compared to in-person groups. Clarifying this would provide a sharper focus and strengthen the study’s positioning.

Key Results

The key findings—statistical equivalence for PA and health outcomes, alongside a significant advantage for group-based interventions in functional outcomes after outlier removal—are important but require greater clarity and stability. The shift to significance for functional outcomes only after excluding outliers (line 396) raises questions about the robustness of this finding, given that statistical significance was only achieved after outlier exclusion. Without a transparent justification for why these specific cases were removed, the reliability of this finding is weakened. The authors should consider providing study-specific details explaining whether exclusions were based on data errors, methodological inconsistencies, or extreme effect sizes.

Additionally, small, non-significant effect sizes for PA (line 312) and wide confidence intervals (e.g., [-.061, .233]) highlight potential imprecision, yet these concerns are not directly addressed. Reporting results both with and without outliers for all outcomes would enhance transparency and allow readers to assess the robustness of conclusions. Furthermore, the discussion on functional outcomes should explore why this specific domain showed a group advantage—was it due to social modeling, enhanced accountability, or leader feedback? The authors should consider providing a clearer mechanistic explanation, possibly drawing from social cognitive theory (e.g., observational learning, vicarious reinforcement), to strengthen the interpretation.

Validity

The validity of the conclusions is weakened by unresolved methodological concerns. The substantial heterogeneity remains despite the inclusion of moderators such as group leader type (line 327), suggesting that key explanatory factors remain unaccounted for. The moderate-to-low GRADE ratings (e.g., low for psychological outcomes, line 437) further highlight limitations in measurement precision, particularly due to reliance on self-reported data and the absence of blinding in many studies.

Furthermore, the dependence of functional outcome significance on outlier removal (line 331) introduces uncertainty into the conclusions. Addressing these concerns requires conducting additional subgroup analyses (e.g., stratified by population type—clinical vs. non-clinical—or intervention setting). A sensitivity analysis that excludes studies relying solely on self-reported psychological outcomes would help assess whether measurement bias affects results. Finally, the authors should explicitly acknowledge these methodological limitations to avoid overgeneralization.

Significance

While the study’s significance lies in updating Burke et al., the small effect sizes (line 312) and alignment with prior findings (line 457) make the contribution feel incremental rather than transformative. The discussion highlights how modern PA interventions include online groups (line 506), but the practical implications remain vague.

To enhance the study’s impact, the discussion should explicitly connect findings to real-world applications. For example, the equivalence of online and in-person PA groups could be leveraged to support scalable virtual PA programs in rural communities or among populations with mobility constraints. Additionally, if group-based PA improves functional outcomes, this insight could inform rehabilitation programs for older adults or clinical populations where functional ability is a primary concern. The authors should explicitly link results to pressing public health challenges, such as declining physical activity rates post-COVID or digital health interventions in underserved areas.

Data and Methodology

The methodology is rigorous but contains gaps that weaken data quality and comprehensiveness. The exclusion of dyadic interventions (line 132) is not sufficiently justified, as dyads also involve social influences that could be relevant to PA interventions. Either providing a stronger theoretical basis for excluding dyads or reconsidering their inclusion could enhance the study’s comprehensiveness.

Additionally, missing data from non-responsive authors (line 190) introduces potential selection bias, yet the manuscript does not specify how many studies were affected or whether imputation was considered. Reporting the proportion of missing data and assessing its potential impact on findings would increase transparency. The high heterogeneity (e.g., line 313) is acknowledged but not fully addressed (line 542), making it difficult to interpret pooled estimates. Conducting additional subgroup analyses, particularly by intervention type or delivery format (e.g., online vs. in-person), could help address this concern.

Analytical Approach

The analytical approach is methodologically sound but presents limitations in explanatory power. The three-level meta-analytic model and TOST equivalence testing are appropriate, but moderator analyses (e.g., group leader, line 324) fail to fully explain heterogeneity (line 327). This suggests that additional moderator interactions should be tested, such as whether the impact of group type depends on the leader's role or whether intervention duration influences results differently across settings. The low statistical power to detect small-to-moderate effects (line 352) raises concerns about false negatives, particularly for secondary outcomes. Conducting a post-hoc power analysis would clarify whether the study was adequately powered to detect meaningful effects.

Furthermore, the handling of outliers, particularly their impact on functional outcomes (line 396), remains insufficiently

justified. The authors should consider explaining the rationale behind outlier exclusion—whether due to data errors, methodological inconsistencies, or extreme effect sizes— to ensure replicability and reduce concerns about selective reporting.
Technical Density
The technical density of the manuscript may hinder accessibility for readers unfamiliar with meta-analytic techniques. Sections explaining TOST (line 247) and the three-level model (line 242) are particularly dense, which may make interpretation challenging for a broader audience.
To improve clarity, the manuscript could introduce a brief glossary or footnotes defining key statistical terms such as "TOST" and "Hedges' g" in more accessible language. The discussion (line 451) references theories like self-determination theory (line 477) but does not thoroughly integrate them into the interpretation of findings. The authors should clearly explain how these theories support or challenge the results—such as whether social relatedness influences group-based PA adherence. Additionally, practical implications should be made more tangible by providing specific examples, such as how virtual group-based PA programs could be implemented in workplace wellness initiatives or public health campaigns. These refinements would enhance the manuscript's readability and real-world applicability.

Reviewer #2 (Remarks to the Author):

This is a very useful and well conducted review, investigating a topic that has implications for resource allocation. The review is an update on one conducted previously, but appears warranted given the much larger number of studies available to provide greater confidence in findings. Particular strengths that provide confidence in the findings include the rigorous screening and data extraction process (incorporating but not reliant on AI), inclusion of online groups which had not been the case in the previous review, and greater availability of objectively measured PA of study outcomes. Overall the authors did an excellent job on reporting a complex set of analyses, and incorporation of multiple dimensions. I have only a couple of minor comments to add.

Introduction
- P5 Line 110 – minor point, but the suggestion that there is a lasting new normal of social distancing needs supporting. For many exercise facilities do not appear different from pre-pandemic arrangements, so evidence that this impression is wrong/limited is needed.

Methods
- It was not clear in the Methods what would be done with the data on study quality and risk of bias. For ROB, it's stated that evidence would be downgraded where needed, but not what would happen in the analysis for a downgraded study.
- It would be useful to clearly articulate what a priori moderators were planned to be included in the analyses (p10 line 236).

Reviewer #3 (Remarks to the Author):

Solo or team? A meta-analysis of individual vs. group-based studies on physical activity, functional, psychosocial, and health outcomes

The present paper reported a meta-analysis of 71 studies containing 523 effect sizes comparing individual and group-based physical activity on outcomes: PA levels, functional assessments, psychosocial outcomes, and health outcomes. With some exceptions, results generally showed that both types of physical activity tended to show similar outcomes, with effects generally small and non-significant.

Overall, there is a lot to like about this paper. I enjoyed the brevity, and the fact that it was really well written and easy to read. The meta-analysis is reported transparently, with much additional information accessible on the OSF. Moreover, results were pre-registered ahead of time on PROSPERO and the authors followed PRISMA protocols. The analyses seemed well justified and both competently and carefully done.

That said, I do have some points of feedback that I note below in case the authors want to provide further clarity about some issues:

1. On page 1 (first paragraph) it is noted that a substantial proportion of adults fail to reach the recommended guidelines for PA. It might be worth briefly mentioning what these are for people who are not in this field

2. On page 3 of the introduction, true groups are described. As someone who is not from the PA field, this still read as a bit unclear to me. It is currently described as a group where group dynamics principles such as team building are used to promote group cohesiveness, which to me doesn't really provide much clarity. I think it would help readers to provide an example here, as the definition to me doesn't really help make these two subtypes clearly distinct.

3. To some extent, the introduction appears to frame the COVID-19 pandemic and its associated measures as ongoing concerns. However, this perspective does not really align with the current global situation. Many countries have moved well beyond strict pandemic protocols, and practices like social distancing are no longer widely enforced or promoted. To enhance the paper's longevity and broader applicability, I would suggest shifting the focus of the introduction away from

specific COVID-19 impacts and focus it on more general issues where possible. This approach would not only make the paper more relevant to a wider range of scenarios but also prevent it from becoming quickly outdated as the world continues to move beyond COVID-19.

4. Unless it is a journal requirement, I would recommend that the authors consider placing the search strategy details before the eligibility criteria, as the former helps to give the latter a bit more context.

5. I found myself wondering why conference proceedings and theses/dissertations were excluded? I think a strong defense is needed here since these are not recommend practices and can create publication bias in the results.

6. A minor point, but technically web of science and proquest are not actual databases, but rather platforms used to search databases. I would recommend framing the search around the papers that describe best practices for literature searching, since the authors appeared to apply a lot of these best practices anyway. For instance, the following might help:

a. Harari, M. B., Parola, H. R., Hartwell, C. J., & Riegelman, A. (2020). Literature searches in systematic reviews and meta-analyses: A review, evaluation, and recommendations. Journal of Vocational Behavior, 118, 103377.

7. While I am open to AI in streamlining some of the more procedural aspects of meta-analysis, as the authors have done here with the Research Screener Software, I think it would provide more weight to their review if they provided some evidence backing up that this is a valid tool and tends to result in reliable decisions – is there any evidence that can back up this tool?

8. Related to the above, it may also be worthwhile including some interrater reliability statistics in the screening decisions. Perhaps there is an opportunity to compute this for the human/AI decision points to test the accuracy of the decisions?

9. A final point about screening is that I also found the section on title and abstract screening to be hard to follow, despite reading this paragraph multiple times. I would suggest revising to make things clearer and easier to follow.

10. I think a stronger defence could be provided about why both observational and experimental studies were aggregated together. Even though the effects were converted to a common metric, there is still the problem that these different study designs quantify different information. Thus, in my mind it only serves to make effect sizes difficult to interpret. Since there were only a handful of observational studies anyway, perhaps it would be a cleaner meta-analysis if only the experimental studies were included? In any case, a stronger defence is needed here.

11. From page 22, the following statement: “Factors such as the predominance of cross-sectional studies, impression, risk of bias, high heterogeneity, lack of control for confounding, heavy reliance on self-reports for some outcomes (e.g., psychological) and indirect measures for others (e.g., class attendance for PA levels), and potentially underpowered studies, indicated that there is substantial room for improvement.” was a bit confusing since most of the included studies in the meta-analysis were experimental, which seems at odds with this statement suggesting a predominance of cross-sectional studies. I would suggest revising for clarity.

12. Another limitation with the meta-analysis is that (from what I could tell) effects were not disattenuated for error in measurement. I don’t think this is a confound, since it would affect both individual PA and group-based PA equally, but it would still give a more pure assessment of the effects. Something to consider.

Overall, I enjoyed reading this paper and wish the authors the best in their continued work.

Version 1:

Decision Letter:

5th September 2025

Dear Dr Kritz,

Thank you once again for your revised manuscript, entitled "Solo or team? A meta-analysis of individual vs. group-based studies on physical activity, functional, psychosocial, and health outcomes.," and for your patience during the re-review process.

Your manuscript has now been evaluated by Reviewers 1 and 3 from the original round of review. All reviewer feedback is included at the end of this letter. Although the reviewers found your manuscript to have improved during revision, they also

raise some important outstanding concerns. We remain very interested in the possibility of publishing your study in Nature Human Behaviour, but would like to consider your response to these outstanding concerns in the form of a revised manuscript before we make a decision on publication.

1. Reviewer 3 has remaining concerns about the exclusion of unpublished literature. We share these concerns and ask that you include unpublished literature in your search and analysis.

2. Reviewer 1 has remaining concerns about the heterogeneity reporting and outlier handling and encourages you to expand the publication bias assessment. We ask that you address these concerns in full.

Finally, your revised manuscript must comply fully with our editorial policies and formatting requirements. Failure to do so will result in your manuscript being returned to you, which will delay its consideration. To assist you in this process, I have attached a checklist that lists all of our requirements. If you have any questions about any of our policies or formatting, please don't hesitate to contact me.

In sum, we invite you to revise your manuscript taking into account all reviewer and editor comments. We are committed to providing a fair and constructive peer-review process. Do not hesitate to contact us if there are specific requests from the reviewers that you believe are technically impossible or unlikely to yield a meaningful outcome.

We hope to receive your revised manuscript within 8-12 weeks. I would be grateful if you could contact us as soon as possible if you foresee difficulties with meeting this target resubmission date.

With your revision, please:

• Include a "Response to the editors and reviewers" document detailing, point-by-point, how you addressed each editor and referee comment. If no action was taken to address a point, you must provide a compelling argument. This response will be used by the editors and reviewers to evaluate your revision.

• Highlight all changes made to your manuscript or provide us with a version that tracks changes.

Please use the link below to submit your revised manuscript and related files:

Link Redacted

Note: This URL links to your confidential home page and associated information about manuscripts you may have submitted, or that you are reviewing for us. If you wish to forward this email to co-authors, please delete the link to your homepage.

We look forward to seeing the revised manuscript and thank you for the opportunity to review your work. Please do not hesitate to contact me if you have any questions or would like to discuss these revisions further.

Sincerely,

[redacted]

[redacted]
Nature Human Behaviour

REVIEWER COMMENTS:

Reviewer #1 (Remarks to the Author):

Overall Comments:
Thank you for the opportunity to review the revised version of "Manuscript #NATHUMBEHAV-25010440A: Solo or team? A meta-analysis of individual vs. group-based studies on physical activity, functional, psychosocial, and health outcomes." The revision represents a significant improvement in both scope and clarity. The rationale for updating Burke et al.'s (2006) meta-analysis has been strengthened, methodological details are more transparent, and the discussion more effectively integrates theoretical frameworks and practical applications. These enhancements have made the manuscript substantially stronger and more relevant to both research and practice. At the same time, several areas still require further refinement to meet the highest standards of methodological rigor and interpretive precision. The following comments are intended to guide these final revisions.

Specific Comments:
1. Heterogeneity reporting: The authors should consider expanding their reporting of heterogeneity by including variance components from the three-level model, 95% prediction intervals, and estimates of the proportion of variance explained by moderators (e.g., pseudo-R^2). Adding omnibus tests of moderator significance and interpreting these results clearly would

help readers better understand residual heterogeneity and the robustness of pooled estimates.
2. Outlier transparency: The authors should consider presenting side-by-side results with and without outliers across all outcome domains. Anchoring exclusion criteria in established influence diagnostics is helpful, but transparency would be further improved by including diagnostic plots or reproducible analytic details (e.g., code) in the Supplement. This would enhance replicability and reassure readers that conclusions are not contingent on analytic choices.
3. Equivalence testing: The authors should consider explicitly defining and justifying a smallest effect size of interest as the basis for equivalence bounds. Clear justification for these bounds, drawn from prior literature or applied relevance, would bring equivalence testing in line with best practice. It would also be useful to consistently report both 90% confidence intervals and 95% confidence intervals (for traditional inference) and clarify whether equivalence conclusions are robust across sensitivity analyses.
4. Publication Bias Diagnostics
The authors should consider extending their publication bias assessment beyond funnel plots and Egger’s test by applying at least one additional method (e.g., a selection model, p-curve, or p-uniform analysis) for the primary outcome domain. This would provide additional assurance about the robustness of conclusions, particularly in light of potential small-study effects.
5. Protocol transparency: The authors should consider referencing a publicly available protocol (e.g., PROSPERO, OSF). If no public registration exists, providing a dated version of the protocol in the Supplement would enhance transparency. Any deviations from the original plan should also be noted explicitly.
6. Theoretical and Practical Integration: The authors should consider further strengthening the discussion by more explicitly connecting their findings to cost-effectiveness and equity considerations. In particular, the equivalence of online and in-person formats has important implications for scalable and resource-efficient delivery, which could be highlighted more directly to inform public health and policy debates.
7. Limitations and Future Direction: The authors should consider expanding their limitations section to explicitly acknowledge the lack of adherence/retention data and the inconsistent reporting of intervention settings across primary studies. Highlighting these gaps would underscore important priorities for improving reporting standards in future research.

Reviewer #3 (Remarks to the Author):

I commend the author team for their efforts in enhancing the paper and appreciate the thoughtful revisions that have been made. However, I believe there is one area that could benefit from further adjustment.

You mention that unpublished sources were excluded to introduce an additional layer of methodological rigour to the studies. While I understand the rationale behind this decision, I suggest reconsidering this approach. Not all researchers are inclined to publish their findings in scientific journals, and there are cases where well-conducted studies remain unpublished. Conversely, poorly conducted studies can still find their way into publication. Thus, excluding unpublished sources might inadvertently overlook valuable insights and data or create bias in the results. Additionally, reducing heterogeneity is not a very good reason to exclude these sources as this approach does not address the root cause of heterogeneity and heterogeneity itself should simply reflect what is present in the literature.

I encourage the authors to at least acknowledge this possibility and the limitations of their approach in the limitations section, as in my mind this is by far the biggest limitation of the meta-analysis and it is not really addressed in much depth at all.

Other than that, the paper has improved and I wish the authors all the best in their research.

Version 2:

Decision Letter:

Our ref: NATHUMBEHAV-25010440B

6th January 2026

Dear Dr. Kritz,

Thank you for submitting your revised manuscript "Solo or team? A meta-analysis comparing individual vs. group-based approaches on physical activity, functional, psychosocial, and health outcomes." (NATHUMBEHAV-25010440B). It has now been seen by the original referees and their comments are below. As you can see, the reviewers find that the paper has improved in revision. We will therefore be happy in principle to publish it in Nature Human Behaviour, pending minor revisions to satisfy the referees' final requests and to comply with our editorial and formatting guidelines.

We are now performing detailed checks on your paper and will send you a checklist detailing our editorial and formatting requirements within two weeks. Please do not upload the final materials and make any revisions until you receive this additional information from us.

Please do not hesitate to contact me if you have any questions.

Sincerely,

[redacted]

[redacted]
Nature Human Behaviour

Reviewer #1 (Remarks to the Author):

Thank you for the opportunity to review the revised manuscript entitled "Solo or team? A meta-analysis comparing individual and group-based approaches on physical activity, functional, psychosocial, and health outcomes." I commend the authors for their meticulous and comprehensive response to the previous round of review. The revisions have successfully addressed every point raised: the expanded reporting of heterogeneity (including prediction intervals and variance components) and the transparent handling of outliers via the new Supplementary File 3 provide the necessary methodological rigor, while the addition of the z-curve analysis and the well-justified SESOI for equivalence testing further bolster the robustness of the results. Furthermore, the integration of equity and cost-effectiveness considerations into the discussion regarding online versus face-to-face formats significantly enhances the study's practical impact. As the authors have fully complied with all requests and improved the clarity and depth of the analysis, I have no further recommendations and recommend the manuscript for acceptance.

Reviewer #3 (Remarks to the Author):

The authors have responded to the feedback and I think the paper is much improved as a result. I wish them all the best in their future research in this area.

Open Access This Peer Review File is licensed under a Creative Commons Attribution 4.0 International License, which permits use, sharing, adaptation, distribution and reproduction in any medium or format, as long as you give appropriate credit to the original author(s) and the source, provide a link to the Creative Commons license, and indicate if changes were made.
In cases where reviewers are anonymous, credit should be given to 'Anonymous Referee' and the source.
The images or other third party material in this Peer Review File are included in the article's Creative Commons license, unless indicated otherwise in a credit line to the material. If material is not included in the article's Creative Commons license and your intended use is not permitted by statutory regulation or exceeds the permitted use, you will need to obtain

permission directly from the copyright holder.
To view a copy of this license, visit https://creativecommons.org/licenses/by/4.0/

Response to Reviewer #1

Overall comments:

Comment 1: Thank you for the opportunity to review "Solo or team? A meta-analysis of individual vs. group-based studies on physical activity, functional, psychosocial, and health outcomes." This study aims to update and extend Burke et al.'s (2006) meta-analysis comparing group-based and individual physical activity interventions. The meta-analysis provides a valuable update with a large sample and advanced methods, aligning well with Nature Human Behaviour's focus on human behaviour and social challenges. The manuscript follows the necessary steps by adhering to PRISMA guidelines and conducting a comprehensive analysis to achieve the study's goals. It is clearly written, with a logical flow from methods to results and discussion. The detailed reporting of moderator analyses and sensitivity tests enhances transparency. Furthermore, the discussion effectively situates the findings within the existing literature, referencing both historical and contemporary studies.

Response: We appreciate the reviewer's positive assessment of the manuscript's clarity, methodological rigor, and relevance. We have carefully addressed all critical issues raised below.

Comment 2: The high heterogeneity remains poorly explained despite moderator analyses, weakening confidence in pooled effect sizes. Subgroup analyses (e.g., by population—healthy vs. clinical—or intervention duration) are needed to pinpoint variability sources and refine conclusions.

Response: We thank the reviewer for highlighting the issue of heterogeneity. We agree that high heterogeneity is an important limitation to address, though we also note that moderate to high heterogeneity is a common feature of meta-analyses-particularly those aiming to answer broad research questions across diverse interventions, as was the case in our review. The Cochrane Handbook, which represents the current gold-standard approach to conducting meta-analyses, acknowledges this challenge, stating that "variation across studies (heterogeneity) must be considered, although most Cochrane Reviews do not have enough studies to allow for the reliable investigation of its causes" (Higgins et al., 2023; Cochrane Handbook, Chapter 10). While our review is not a Cochrane Review, we find this point illustrative of the broader methodological constraints often encountered in meta-analytic work.

Subgroup analyses, as suggested by the reviewer, are indeed one approach to identifying sources of variability in the data. However, moderator analyses using meta-regression are an equally valid and in the case of continuous moderators, a more statistically powerful option. We employed meta-regression because it is functionally equivalent to subgroup analysis but allows for the inclusion of both categorical and continuous variables (e.g., health status, age, intervention duration), making it better suited to the complexity and diversity of the included studies. Regression coefficients from these models (reported in Tables 1–2) indicate differences in effect sizes relative to a reference category and permit meaningful comparisons across subgroups.

To address the reviewer's request for greater clarity, we have amended the Methods section to more explicitly describe the moderator analysis **(page 27, lines 627–636):**

> *"Second, we examined effects of potential moderators by including the following variables as covariates in a meta-regression: group type (the style of group setting used, such as online or standard face-to-face groups), group leader (the individual leading the group), health status (whether participants were healthy or had a specific medical condition), age (average age of the sample), gender (the proportion of female identifying participants in the sample), sessions per week (the frequency that groups or individuals exercised at), intervention duration (the number of weeks the intervention ran for), and study quality (the study's methodological and reporting quality rating). This moderator analysis allowed us to compare effect sizes across different moderator categories/quantities."*

"

Importantly, the moderator variables included health status (e.g., healthy vs. clinical population) and intervention duration/frequency, which align directly with the reviewer's suggestion to examine these factors as potential sources of heterogeneity. Significant moderators and their impact on the overall effect sizes are reported and discussed throughout the Results section (see page 9 subsections under "*Controlling for Significant Moderators*").

We remain cautious about undertaking any further post hoc exploratory subgroup analyses that were not pre-specified in our protocol. As the Cochrane Handbook (Section 10.10.3) cautions, conclusions drawn from post hoc analyses hold little informational value, should be interpreted conservatively, and can produce misleading results (e.g., false positives).

Higgins, J. P. T., Thomas, J., Chandler, J., Cumpston, M., Li, T., Page, M. J., & Welch, V. A. (Eds.). (2023). *Cochrane handbook for systematic reviews of interventions* (Version 6.4). Cochrane. https://training.cochrane.org/handbook

Comment 3: Moderate GRADE ratings for most outcomes (low for psychological) stem from imprecision, bias risks (e.g., lack of blinding), and reliance on self-reports, casting doubt on the stability of the results. A sensitivity analysis excluding indirect psychological measures (e.g., general well-being) and focusing on specific constructs (e.g., loneliness) could strengthen validity.

Response: We thank the reviewer for this valuable comment.

While refining the scope of psychological measures would address some of these concerns, it would not fully mitigate other sources of bias (e.g., lack of blinding) inherent in the primary studies, which affect the overall GRADE ratings.

With regard to conducting further sensitivity analyses, we are mindful of Cochrane guidance indicating that such post hoc analyses should be interpreted with caution, as they are best suited for hypothesis generation rather than explaining observed effects. Nonetheless, we appreciate the reviewer's suggestion and have noted the potential value of such approaches for future meta-analytic work in the discussion.

We agree that focusing on more specific psychological constructs, such as loneliness rather than broader measures of well-being, could help reduce imprecision and improve construct validity. We have now addressed these points in the revised Discussion section (**page 19, lines 438–446**).

> "*Psychological outcomes in our review were particularly affected by imprecision, risks of bias (e.g., lack of blinding), and heavy reliance on self-report measures, raising concerns about the robustness of the associated findings. While self-report remains a necessary method for assessing many psychological constructs, future research may benefit from prioritizing more specific and well-validated measures (e.g., affect, loneliness) over broad or diffuse constructs (e.g., general psychological well-being). Additionally, meta-analytical comparisons of group-based versus individual interventions targeting clearly defined psychological outcomes could offer greater granularity regarding intervention effects and advance construct-specific conclusions.*
>
> *."*

Comment 4: The shift to significance for functional outcomes only after outlier removal raises concerns about selective reporting. A detailed justification for exclusions (e.g., study-specific traits) and consistent reporting of results with and without outliers across all outcomes are essential.

Response: We fully agree that transparency regarding outlier handling is critical, particularly when such decisions influence the interpretation of findings.

In our manuscript, the primary analyses included all studies, including those with outlying effect sizes. As part of our pre-specified sensitivity analyses, we examined the potential influence of these outliers on overall results. These sensitivity analyses, along with other subgroup and design-based comparisons, are clearly presented in their own subsections, and we have taken care to distinguish them from our main findings.

In light of the reviewer's concern, we have revised the *Discussion* section to ensure that the primary conclusions are based solely on the full-sample analyses. We now explicitly state that the sensitivity results (e.g., for functional outcomes) should be interpreted as hypothesis-generating and indicative of areas in need of further, more robust investigation (**pagea 16-17, lines 371-379**). The revised text reads:

> *"For functional outcomes (e.g., balance, coordination, muscle strength), we did not find a substantive difference in our primary analysis; however, sensitivity analyses indicated that group-based approaches became significantly more effective after removing outliers, as well as when restricting the analysis to experimental/quasi-experimental studies (i.e.,*

excluding correlational studies). While results of sensitivity analyses should be interpreted with caution, they can be useful for hypothesis generation[55]. We suggest that in some cases (e.g., when groups are formalised such as in experimental studies), group structure and support may better promote functional gains compared to individual contexts, as individuals in a group can benefit from observing others and receiving feedback."

We also appreciate the reviewer's point regarding consistency in reporting with and without outliers. To further clarify our methodological decisions, we have included a justification for the exclusion of outliers in the equivalence testing (TOST) analyses. Specifically, extreme values resulted in non-normality of the distribution, violating the test's assumptions. While robust versions of TOST exist (see- https://aaroncaldwell.us/TOSTERpkg/articles/robustTOST.html), they have not, to our knowledge, been extended to meta-analytic data. This explanation has now been added to the relevant sections of the Results (**pages, 9, 12**).

Comment 5: Moderator analyses lack explanatory power, and low study power (per funnel plots) risks missing true effects. Testing moderator interactions (e.g., group type × leader) and adding a post-hoc power calculation could help clarify these weaknesses.

Response: We thank the reviewer for this constructive suggestion. We agree that the explanatory power of moderator analyses in meta-analytic work can be limited, particularly when faced with substantial heterogeneity and inconsistent reporting across primary studies. As noted, this is a common challenge in meta-analysis, even in reviews of the highest methodological quality.

In our review, we explored several moderators grounded in both theory and prior empirical findings. However, interactions such as group type × leader were not consistently reported in the primary studies, limiting our capacity to model these effects reliably. Furthermore, detecting interaction effects generally requires substantially greater statistical power than detecting main effects (see Sommet et al., in press, https://journals.sagepub.com/doi/10.1177/25152459231178728) . As such, we believe that conducting additional post hoc analyses of interaction effects—given the current dataset—would likely yield underpowered results with limited explanatory value.

We appreciate the reviewer's suggestion regarding a post hoc power analysis. However, we note that there is broad consensus in the methodological literature advising against post hoc power calculations, as such analyses are largely a function of the observed p-value and do not provide additional information beyond what is already conveyed by confidence intervals (Valentine et al., 2010; Borenstein et al., 2009). In line with these recommendations, we have consistently reported confidence intervals throughout the manuscript to communicate the precision and uncertainty associated with our effect estimates.

Nonetheless, we think the reviewer's suggestion to consider power is highly valuable. Aligning with best practice recommendations (Hedges & Pigott, 2001), we used power curves (implemented using the dmetar package; Harris et al., 2020) to estimate the smallest reliably detectable (i.e., with 80% power) effect given the sample size obtained in our meta-analyses and assuming high heterogeneity. The results of this analysis are now provided in the Search Overview subsection of Results **(page 6, lines 128-131).** We now state:

> *"Power curves indicated the minimal detectable effect size at 80% power, given the number of extracted effects and assuming high heterogeneity, was g = .058 for PA outcomes, g = .052 for functional outcomes, g = .062 for psychological outcomes and g = .050 for health outcomes[33,34]"*

We hope this additional information addresses the reviewer's concern and strengthens the transparency of our analytic approach.

Comment 6: The discussion does not fully explore practical implications (e.g., scalability) or theoretical alignment (e.g., self-determination theory), limiting its reach. Providing concrete examples (e.g., policy applications) and deeper theoretical synthesis would enhance the manuscript's contribution to the field.

Response: We thank the reviewer for highlighting the need to strengthen the practical and theoretical contributions of the *Discussion*. In response, we have revised the section to:

- Expand on the practical implications of our findings, including a clearer discussion of scalability, resource needs, and digital delivery formats. In particular, we highlight how digital interventions, which showed comparable outcomes to in-person formats, may enhance accessibility for underserved populations (e.g., rural communities or older adults with mobility constraints).
- Include policy-relevant examples to demonstrate real-world applicability, such as implications for remote delivery of physical activity programs and scalable rehabilitation models for older adults and in workplace settings.

For example, we now include in the discussion section on **page 18 (lines 403-411)** the following:

> "*The comparable effectiveness of online and face-to-face groups across psychological, functional and PA-related outcomes supports the potential of online interventions to reach populations who face barriers to in-person participation, such as mobility limitations, transportation challenges, or geographic isolation. Building on these findings, policy initiatives could integrate virtual PA programs into public health strategies, for example, by offering virtual and group-based rehabilitation programs for older adults or embedding digital PA support into chronic disease prevention and management initiatives. Workplace wellness policies could also leverage virtual, group-based PA*

programs to enhance reach and scalability, particularly for national PA promotion efforts in rural or resource-limited settings."

- More explicitly integrate relevant theoretical frameworks. We now discuss on **pages 16-page 18** how our findings align with key principles of Self-Determination Theory (e.g., relatedness and autonomy) and Social Cognitive Theory (e.g., observational learning, modelling, and vicarious reinforcement), particularly in relation to improvements in functional outcomes,

We believe these revisions have improved the scope and relevance of the *Discussion* and thank the reviewer for prompting these enhancements.

Specific Comments Following the Journal's Reviewer Guidelines

Comment 7a:
Study Rationale
The study presents a strong rationale for updating Burke et al.'s (2006) meta-analysis but lacks the precision needed to convincingly justify its urgency and originality. The statement that "a multitude of relevant new studies" have emerged (line 101) is too vague, as it does not provide concrete examples or preliminary evidence (e.g., the number of studies published since 2006, notable shifts in effect sizes). Without such details, the argument for why an update is necessary feels assumed rather than empirically justified. The authors should consider strengthening this section with quantitative indicators, such as citation trends or methodological advancements in PA interventions, to reinforce the study's relevance.

Response: In response, we have revised the rationale in the Introduction to include a specific quantitative indicator and key methodological advancements. The revised text in the introduction on **page 5 (lines 91-92)** now states:

> *"First, this review was published nearly 20 years ago,* ***and 58 relevant new studies have since been published and included in this meta-analysis****."*

We have included details that emphasize key methodological developments since the Burke et al. meta-analysis in the introduction Specifically, we state on **page 5, lines 93-98:**

> *"However, with technological developments and the introduction of online-delivered interventions[25], an updated understanding of the literature requires expanding the definition of a PA group to include online groups[26], and newer technologies such as virtual reality platforms[27]. These modalities were not widely accessible when Burke et al. conducted their review, potentially limiting the generalizability and scalability of their findings."*
> *."*

And now clarified on **page 5, lines 107-108** the following:

"Finally, since 2006, the use of device-based PA measures (e.g., Actigraphs) has enabled more sophisticated assessments of PA[30]."

In addition, we clarified **on page 5-6, lines 108-112** our analytic scope by stating:

"Differing from the Burke et al. meta-analysis, we excluded dyadic interventions, as their unique interpersonal dynamics differ from those in group or individual settings and could introduce conceptual heterogeneity [31,32]. We also included only studies directly comparing group-based and individual PA conditions, to specifically address the question about their relative effectiveness."

With these clarifications we have strengthened the rationale with greater specificity and empirical support. We also clarified that we included only studies *directly* comparing group-based and individual PA conditions, to specifically address the question about their relative effectiveness.

Comment 7b: Additionally, while the inclusion of online groups and virtual reality (line 106) is a valuable expansion, the rationale does not clearly connect these modalities to the specific limitations of Burke et al.'s findings.

Response: We thank the reviewer for this insightful comment. To more clearly connect the inclusion of online groups and virtual reality to the limitations of the original review, we have revised the rationale section in the Introduction (**page 5, lines 93-98**) to state:

"However, with technological developments and the introduction of online-delivered interventions[25], an updated understanding of the literature requires expanding the definition of a PA group to include online groups[26], and newer technologies such as virtual reality platforms[27]. These modalities were not widely accessible when Burke et al. conducted their review, potentially limiting the generalizability and scalability of their findings."

Comment 8: A stronger statement is needed to explain why the rise of digital interventions necessitates reassessment, particularly in light of changes in exercise behavior post-COVID, increased accessibility through technology, or different motivational structures in virtual environments. The current study's aim (line 116) is also broad and does not explicitly pose a specific research question or hypothesis. The authors should consider elaborating on whether the study seeks to determine if online groups outperform individual PA interventions post-COVID or whether digital interventions lead to higher adherence

rates compared to in-person groups. Clarifying this would provide a sharper focus and strengthen the study's positioning.

Response: We thank the reviewer for this valuable suggestion. In response, we revised the *Introduction* to clearly articulate the rationale for reassessing digital interventions in the current context and sharpened the study's aim. Specifically, we added to **page 5, lines 99-106** the following sentence to contextualize the need for an updated review:

> *"This shift is particularly relevant considering the post-COVID increase in remote and home-based physical activity formats, which offer enhanced accessibility, especially for underserved or rural populations[25]. Moreover, digital interventions may introduce different motivational dynamics (e.g., altered social presence, autonomy, or feedback structures) that can influence adherence and outcomes in ways that differ from traditional in-person settings [28,29]. These contextual changes highlight the need to reassess the relative effectiveness of group-based (online and in-person) versus individual interventions, and to update public health messaging to reflect this evolving delivery landscape"*

We also explicitly clarified in the Introduction (see **page 6, lines 117-123**) our research objective and sub-questions (which were not about testing pre-COVID vs post-COVID differences) as follows:

> *Specifically, we examined whether group-based interventions, delivered either in-person or online, differ in effectiveness from individual-based interventions.*
>
> *To guide our analysis, we addressed the following sub-questions:*
>
> *Do online group-based interventions perform comparably to in-person groups?*
> *Does intervention mode (online vs. in-person) moderate the relationship between group format and behavioral, functional, psychosocial, and physical outcomes?*

Comment 9: Key Results
The key findings—statistical equivalence for PA and health outcomes, alongside a significant advantage for group-based interventions in functional outcomes after outlier removal—are important but require greater clarity and stability. The shift to significance for functional outcomes only after excluding outliers (line 396) raises questions about the robustness of this finding, given that statistical significance was only achieved after outlier exclusion. Without a transparent justification for why these specific cases were removed, the reliability of this finding is weakened. The authors should consider providing study-specific details explaining whether exclusions were based on data errors, methodological inconsistencies, or extreme effect sizes.

Response: We appreciate this observation and agree that more transparency strengthens the interpretation. Outliers were identified using standard influence diagnostics (standardized residuals > ±3 and Cook's distance values >3x the mean), consistent with recommended meta-analytic procedures (Gucciardi et al., 2022- https://doi.org/10.1080/1750984X.2021.1946835). The excluded studies were not removed due to data errors or methodological inconsistencies but

due to statistically extreme effect sizes that produced heavy tailed (i.e., non-normal) effect size distributions and may have disproportionately influenced the pooled estimate. Importantly, violations of normality can influence meta-analytic conclusions (Liu et al., 2023- https://doi.org/10.1186/s12916-023-02823-9) and thus it is necessary to examine the impact of these outlying studies. We have now added a brief explanation in the Results **(pages, 9, 12)** and included study-level details for the removed outliers in the Supplementary Materials **(Table S3).** Importantly, the direction and overall pattern of results remained consistent, and the finding for functional outcomes was strengthened, not reversed, by the exclusion. **We now state:**

> *"These outliers contributed to heavy-tailed (i.e., non-normal) effect size distributions, which can influence meta-analytic results (Liu et al., 2023). Aligning with our protocol, we conducted a sensitivity analysis to examine the impact of these outlying studies."* **(page 9, lines 190-193)**
>
> *We identified outlying effect sizes for all secondary outcomes, which contributed to heavy tailed (i.e., non-normal) effect size distributions. Aligning with our protocol, we conducted sensitivity analyses to examine the impact of these effects"* **(page 12, lines 252-254)**

Comment 10: Additionally, small, non-significant effect sizes for PA (line 312) and wide confidence intervals (e.g., [-.061, .233]) highlight potential imprecision, yet these concerns are not directly addressed. Reporting results both with and without outliers for all outcomes would enhance transparency and allow readers to assess the robustness of conclusions.

Response: We agree that clear reporting of results, including consideration of potential imprecision and the impact of outliers, is essential for transparency.

In our manuscript, we report the main results, including all studies, outliers included, at the beginning of each relevant subsection (see *Primary Outcome: Comparison of Group-Based and Individual Conditions on PA*, **page 8**; *Secondary Outcomes: Comparison of Group-Based and Individual Studies on Functional, Psychological, and Health Outcomes*, **page 10**). We have attempted to clearly demarcate these results from sensitivity analyses in which we removed outliers by providing these analyses in separate subsections (see *Outliers* (**page 9 and page 12** for primary and secondary outcomes, respectively).

Following the reviewer's suggestion, we have revised the Methods and Results sections to further clarify our decisions around outlier detection and sensitivity analyses in which we removed outliers. We would also like to note that these procedures were pre-specified in our study protocol and conducted in line with our planned analytical strategy.

We have also amended the Discussion to include a commentary on imprecision in the primary outcome **(page 20, lines 446-464)**.

"We observed imprecision in both primary and secondary outcome effect size estimates, indicating uncertainty around the true size of differences between group and individual intervention programs. This issue is potentially attributable to the substantial heterogeneity observed in all effect sizes, which underscores variability in study designs, populations, and intervention implementation across studies. Differences in participant demographics, settings (e.g., community versus clinical), and intervention characteristics (e.g., frequency, duration) likely contributed to inconsistent results. Although our various sensitivity analyses demonstrated that results remained largely unchanged after adjusting for a variety of potential between-study differences, we suggest that efforts to address heterogeneity in future studies by standardizing intervention designs or performing other subgroup/moderator analyses could clarify which specific factors influence the effectiveness of the two different formats. For example, we were unable to examine the influence of intervention setting in the current study due to inconsistent or incomplete reporting across the included studies. This step would enhance comparability and help isolate the conditions under which group or individual interventions work best and improve precision of effect size estimates."

Comment 11: Furthermore, the discussion on functional outcomes should explore why this specific domain showed a group advantage—was it due to social modelling, enhanced accountability, or leader feedback? The authors should consider providing a clearer mechanistic explanation, possibly drawing from social cognitive theory (e.g., observational learning, vicarious reinforcement), to strengthen the interpretation.

Response: We agree that understanding the mechanisms behind the group advantage in functional outcomes is important. Our data did not allow us to directly test mechanisms such as social modelling, or leader feedback. However, we have expanded the discussion to include theoretical explanations, drawing on social cognitive theory (see revised *Discussion*, **pages 16-18**) We now highlight the potential roles of observational learning, vicarious reinforcement, and perceived social support, and suggest that these mechanisms be explicitly examined in future primary studies.

Comment 12:
Validity
The validity of the conclusions is weakened by unresolved methodological concerns. The substantial heterogeneity remains despite the inclusion of moderators such as group leader type (line 327), suggesting that key explanatory factors remain unaccounted for. The moderate-to-low GRADE ratings (e.g., low for psychological outcomes, line 437) further highlight limitations in measurement precision, particularly due to reliance on self-reported data and the absence of blinding in many studies.

Response: We appreciate the reviewer's concern but neither points they raise are unusual. In fact, we have yet to come across a published meta-analysis in which heterogeneity is fully explained by the addition of moderators. The moderate to low GRADE ratings are also very common in most published meta-analyses; they reflect the quality of the primary studies and not the quality of the meta-analysis. While we acknowledge that heterogeneity remains, we believe

our aforementioned analytic steps have meaningfully addressed this common challenge in meta-analysis by partitioning and explaining variance to the extent permitted by the available data. We now more explicitly acknowledge the remaining unexplained heterogeneity as a limitation in the revised *Discussion* **(see response to Comment 10).**

Regarding the GRADE ratings, we agree that the low certainty for psychological outcomes reflects limitations in measurement precision and study design (e.g., reliance on self-reported data, absence of blinding). These issues are clearly acknowledged in both the GRADE summary and the amended *Discussion* **(see response to Comment 3).**

Comment 13:
Furthermore, the dependence of functional outcome significance on outlier removal (line 331) introduces uncertainty into the conclusions. Addressing these concerns requires conducting additional subgroup analyses (e.g., stratified by population type—clinical vs. non-clinical—or intervention setting). A sensitivity analysis that excludes studies relying solely on self-reported psychological outcomes would help assess whether measurement bias affects results. Finally, the authors should explicitly acknowledge these methodological limitations to avoid overgeneralization.

Response: We thank the reviewer for these constructive suggestions. As noted above, we conducted sensitivity analyses excluding statistical outliers and now clearly report how this affected the significance of functional outcomes. While statistical significance was achieved only after removing outliers (identified using standard influence diagnostics), the direction and magnitude of the effect remained consistent. To avoid overstating the findings, we have revised the *Discussion* accordingly **(see response to Comment 4).**

We also confirm that clinical vs. non-clinical population status was included as a moderator in our analyses (see Tables 1 and 2). However, we recognize that residual heterogeneity remains and agree that future research should explore additional potential moderators. In particular, as the reviewer suggested, intervention setting is a relevant factor; unfortunately, inconsistent or unclear reporting across studies precluded meaningful subgroup analysis in this case. We now highlight this gap and recommend it as a priority for future work **(please see page 19).**

Regarding the suggestion to exclude studies relying solely on self-reported psychological outcomes, we note that all psychological outcomes in the literature are self-reported. Conducting such an analysis would leave very few effect sizes, raising concerns about the robustness of the resulting estimates (Cochrane Handbook, Chapter 10). We also note, as discussed in previous responses, that additional post-hoc sensitivity analyses may offer limited value and risk overfitting the data.

Finally, we have revised the Discussion to more explicitly acknowledge methodological limitations, particularly in relation to measurement precision and reliance on self-reported outcomes.

Comment 14: Significance
While the study's significance lies in updating Burke et al., the small effect sizes (line 312) and alignment with prior findings (line 457) make the contribution feel incremental rather than transformative. The discussion highlights how modern PA interventions include online groups (line 506), but the practical implications remain vague.

Response: We respectfully disagree that our contribution is merely incremental. While our work builds on Burke et al., we believe it constitutes a meaningful expansion for the following reasons:

1. As outlined in the revised *Introduction* and *Discussion*, we do not merely update the prior meta-analysis. We expand it by incorporating a broader range of delivery formats—including online groups and VR-enhanced interventions—that reflect the evolving public health and technological landscape in the recent years. This enables us to assess intervention formats that were not previously available or evaluated.
2. Our analysis responds to significant shifts in exercise behaviour, accessibility, and delivery mechanisms. By including more recent studies, many of which use device assessed physical activity measures, we provide a more rigorous and contemporary assessment of group versus individual physical activity interventions.
3. We did not only test for superiority, but also for equivalence between formats. Finding no significant differences has practical implications: if online and face-to-face interventions yield similar outcomes, online and/or group-based interventions may be favoured in terms of scalability and cost-efficiency. This is particularly relevant for policymakers and program designers aiming to maximize reach within constrained budgets. This has now been clarified in the discussion section.
4. In the current era of open science, dismissing the value of non-significant or modest effects risks reinforcing publication bias. Our transparent reporting, moderator analyses, and confidence intervals contribute to a more balanced and accurate evidence base—critical for advancing cumulative knowledge in this field.
5. We further enhance the contribution by integrating relevant theoretical frameworks (e.g., Self-Determination Theory, Social Cognitive Theory) and articulating clear implications for practice, including, equitable access, and digital delivery models in underserved settings. **Please see the discussion section, page 16-18.**

Comment 15: To enhance the study's impact, the discussion should explicitly connect findings to real-world applications. For example, the equivalence of online and in-person PA groups could be leveraged to support scalable virtual PA programs in rural communities or among populations with mobility constraints.

Response: We thank the reviewer for this valuable suggestion. As requested, we have explicitly connected our findings to real-world applications in the revised *Discussion*. For example, we now state:

> *"The similar effectiveness of online and face-to-face groups across psychological, functional and PA-related outcomes supports the potential of online interventions to*

reach populations who face barriers to in-person participation, such as mobility limitations, transportation challenges, or geographic isolation. Building on these findings, policy initiatives could integrate virtual PA programs into public health strategies, for example, by offering virtual and group-based rehabilitation programs for older adults or embedding digital PA support into chronic disease prevention and management initiatives. Workplace wellness policies could also leverage virtual, group-based PA programs to enhance reach and scalability, particularly for national PA promotion efforts in rural or resource-limited settings." **(page 18, lines 403–411)**

Comment 16: Additionally, if group-based PA improves functional outcomes, this insight could inform rehabilitation programs for older adults or clinical populations where functional ability is a primary concern. The authors should explicitly link results to pressing public health challenges, such as declining physical activity rates post-COVID or digital health interventions in underserved areas.

Response: We thank the reviewer for this insightful suggestion. In the revised *Discussion*, we have now explicitly linked the observed group-based advantage in functional outcomes to potential applications in rehabilitation contexts, particularly for older adults and clinical populations with reduced physical functioning. For example, in **page 17, lines 385–390**, we state:

> *"These mechanisms may be particularly salient for functional tasks requiring coordination or technique, which benefit from visual learning and real-time adjustment- as compared to more general PA outcomes, where intensity and consistency may matter more than group dynamics. Such functional advantages suggest that group-based approaches could be valuable for rehabilitation programs targeting older adults and clinical populations, where preserving functional ability is critical [41]."*

We also highlight how these findings address current public health challenges, including and the relevance of scalable digital interventions in underserved and rural communities. These real-world implications strengthen the clinical and public health relevance of our findings. **Please see lines 403-406, page 18 where we state:**

> *"The similar effectiveness of online and face-to-face groups across psychological, functional and PA-related outcomes supports the potential of online interventions to reach populations who face barriers to in-person participation, such as mobility limitations, transportation challenges, or geographic isolation"*

Comment 17: Data and Methodology
The methodology is rigorous but contains gaps that weaken data quality and comprehensiveness. The exclusion of dyadic interventions (line 132) is not sufficiently justified, as dyads also involve social influences that could be relevant to PA interventions. Either providing a stronger theoretical basis for excluding dyads or reconsidering their inclusion could enhance the study's comprehensiveness.

Response: As noted in the revised Introduction **(page 5, lines 108–110),** we have clarified our rationale for excluding dyadic interventions based on conceptual distinctions in social dynamics. Specifically, we now state:

> *"Differing from the Burke et al. meta-analysis, we excluded dyadic interventions, as their unique interpersonal dynamics differ from those in group or individual settings and could introduce conceptual heterogeneity [31,32]"*

Dyadic interventions typically involve reciprocal interdependence and individualized feedback, which are conceptually distinct from the collective identity, group cohesion, and social facilitation mechanisms that underpin group-based physical activity interventions. These theoretical and structural differences justify their exclusion and ensure analytical consistency across the included studies. We hope this elaboration strengthens the conceptual coherence of our review.

Comment 18: Additionally, missing data from non-responsive authors (line 190) introduces potential selection bias, yet the manuscript does not specify how many studies were affected or whether imputation was considered. Reporting the proportion of missing data and assessing its potential impact on findings would increase transparency.

Response: We appreciate this important point. As now specified in the revised manuscript (**Methods: Data Extraction Process, page 24, lines 555–557**), we contacted authors of five studies to obtain missing data; two did not respond, and their studies were excluded due to insufficient information. Because the missing data involved key outcome variables necessary for effect size calculation, imputation was not feasible. Given that only two studies were excluded on this basis, we consider the risk of selection bias to be minimal. Nonetheless, we have now explicitly reported this number to increase transparency.

Comment 19: The high heterogeneity (e.g., line 313) is acknowledged but not fully addressed (line 542), making it difficult to interpret pooled estimates. Conducting additional subgroup analyses, particularly by intervention type or delivery format (e.g., online vs. in-person), could help address this concern.

Response: As noted above, we addressed heterogeneity through a three-level random-effects model and moderator analyses, which included intervention type and delivery format (online vs. in-person) as moderators (see Tables 1 and 2). Importantly, our moderator analyses was implemented using meta-regression (i.e. moderators were included as covariates in the model) and can be considered as equivalent to subgroup analysis, but is more appropriate for our review as it allows for continuous (e.g. age) as well as categorical (e.g. healthy vs. clinical population) study characteristics to be investigated (see Cochrane Handbook – Section 10.11). A sub-group analysis would also result in loss of statistical power. We have clarified the methods used for our moderator analysis and hope this makes our approach more transparent. Specifically, we now state on **page 27-28, lines 626-635:**

> *"Second, we examined effects of potential moderators by including the following variables as covariates in a meta-regression: group type (the style of group setting used,*

such as online or standard face-to-face groups), group leader (the individual leading the group), health status (whether participants were healthy or had a specific medical condition), age (average age of the sample), gender (the proportion of female identifying participants in the sample), sessions per week (the frequency that groups or individuals exercised at), intervention duration (the number of weeks the intervention ran for), and study quality (the study's methodological and reporting quality rating). This moderator analysis allowed us to compare effect sizes across different moderator categories/quantities."

While heterogeneity remains due to the diversity of study designs and outcome measures, the inclusion of these moderators explained some, but not all heterogeneity, in several domains. As explained above, this is the case for almost all published meta-analyses.

Comment 20: Analytical Approach
The analytical approach is methodologically sound but presents limitations in explanatory power. The three-level meta-analytic model and TOST equivalence testing are appropriate, but moderator analyses (e.g., group leader, line 324) fail to fully explain heterogeneity (line 327). This suggests that additional moderator interactions should be tested, such as whether the impact of group type depends on the leader's role or whether intervention duration influences results differently across settings.

Response: We appreciate this thoughtful suggestion. As noted above, we employed a three-level meta-analytic model and conducted a comprehensive set of moderator analyses (see Tables 1 and 2), including variables such as group type, leader role, intervention duration, clinical status, and delivery format. While these moderators helped reduce unexplained variance in some outcome domains and allowed comparisons between different levels of each moderator, heterogeneity remained—likely reflecting the complex interplay of factors across a diverse evidence base.

We agree that testing interactions between moderators (e.g., group type × leader role, duration × setting) could offer potential mechanisms for observed effects. However, many studies lacked sufficient information to allow reliable estimation of such moderator x moderator effects. Effectively, such analyses would necessitate access to the raw data sets of each included study in order to extract the required information. Moreover, post-hoc sensitivity analyses to explain potential sources of heterogeneity offer limited explanatory power: "reliable conclusions can only be drawn from analyses [conducted to explore heterogeneity] that are truly pre-specified before inspecting the studies' results, and even these conclusions should be interpreted with caution. Explorations of heterogeneity that are devised after heterogeneity is identified can at best lead to the generation of hypotheses. They should be interpreted with even more caution and should generally not be listed among the conclusions of a review." (Cochrane Handbook, Section 10.10.3).

We have now noted this as a limitation in the Discussion and suggested it as an important direction for future meta-analyses with larger or more harmonized datasets or specific research questions addressing these hypotheses. Specifically, we now state in the Discussion on page 20-21, in **lines 468-472:**

“Interaction effects between moderators (e.g., group type × leader role or duration × setting) may offer additional explanatory insight, but limited reporting in the primary studies precluded reliable testing. Larger datasets or access to the raw data from all primary studies may enable future analyses to systematically explore these interactions.

.”

Comment 21: The low statistical power to detect small-to-moderate effects (line 352) raises concerns about false negatives, particularly for secondary outcomes. Conducting a post-hoc power analysis would clarify whether the study was adequately powered to detect meaningful effects.

Response: We acknowledge the reviewer’s concern regarding statistical power. As noted above, we addressed questions about study power using power curves to determine the smallest possible effect size that could be reliably detectable (i.e., with 80% power) given our obtained sample. We interpreted effects smaller than these limits cautiously—particularly for psychological outcomes with low GRADE ratings. Power curve analysis is considered a statistically valid alternative to a post-hoc power analysis, which is generally not advised in the methodological literature.(see Sommet et al., in press, https://journals.sagepub.com/doi/10.1177/25152459231178728)

Comment 22: *Furthermore, the handling of outliers, particularly their impact on functional outcomes (line 396), remains insufficiently justified. The authors should consider explaining the rationale behind outlier exclusion—whether due to data errors, methodological inconsistencies, or extreme effect sizes— to ensure replicability and reduce concerns about selective reporting.*

Response: We thank the reviewer for this important point. As noted above, outliers were identified using standard influence diagnostics—specifically, standardized residuals greater than ±3 and high Cook’s distance values **(page 27)**. These exclusions were not due to data errors or methodological inconsistencies but rather reflected statistically extreme effect sizes that may have disproportionately influenced the pooled estimates and/or invalidated analyses, such as the TOST tests, by reducing the normality of effect size distributions.

Comment 23: Technical Density, The technical density of the manuscript may hinder accessibility for readers unfamiliar with meta-analytic techniques. Sections explaining TOST (line 247) and the three-level model (line 242) are particularly dense, which may make interpretation challenging for a broader audience.

Response: We appreciate the reviewer’s concern and understand the importance of accessibility for a broad readership. However, we feel that omitting or overly simplifying details in this section could obscure key methodological information that may be especially relevant for readers with an interest in analytic rigor.

That said, we have made an effort to keep the descriptions concise and have included simplified explanations, particularly for less familiar techniques such as TOST. In response to this

comment, we have reviewed the relevant sections again and made some revisions to improve readability without sacrificing precision.

> *"To provide a brief primer on the interpretation of this procedure, a significant TOST test indicates that the two conditions produce statistically equivalent effect sizes, and we can thus accept the null hypothesis that there is no difference between the two. A non-significant TOST test indicates that there is insufficient precision to conclude either for or against the null hypothesis."* **(page 26-27, lines 611-615)**

In addition, parts of the Discussion were re-written to be accessible to readers who may not be familiar with the technical aspects of the analyses.

Comment 24:
To improve clarity, the manuscript could introduce a brief glossary or footnotes defining key statistical terms such as "TOST" and "Hedges' g" in more accessible language.

Response:
We appreciate the reviewer's suggestion and understand the value of enhancing clarity for a broad readership. We defined TOST and its purpose (i.e., to test evidence for the null hypothesis) when we introduced the acronym **(page 26),** provided readers with a primer to understand the test, and cite several papers that go into the depth in detail. We believe adding further information is beyond the scope of this paper and would further complicate the section, which as the reviewer pointed out in their previous comment is already one of the more technical parts of the manuscript.

Hedges g is perhaps one of the most commonly used effect size metrics in meta-analysis. It is a fundamental concept to meta-analysis that is covered in introductory textbooks on the topic (e.g., Bornstein, 2009 – cited in our review). We suggest most readers who have basic knowledge of meta-analysis would be familiar with Hedge's g and argue that it is beyond the scope of this review to provide readers with an explanation of fundamental meta-analytic principles.

We hope the rationale provided is acceptable and appreciate the reviewer's commitment to ensuring the paper is accessible and informative.

Comment 25: The discussion (line 451) references theories like self-determination theory (line 477) but does not thoroughly integrate them into the interpretation of findings. The authors should clearly explain how these theories support or challenge the results—such as whether social relatedness influences group-based PA adherence. Additionally, practical implications should be made more tangible by providing specific examples, such as how virtual group-based PA programs could be implemented in workplace wellness initiatives or public health campaigns. These refinements would enhance the manuscript's readability and real-world applicability.

Response: We thank the reviewer for this valuable suggestion. We have expanded the discussion to more clearly integrate self-determination theory, specifically highlighting how social relatedness and autonomy support may help explain the group-based advantage in functional outcomes. We also added concrete examples of how findings could inform real-world applications, such as implementing virtual PA groups in workplace wellness programs and public health initiatives targeting rural or mobility-limited populations. These additions, on **pages 16-18**, aim to enhance both theoretical interpretation and practical relevance.

We sincerely thank Reviewer 1 for their thoughtful and detailed feedback, which has helped us strengthen both the clarity and scientific contribution of the manuscript

Reviewer #2

Comment 1: This is a very useful and well conducted review, investigating a topic that has implications for resource allocation. The review is an update on one conducted previously, but appears warranted given the much larger number of studies available to provide greater confidence in findings. Particular strengths that provide confidence in the findings include the rigorous screening and data extraction process (incorporating but not reliant on AI), inclusion of online groups which had not been the case in the previous review, and greater availability of objectively measured PA of study outcomes. Overall the authors did an excellent job on reporting a complex set of analyses, and incorporation of multiple dimensions. I have only a couple of minor comments to add.

We would like to thank you for the very positive evaluation of our work. This is very much appreciated!

Introduction
- P5 Line 110 – minor point, but the suggestion that there is a lasting new normal of social distancing needs supporting. For many exercise facilities do not appear different from pre-pandemic arrangements, so evidence that this impression is wrong/limited is needed.

Response: Thank you for this suggestion. We retain that the pandemic led to an increase in remote-delivered physical activity interventions as just one justification for the study, but remove mention of a new normal.

Comment 2: Method have overstated the long-term behavioural impact of social distancing.

Response:

Thank you for this observation. We revised the introduction to avoid overstating the long-term behavioural impact of social distancing. The updated version emphasises the broader shift

toward remote and home-based physical activity formats post-COVID, without implying a permanent change in social behaviour. Specifically, we now state **on page 5, lines 99-105**:

> *"This shift is particularly relevant considering the post-COVID increase in remote and home-based physical activity formats, which offer enhanced accessibility, especially for underserved or rural populations[25]. Moreover, digital interventions may introduce different motivational dynamics (e.g., altered social presence, autonomy, or feedback structures) that can influence adherence and outcomes in ways that differ from traditional in-person settings [28,29]. These contextual changes highlight the need to reassess the relative effectiveness of group-based (online and in-person) versus individual interventions, and to update public health messaging to reflect this evolving delivery landscape."*

Comment 3: It was not clear in the Methods what would be done with the data on study quality and risk of bias. For ROB, it's stated that evidence would be downgraded where needed, but not what would happen in the analysis for a downgraded study.

Response: Thank you for this observation. Study quality ratings were included as a moderator in our analyses. One of the key advantages of using a moderator approach is that, by entering study quality as a covariate, we were able to adjust for the potential influence of study-level characteristics measured on a continuous scale. Specifically, in the study quality moderator analysis, we accounted for the potential impact of study quality on effect sizes.

The corresponding coefficients in **Tables 1–2** reflect how effect sizes varied as study quality improved. Because GRADE assesses the quality of evidence in the body of literature as a whole and not per individual study, we did not implement any changes in the analysis. We suggest that our moderator analysis adjusting for study quality is a good proxy for adjusting for risk of bias as poor study quality typically introduces a higher risk of bias.

Comment 3: - It would be useful to clearly articulate what a priori moderators were planned to be included in the analyses (p10 line 236).

Response: We thank the reviewer for this helpful comment. As suggested, we now clearly list all a priori moderators in the Methods section **(page 25, lines 566-568**). Specifically, we state:

> *"We pre-specified and a priori registered the following moderators for later meta-regression: group type, leader type, clinical status, age, gender, session frequency, delivery format, duration, and study quality."*

We sincerely thank Reviewer 2 for the thoughtful feedback. We look forward to your consideration of our revised manuscript.

Reviewer #3

Comment 1: Overall, there is a lot to like about this paper. I enjoyed the brevity, and the fact that it was really well written and easy to read. The meta-analysis is reported transparently, with much additional information accessible on the OSF. Moreover, results were pre-registered ahead of time on PROSPERO and the authors followed PRISMA protocols. The analyses seemed well justified and both competently and carefully done.

Response: We would like to thank you for the very positive evaluation of our work. This is very much appreciated!

That said, I do have some points of feedback that I note below in case the authors want to provide further clarity about some issues:

1. On page 1 (first paragraph) it is noted that a substantial proportion of adults fail to reach the recommended guidelines for PA. It might be worth briefly mentioning what these are for people who are not in this field

Response: Thank you for this helpful suggestion. We have added a clarifying sentence to the Introduction to improve accessibility for readers less familiar with current guidelines (**see page 3, Lines 42-44)**

> *"To achieve these benefits, the World Health Organization (WHO) recommends 150–300 minutes of moderate-intensity or 75–150 minutes of vigorous-intensity aerobic activity per week, along with muscle-strengthening exercises on two or more days[4]."*

Comment 2: On page 3 of the introduction, true groups are described. As someone who is not from the PA field, this still read as a bit unclear to me. It is currently described as a group where group dynamics principles such as team building are used to promote group cohesiveness, which to me doesn't really provide much clarity. I think it would help readers to provide an example here, as the definition to me doesn't really help make these two subtypes clearly distinct.

Response: Thank you for the suggestion. We have revised the definition for clarity. The revised text now reads:

> *"True groups were exercise classes where leaders intentionally apply group dynamics principles (e.g., establishing group goals, fostering team identity, using group feedback), to enhance cohesion and engagement. In contrast, non-true groups involve participants exercising together without these structured group-building strategies[23,24]."* (**page 4, lines 79–83)**

Comment 3: To some extent, the introduction appears to frame the COVID-19 pandemic and its associated measures as ongoing concerns. However, this perspective does not really align with the current global situation. Many countries have moved well beyond strict .pandemic protocols, and practices like social distancing are no longer widely enforced or promoted. To enhance the paper's longevity and broader applicability, I would suggest shifting the focus of the introduction away from specific COVID-19 impacts and focus it on more general issues where possible. This approach would not only make the paper more relevant to a wider range of scenarios but also prevent it from becoming quickly outdated as the world continues to move beyond COVID-19.

Response: We appreciate this suggestion. We have now revised the introduction to reduce emphasis on the pandemic as a central explanatory factor for altered physical activity modes. Instead, we present it as a catalyst that accelerated the adoption of new modalities, such as digital and home-based options. The revised text broadens the framing to highlight long-standing challenges and emerging opportunities since the Burke et al. meta-analysis, thereby enhancing the paper's relevance beyond the COVID-19 context. Specifically, we now state on **page 5, lines 93-101:**

> *" However, with technological developments and the introduction of online-delivered interventions[25], an updated understanding of the literature requires expanding the definition of a PA group to include online groups[26], and newer technologies such as virtual reality platforms[27]. These modalities were not widely accessible when Burke et al. conducted their review, potentially limiting the generalizability and scalability of their findings.*
>
> *This shift is particularly relevant considering the post-COVID increase in remote and home-based physical activity formats, which offer enhanced accessibility, especially for underserved or rural populations[25].."*

Comment 4: Unless it is a journal requirement, I would recommend that the authors consider placing the search strategy details before the eligibility criteria, as the former helps to give the latter a bit more context.

Response: Thank you for the stylistic suggestion. We have reviewed the structure and, unless journal formatting requires otherwise, we are happy to retain the current order, as it aligns with prior meta-analytic reviews and because the eligibility criteria guide the selection of appropriate search terms. . This is also the order recommended in the PRISMA checklist for systematic reviews. However, we remain open to reordering, if preferred by the editorial team.

Comment 5: I found myself wondering why conference proceedings and theses/dissertations were excluded? I think a strong defense is needed here since these are not recommend practices and can create publication bias in the results.

Response: Thank you for raising this important point. These sources were excluded to ensure the methodological quality and consistency of the included studies. We prioritized peer-reviewed publications that provided sufficient methodological detail and accessible data for effect size calculation. This decision is consistent with standards commonly applied in previous meta-

analyses and systematic reviews in the physical activity domain (e.g., Fong et al., 2012; Lee et al., 2025). Moreover, the inclusion of grey literature may increase heterogeneity and reduce methodological transparency, as such sources often contain interim or incomplete results and typically lack sufficient detail to adequately assess risk of bias (Gunnell et al., 2020).

We acknowledge that one of the main arguments for including grey literature in meta-analyses is to reduce the potential for publication bias (i.e., the selective publication of large or statistically significant effects; see Swift & Wampold, 2018). However, we believe this was less relevant in our case for two key reasons. First, the effect sizes synthesized in this meta-analysis were already relatively small, and most results were non-significant. Second, our assessment of publication bias (at least for the primary outcome) indicated that such bias was relatively low. Together, these findings suggest that the inclusion of grey literature would be unlikely to yield its primary benefit (i.e., mitigating inflated estimates due to publication bias), and may instead lower the overall quality of the synthesized evidence by introducing other sources of bias.

Fong, D. Y., Ho, J. W., Hui, B. P., Lee, A. M., Macfarlane, D. J., Leung, S. S., ... & Cheng, K. K. (2012). Physical activity for cancer survivors: Meta-analysis of randomised controlled trials. *BMJ*, *344*, e70. https://doi.org/10.1136/bmj.e70

Lee, S., Ahn, S., Patel, P., & Myers, N. D. (2025). A meta-analysis of eHealth interventions to promote physical activity in young, middle-aged, and late middle-aged adults with obesity or overweight [Published correction appears in *Obesity Reviews*, *26*(6), e13959]. *Obesity Reviews*, *26*(6), e13898. https://doi.org/10.1111/obr.13898

Gunnell, K., Poitras, V. J., & Tod, D. (2020). Questions and answers about conducting systematic reviews in sport and exercise psychology. *International Review of Sport and Exercise Psychology*, *13*(1), 297–318. https://doi.org/10.1080/1750984X.2019.1695141

Swift, J. K., & Wampold, B. E. (2018). Inclusion and exclusion strategies for conducting meta-analyses. *Psychotherapy Research, 28*(3), 356–366. https://doi.org/10.1080/10503307.2017.1405169

In practice, only one relevant conference abstract was identified during screening, and it lacked relevant or usable data. We now included this detail in the Method section **(Page 22, Lines 503-505):**

"Conference proceedings and theses/dissertations excluded due to limited methodological detail and incomplete data reporting. Only one relevant abstract was identified, which lacked usable data."

Comment 6: A minor point, but technically web of science and proquest are not actual databases, but rather platforms used to search databases. I would recommend framing the search around the papers that describe best practices for literature searching, since the

authors appeared to apply a lot of these best practices anyway. For instance, the following might help:

a. Harari, M. B., Parola, H. R., Hartwell, C. J., & Riegelman, A. (2020). Literature searches in systematic reviews and meta-analyses: A review, evaluation, and recommendations. Journal of Vocational Behavior, 118, 103377.

Response: Thank you for this helpful suggestion. We have corrected the terminology in the Methods section to reflect that we searched databases via platforms such as Web of Science and ProQuest. In addition, we now reference Harari et al. (2020) to frame our approach and acknowledge that our search strategy was developed in line with recommended best practices for systematic reviews and meta-analyses, including the use of multiple databases, backward citation tracking, and transparent inclusion criteria. Specifically, we now state **(Page 8, Lines 199-201):**

> "*Following best practices for systematic reviews and meta-analyses* ***(Harari et al., 2020)****, we conducted a comprehensive search of multiple databases via platforms such as Web of Science and ProQuest, ensuring broad coverage and methodological rigor.*"

Comment 7: While I am open to AI in streamlining some of the more procedural aspects of meta-analysis, as the authors have done here with the Research Screener Software, I think it would provide more weight to their review if they provided some evidence backing up that this is a valid tool and tends to result in reliable decisions – is there any evidence that can back up this tool?

Response: Thank you for raising this point. We have included citations to support the use of Research Screener as a valid and reliable tool. Specifically, a validation study by Chai et al. (2021) demonstrated that Research Screener performs comparably to manual screening in terms of both accuracy and efficiency. Further, a recent evaluation by Affengruber et al. (2024) found it to be among the most reliable of currently available automation tools.

We also note that our screening process exceeded current best-practice thresholds: while Chai et al. (2021) recommend manual review of approximately 35% of studies and Campos et al. (2024) suggest 20%, we manually reviewed 51% of records to ensure accuracy and rigor. These references and clarifications have been added to the Methods section where we state on **page 23, lines 528-533:**

> *"Using this tool, in the second step the reviewers screened 51% of the imported studies (3152/6217), manually reviewing those flagged as potentially eligible. This percentage far exceeded the 5%-35% recommended by the authors of the tool[50], as well as generic recommendations of a minimum of 20% for automated screening tools[51]. All AI-flagged potentially eligible abstracts were manually reviewed, and final inclusion decisions were verified by the two independent human reviewers (MK, DO)."*

References:

- Chai, Z., Polychronis, S., White, T., Liu, H., & Van Mierlo, T. (2021). Research Screener: A machine learning tool to semi-automate abstract screening for systematic reviews. *Systematic Reviews*, 10(1), 267. https://doi.org/10.1186/s13643-021-01792-1

- Affengruber, L., Wurth, S., Gartlehner, G., & Nussbaumer-Streit, B. (2024). Performance of machine learning tools for abstract screening in systematic reviews: Systematic evaluation. *BMJ Evidence-Based Medicine*. https://doi.org/10.1136/bmjebm-2023-112530

- Campos, R., Azevedo, R., Riegler, A., & Derboven, J. (2024). Guidelines for the responsible use of semi-automated tools in evidence synthesis. *Educational Psychology Review*. https://doi.org/10.1007/s10648-024-09862-5

Comment 8: Related to the above, it may also be worthwhile including some interrater reliability statistics in the screening decisions. Perhaps there is an opportunity to compute this for the human/AI decision points to test the accuracy of the decisions?

Response: Thank you for this thoughtful suggestion. We have now included interrater reliability statistics to enhance transparency. Specifically, we added the following to **page 23, lines 538–542:**

> *"Full texts of the studies deemed eligible after the title and abstract screening (n = 283) were retrieved and assessed for eligibility by at least two independent reviewers (MK, DO). Disagreements were resolved either through discussion (n = 55) or consultation with a third reviewer (HR, CTN or NN; n = 16). Agreement was reached in 213 cases (75%), and interrater reliability was moderate (Cohen's κ = 0.47)."*

Additionally, to clarify the role of the AI tool and human verification, we revised the Methods section (**page 23, lines 531–533**) and now state that:

> *"All AI-flagged potentially eligible abstracts were manually reviewed, and final inclusion decisions were verified by the two independent human reviewers (MK, DO)."*

This highlights our efforts to ensure accuracy and rigor in the screening process, while providing transparency regarding the interplay between AI and human decision-making.

Comment 9: A final point about screening is that I also found the section on title and abstract screening to be hard to follow, despite reading this paragraph multiple times. I would suggest revising to make things clearer and easier to follow.

Response: Thank you, for this observation. We have edited the paragraph in question to improve clarity. We also added additional information. (See above and in manuscript- **Page 23, Lines 528-533**).

Comment 10: I think a stronger defence could be provided about why both observational and experimental studies were aggregated together. Even though the effects were converted to a common metric, there is still the problem that these different study designs quantify different information. Thus, in my mind it only serves to make effect sizes difficult to interpret. Since there were only a handful of observational studies anyway, perhaps it would be a cleaner meta-analysis if only the experimental studies were included? In any case, a stronger defence is needed here.

Response: Thank you for this thoughtful comment. We made the a priori decision to include both experimental and observational studies in our main analysis because, prior to conducting the search, we were uncertain about the proportion of each study type and aimed to provide a comprehensive representation of the available literature. This approach aligns with established meta-analytic practices in behavioral science, particularly when the objective is to synthesize broad intervention effects across varied contexts.

That said, we fully acknowledge that experimental studies typically offer stronger evidentiary value than observational studies. For this reason, we conducted secondary analyses, for both primary and secondary outcomes, restricted to experimental and quasi-experimental studies **(see pages 14 and 16)**. These analyses yielded results that were substantively consistent with our main findings, suggesting that the inclusion of observational studies had minimal influence on the overall conclusions. In brief, removing the observational studies had minimal effect on our conclusions, while this action reduces by 20% the number of included studies. We have now made this point more explicit in the Discussion **(p. 19)** and have added corresponding information to the Methods section **(p. 13).**

Discussion- page 19, 427-436

> *"Although most studies were experimental, a small number were observational or cross-sectional, introducing potential limitations such as measurement imprecision and confounding. In line with our protocol, we aggregated both experimental and non-experimental studies[43]. Secondary analyses restricted to experimental and quasi-experimental studies yielded results consistent with the main analysis, suggesting that the inclusion of observational studies had minimal impact on overall findings. However, methodological concerns across the literature, including reliance on cross-sectional designs in some outcome domains, risk of bias, high heterogeneity, limited control for confounders, self-*

reported outcomes, use of indirect proxies (e.g., class attendance as a proxy for physical activity), and small sample sizes, indicate scope for methodological improvement in future research."

Method: Page 27-28, lines 636-643

"We made an a priori decision to include both experimental and observational studies to ensure comprehensive coverage of the literature and to reflect the full range of evidence on physical activity interventions across real-world contexts. While experimental designs typically provide greater evidentiary strength, observational studies can enhance ecological validity [49], To assess whether their inclusion biased results, we conducted a secondary analysis restricted to experimental and quasi-experimental studies, adjusted for pre-intervention physical activity levels. Results were consistent with the main analysis, indicating minimal impact on overall findings."

Comment 11: From page 22, the following statement: "Factors such as the predominance of cross-sectional studies, impression, risk of bias, high heterogeneity, lack of control for confounding, heavy reliance on self-reports for some outcomes (e.g., psychological) and indirect measures for others (e.g., class attendance for PA levels), and potentially underpowered studies, indicated that there is substantial room for improvement." was a bit confusing since most of the included studies in the meta-analysis were experimental, which seems at odds with this statement suggesting a predominance of cross-sectional studies. I would suggest revising for clarity.

Response: Thank you for pointing this out. We have revised the statement for clarity and accuracy (**page 19, lines 427-436**)

"Although most studies were experimental, a small number were observational or cross-sectional, introducing potential limitations such as measurement imprecision and confounding. In line with our protocol, we aggregated both experimental and non-experimental studies[43]. Secondary analyses restricted to experimental and quasi-experimental studies yielded results consistent with the main analysis, suggesting that the inclusion of observational studies had minimal impact on overall findings. However, methodological concerns across the literature, including reliance on cross-sectional designs in some outcome domains, risk of bias, high heterogeneity, limited control for confounders, self-reported outcomes, use of indirect proxies (e.g., class attendance as a proxy for physical activity), and small sample sizes, indicate scope for methodological improvement in future research."

Comment 12: Another limitation with the meta-analysis is that (from what I could tell) effects were not disattenuated for error in measurement. I don't think this is a confound, since it would affect both individual PA and group-based PA equally, but it would still give a more pure assessment of the effects. Something to consider.

Response: We thank the reviewer for this insightful observation. The reviewer is correct that we did not disattenuate effect sizes for measurement error. This decision was partly due to limitations in the meta-analytic software (metafor), which does not currently support this functionality. In addition, as the reviewer rightly notes, any measurement error is likely to have affected both individual and group-based PA conditions similarly and therefore would not be expected to introduce systematic bias between groups. We have now included this point as a limitation in the Discussion **(page 20, lines 461-464).**

> *"Finally, our obtained effect sizes were not disattenuated for measurement error. We suggest this is likely to have a minimal impact on results, since measurement error should affect both individual PA and group-based PA equally; however, we acknowledge that doing so would have provided a purer assessment of effects."*

We are grateful for the constructive feedback provided and believe the revisions have substantially strengthened the clarity, rigor, and practical relevance of our manuscript. We look forward to hearing your feedback on the revised version.

Reviewer 1 Comments

Overall Comments:
Thank you for the opportunity to review the revised version of "Manuscript #NATHUMBEHAV25010440A: Solo or team? A meta-analysis of individual vs. group-based studies on physical activity, functional, psychosocial, and health outcomes." The revision represents a significant improvement in both scope and clarity. The rationale for updating Burke et al.'s (2006) meta-analysis has been strengthened, methodological details are more transparent, and the discussion more effectively integrates theoretical frameworks and practical applications. These enhancements have made the manuscript substantially stronger and more relevant to both research and practice. At the same time, several areas still require further refinement to meet the highest standards of methodological rigor and interpretive precision. The following comments are intended to guide these final revisions.

Specific Comments:

1. Heterogeneity reporting: The authors should consider expanding their reporting of heterogeneity by including variance components from the three-level model, 95% prediction intervals, and estimates of the proportion of variance explained by moderators (e.g., pseudo-R^2). Adding omnibus tests of moderator significance and interpreting these results clearly would help readers better understand residual heterogeneity and the robustness of pooled estimates.

Response: We thank the reviewer for these detailed suggestions. We have now expanded our reporting of heterogeneity to provide a more complete account of between- and within-study variance. The **Results** section (pp. 9–10) now reports variance components from the three-level model, pseudo-R^2 values indicating the proportion of variance explained by moderators, and 95% prediction intervals for each outcome domain. These statistics and examples of the updated variance decomposition are detailed in Supplementary Material 3, which demonstrates the robustness of pooled estimates across domains.

2. *Outlier transparency: The authors should consider presenting side-by-side results with and without outliers across all outcome domains. Anchoring exclusion criteria in established influence diagnostics is helpful, but transparency would be further improved by including diagnostic plots or reproducible analytic details (e.g., code) in the Supplement. This would enhance replicability and reassure readers that conclusions are not contingent on analytic choices.*

Response: The analytic code is openly available on OSF https://osf.io/xt2g4. Consistent with the reviewer's recommendation, a ***Supplementary*** **File 3** now provides side-by-side pooled estimates with and without outliers for all outcome domains, as well as diagnostic plots (standardized residuals and Cook's distance). For example, the pooled physical-activity effect changed only marginally from g = 0.086 [95 % CI: –0.02, 0.21] to g = 0.092 [95 % CI: –0.01, 0.22] after outlier removal, indicating that our conclusions are robust to analytic decisions.

3. *Equivalence testing: The authors should consider explicitly defining and justifying a smallest effect size of interest as the basis for equivalence bounds. Clear justification for these bounds, drawn from prior literature or applied relevance, would bring equivalence testing in line with best practice. It would also be useful to consistently report both 90% confidence intervals and 95% confidence intervals (for traditional inference) and clarify whether equivalence conclusions are robust across sensitivity analyses.*

Response: We thank the reviewer for this valuable suggestion. We clarified the statistical approach to equivalence testing in the **Data Analyses** section (p. 28). Equivalence between group-based and individual interventions was assessed using the Two One-Sided Tests (TOST) procedure with bounds set at a smallest effect size of interest (SESOI) of ± 0.19 (see addition in manuscript, p. 28, lines 651-652) aligning with the 25th percentile of effect sizes observed at six-month follow-up in a meta-synthesis of physical-activity interventions (Wright et al., 2021). This is similar to our original bounds of ± .20, based on Cohen's effect size benchmarks, but more accurately reflects small effect sizes in the extant field. Both 90 % and 95 % confidence intervals are reported, and sensitivity analyses in the **Supplementary File 3** further confirm that equivalence conclusions are robust across analytic specifications.

4. *Publication Bias Diagnostics: The authors should consider extending their publication bias assessment beyond funnel plots and Egger's test by applying at least one additional method (e.g., a selection model, p-curve, or p-uniform analysis) for the primary outcome domain. This would provide additional assurance about the robustness of conclusions, particularly in light of potential small-study effects.*

Response: We again thank the reviewer for their excellent suggestion. We now extended our publication-bias assessment beyond funnel plots and Egger's test by implementing a z-curve analysis (**Results***,* p. 10; lines 209-214, Brunner & Schimmack, 2020, *Meta-Psychology*, 4- https://doi.org/10.15626/MP.2018.874). This method estimates the expected discovery rate

(EDR) and expected replication rate (ERR) if no publication bias was present. The analysis, presented in the **Supplementary 3***,* corroborates Egger's test findings and indicates minimal small-study effects across domains.

5. Protocol transparency: The authors should consider referencing a publicly available protocol (e.g., PROSPERO, OSF). If no public registration exists, providing a dated version of the protocol in the Supplement would enhance transparency. Any deviations from the original plan should also be noted explicitly.

Response: Our review was pre-registered on PROSPERO (https://www.crd.york.ac.uk/PROSPERO/view/CRD42021271452) which is now cited in the **Methods** (pp. 22, line 515). We confirm that our review followed the registered protocol for objectives, eligibility, and primary analyses, and included the following minor deviations/methodological enhancements:

1. Title wording was refined to better reflect the scope and outcome domains.
2. The search was extended beyond the original timeframe.
3. Only English-language studies were retained due to the absence of a second coder who spoke German
4. GRADE ratings were added
5. Equivalence testing (TOST) was added.
6. Psychological and social outcomes (listed separately in the protocol) were conceptually merged and reported as psychosocial outcomes to reflect the nature of the included measures and improve interpretability.
7. The original outcome category "physical health" was split into *functional* and *health* outcomes to match reporting in the primary studies.

These are now explicitly noted on page 22-23, lines 514-520.Full details of protocol adherence and deviations are listed in **Supplementary File 3***,* Table S5 (Protocol Adherence Summary).

6. Theoretical and Practical Integration: The authors should consider further strengthening the discussion by more explicitly connecting their findings to cost-effectiveness and equity considerations. In particular, the equivalence of online and in-person formats has important implications for scalable and resource-efficient delivery, which could be highlighted more directly to inform public health and policy debates.

Response: We thank the reviewer for this suggestion. The **Discussion** has now been expanded to more explicitly address cost-effectiveness and equity considerations. We now highlight the implications of equivalence between online and in-person formats for scalability and resource allocation. The revised text now states (p. 19, lines, 423-428):

> "The similar effectiveness of online and face-to-face groups across psychosocial, functional and PA-related outcomes supports the potential of online interventions to reach populations who face barriers to in-person participation, such as mobility limitations, transportation challenges, or geographic isolation. Online formats lower

> logistical demands (e.g., transport and staffing), expand scalability, and extend reach to underserved populations, thereby supporting both cost-effectiveness and equity.”

7. Limitations and Future Direction: The authors should consider expanding their limitations section to explicitly acknowledge the lack of adherence/retention data and the inconsistent reporting of intervention settings across primary studies. Highlighting these gaps would underscore important priorities for improving reporting standards in future research.

Response: We thank the reviewer for this point. Adherence and retention were generally well reported across the included studies and are therefore not a limitation in this dataset. We agree though that inconsistent reporting of intervention settings was a clear limitation. The **Limitations** section therefore now states:

> Although our sensitivity analyses indicated that the overall pattern of findings was largely robust to between-study differences, heterogeneity remains an important limitation. Inconsistent or incomplete reporting of intervention settings (e.g., community, workplace, clinical) and intervention characteristics (e.g., group size) prevented us from examining whether these factors moderated intervention effects. To improve interpretability and better isolate the conditions under which group or individual formats are most effective, future research should adopt clearer reporting standards for intervention context and consider standardizing key intervention characteristics or incorporating planned subgroup/moderator analyses. Such steps would enhance comparability across studies and improve the precision of effect size estimates. (p. 22, 475-483)

Reviewer 3 Comments

I commend the author team for their efforts in enhancing the paper and appreciate the thoughtful revisions that have been made. However, I believe there is one area that could benefit from further adjustment. You mention that unpublished sources were excluded to introduce an additional layer of methodological rigour to the studies. While I understand the rationale behind this decision, I suggest reconsidering this approach. Not all researchers are inclined to publish their findings in scientific journals, and there are cases where well-conducted studies remain unpublished. Conversely, poorly conducted studies can still find their way into publication. Thus, excluding unpublished sources might inadvertently overlook valuable insights and data or create bias in the results. Additionally, reducing heterogeneity is not a very good reason to exclude these sources as this approach does not address the root cause of heterogeneity and heterogeneity itself should simply reflect what is present in the literature.

I encourage the authors to at least acknowledge this possibility and the limitations of their approach in the limitations section, as in my mind this is by far the biggest limitation of the meta-analysis and it is not really addressed in much depth at all.

Other than that, the paper has improved, and I wish the authors all the best in their research

Response: We recognize the relevance of considering unpublished literature. As a comprehensive grey literature search at this stage is a very complex and time-intensive task, and with some co-authors recently taking on new senior academic roles, such a revision is practically unrealistic. Searching the literature, screening articles (primarily old student dissertations and conference abstracts), contacting authors (if their email addresses are still operational), updating all meta-analytic estimates (and coding for study quality), and updating the manuscript accordingly would need at least half a year, maybe more. Therefore, we are very appreciative that Reviewer 3 suggested the possibility of addressing this limitation in the limitations section.

We now explicitly acknowledge and discuss the exclusion of unpublished literature as a

limitation in the **Limitations** section (pp. 21–22, lines 489-493). The revised text now states:

> "In addition, we excluded grey/unpublished literature, including dissertations and conference proceedings, as these typically provide insufficient methodological detail or extractable data. While this decision aligns with our methodological rigor, it may bias results by overrepresenting published studies, which are more likely to report positive effects."

We sincerely thank the reviewers and editor for their helpful feedback, which has substantially strengthened our manuscript. We hope that our revisions satisfactorily address all concerns.
